# Electrochemical Deconstructive Methoxylation of Arylalcohols–A Synthetic and Mechanistic Investigation

**DOI:** 10.1002/chem.202403413

**Published:** 2024-10-30

**Authors:** Hussain A. Maashi, Toby Lewis‐Atwell, James Harnedy, Matthew N. Grayson, Louis C. Morrill

**Affiliations:** ^1^ Cardiff Catalysis Institute School of Chemistry Cardiff University Main Building, Park Place Cardiff CF10 3AT United Kingdom; ^2^ Department of Chemistry College of Science University of Bisha Bisha 61922 Saudi Arabia; ^3^ Department of Chemistry University of Bath Claverton Down, Bath BA2 7AY United Kingdom E-mails; ^4^ Department of Computer Science University of Bath Claverton Down, Bath BA2 7AY United Kingdom

**Keywords:** Organic electrochemistry, Deconstructive functionalization, Mechanistic studies

## Abstract

Herein, we report a mechanistic investigation of a recently developed electrochemical method for the deconstructive methoxylation of arylalcohols. A combination of synthetic, electroanalytical, and computational experiments have been performed to gain a deeper understanding of the reaction mechanism and the structural requirements for fragmentation to occur. It was found that 2‐arylalcohols undergo anodic oxidation to form the corresponding aromatic radical cations, which fragment to form oxocarbenium ions and benzylic radical intermediates via mesolytic cleavage, with further anodic oxidation and trapping of the benzylic carbocation with methanol to generate the observed methyl ether products. It was also found that the electrochemical fragmentation of 2‐arylalkanols is promoted by structural features that stabilize the oxocarbenium ions and/or benzylic radical intermediates formed upon mesolytic cleavage of the aromatic radical cations. With an enhanced understanding of the reaction mechanism and the structural features that promote fragmentation, it is anticipated that alternative electrosynthetic transformations will be developed that utilize this powerful, yet underdeveloped, mode of substrate activation.

## Graphical abstract

**Deconstructive Functionalization**: Herein, we report a mechanistic investigation of a recently developed electrochemical method for the deconstructive methoxylation of arylalcohols. A combination of synthetic, electroanalytical, and computational experiments have been performed to gain a deeper understanding of the reaction mechanism and the structural requirements for fragmentation to occur

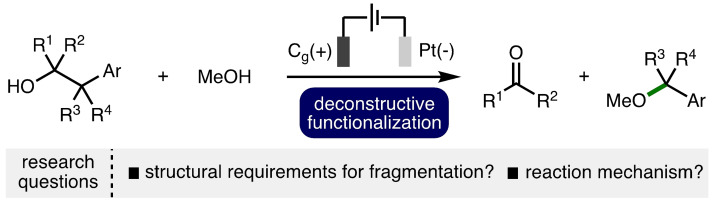

## Introduction

**Scheme 1:**
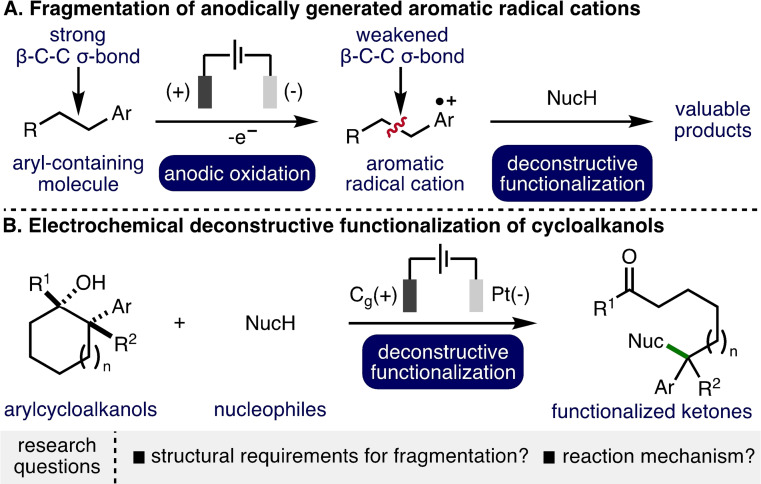
Context of this Investigation.

Organic electrochemistry offers a highly controllable and selective method to add to or remove electrons from molecules.^[1]^ By careful tuning of electrochemical parameters, specific single electron transfer processes can be targeted, accessing versatile reactive intermediates.^[2]^ Radical chemistry has a rich history, which showcases the application of various radical intermediates across a diverse range of useful and elegant transformations.^[3]^ The oxidation of aromatic rings to the corresponding aromatic radical cations results in the weakening of benzylic β−C−C σ‐bonds (Scheme 1A).[^4^, ^5^] This interesting and powerful mode of substrate activation has been applied towards the development of electrosynthetic methods,^[6]^ and particularly to the deconstructive functionalization of arylcyclopropanes,^[7]^ donor‐acceptor cyclopropanes/cyclobutanes,^[8]^ and 5‐, 6‐ and 7‐membered arylcycloalkanes.^[9]^ Analysis of these various electrosynthetic methods raises several intriguing mechanistic questions, including: i) which part of the molecule undergoes anodic oxidation (e. g., the aromatic nucleus or the alcohol functionality within arylcycloalkanols)?; ii) whether fragmentation (benzylic β−C−C σ‐bond cleavage) of the radical cation and nucleophilic attack occur in the same step (S_N_2‐like) or in distinct steps (S_N_1‐like); iii) the respective positions of the radical and cation upon fragmentation of the radical cation intermediates (if the S_N_1‐like mechanism is operative); iv) the key structural features (e. g., degree and type of substitution on the benzylic β−C−C σ‐bond) required for fragmentation to occur. Obtaining answers to these questions would facilitate rational reaction design.

In this domain, we recently developed an electrochemical approach for the deconstructive functionalization of unstrained cycloalkanols where various alcohols, carboxylic acids, and N‐heterocycles were employed as nucleophiles to generate a diverse array of synthetically useful remotely functionalized ketones (Scheme 1B).^[10]^ Based upon the scope and limitations of the reaction, the working mechanistic hypothesis involved an initial single‐electron anodic oxidation of the aromatic ring at either the 1‐ or 2‐position within the cycloalkanol substrate to give the corresponding aromatic radical cation. This species could be converted to a carbocation intermediate via mesolytic cleavage of the benzylic β−C−C σ‐bond with concomitant proton loss and single‐electron anodic oxidation, with subsequent attack by an external nucleophile to form the observed products. To gain a deeper understanding of this process and to address the aforementioned mechanistic questions, herein, we report an investigation into the electrochemical deconstructive functionalization of acyclic 2‐arylalcohols (and related substrates), which combines various synthetic, electroanalytical, and computational experiments.

## Results and Discussion

**Scheme 2:**
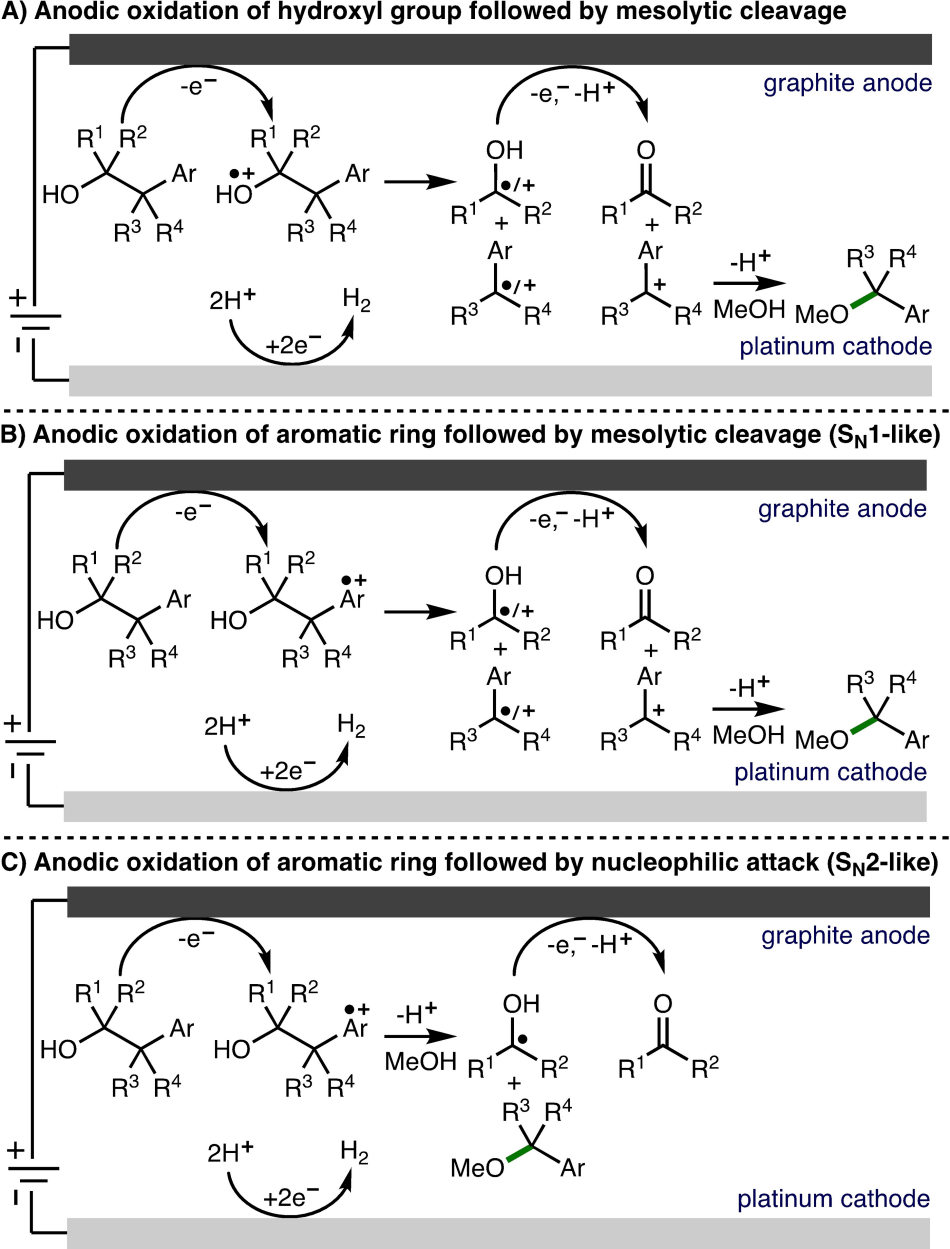
Mechanisms considered for C−C bond cleavage.

For this investigation, acyclic 2‐arylalcohols were selected as model substrates due to their commercial availability and/or expedient access via straightforward synthesis.^[11]^ Three mechanisms were considered for the C−C bond cleavage of 2‐arylalcohols: i) anodic oxidation of the hydroxyl group followed by mesolytic C−C bond cleavage (Scheme 2A); ii) anodic oxidation of the aromatic ring followed by mesolytic C−C bond cleavage (S_N_1‐like pathway) (Scheme 2B); iii) anodic oxidation of the aromatic ring followed by intermolecular nucleophilic attack (S_N_2‐like pathway) (Scheme 2C).

**Scheme 3:**
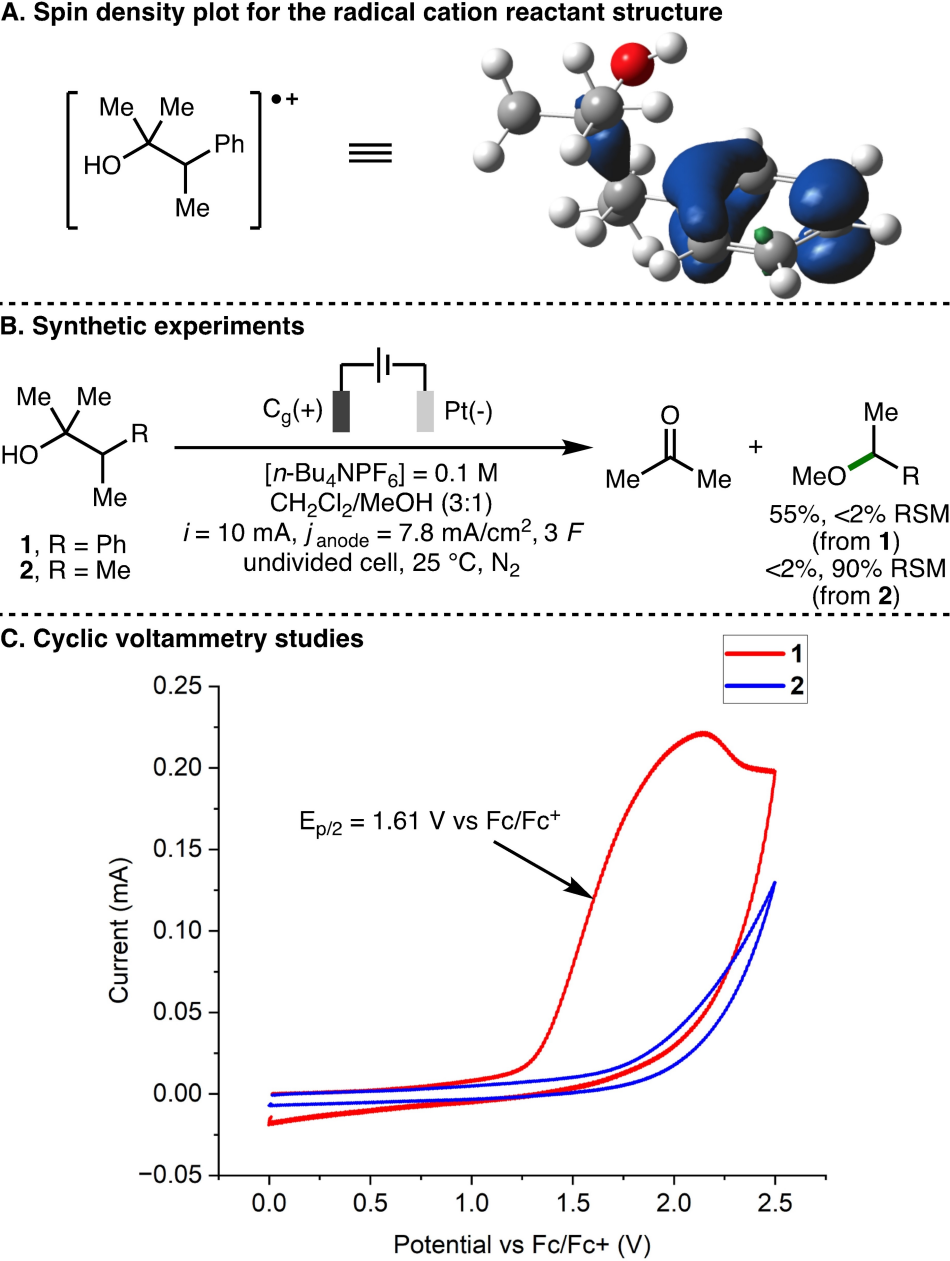
Spin density plot, synthetic, and cyclic voltammetry studies. RSM=recovered starting material. Yields as determined by ^1^H NMR analysis of the crude reaction mixture with 1,3,5‐trimethylbenzene as the internal standard. Spin density visualisation performed using GaussView 6.^[13]^

Initial efforts were directed towards investigating which motif within the 2‐arylalcohol scaffold undergoes anodic oxidation–the hydroxyl group, or the aromatic ring. We performed density functional theory (DFT) calculations at the ωB97X−D/def2‐TZVPP level of theory using Gaussian 16^[12]^ (see supporting information for full computational details). Visualization of the spin density plots for the radical cation reactant structure indicated that most of the radical character of the species exists on the aromatic ring (Scheme 3A). These calculations were complemented by synthetic studies, which revealed that subjecting 2‐arylalcohol **1** to the previously optimized electrochemical reaction conditions (graphite anode, platinum cathode, galvanostatic electrolysis (*i*=10 mA, *j*_anode_=7.8 mA/cm^2^, 3 *F*), *n*‐Bu_4_NPF_6_ as the supporting electrolyte in CH_2_Cl_2_/MeOH (3 : 1, [**1**]=0.05 M), in an undivided cell at 25 °C under N_2_), produced the deconstructive methoxylation product, (1‐methoxyethyl)benzene, in 55 % yield, whereas the corresponding aliphatic alcohol **2** was unreactive (Scheme 3B). Furthermore, cyclic voltammetry experiments revealed that 2‐arylalcohol **1** readily undergoes anodic oxidation (E_p/2_ = 1.61 V vs. Fc/Fc^+^) whereas aliphatic alcohol **2** does not undergo any observable oxidation in the same potential window (0–2.5 V vs. Fc/Fc^+^) (Scheme 3C). Taken together, these results indicate that the reaction mechanism likely initiates via anodic oxidation of the aromatic ring to form the corresponding aromatic radical cation.

**Scheme 4:**
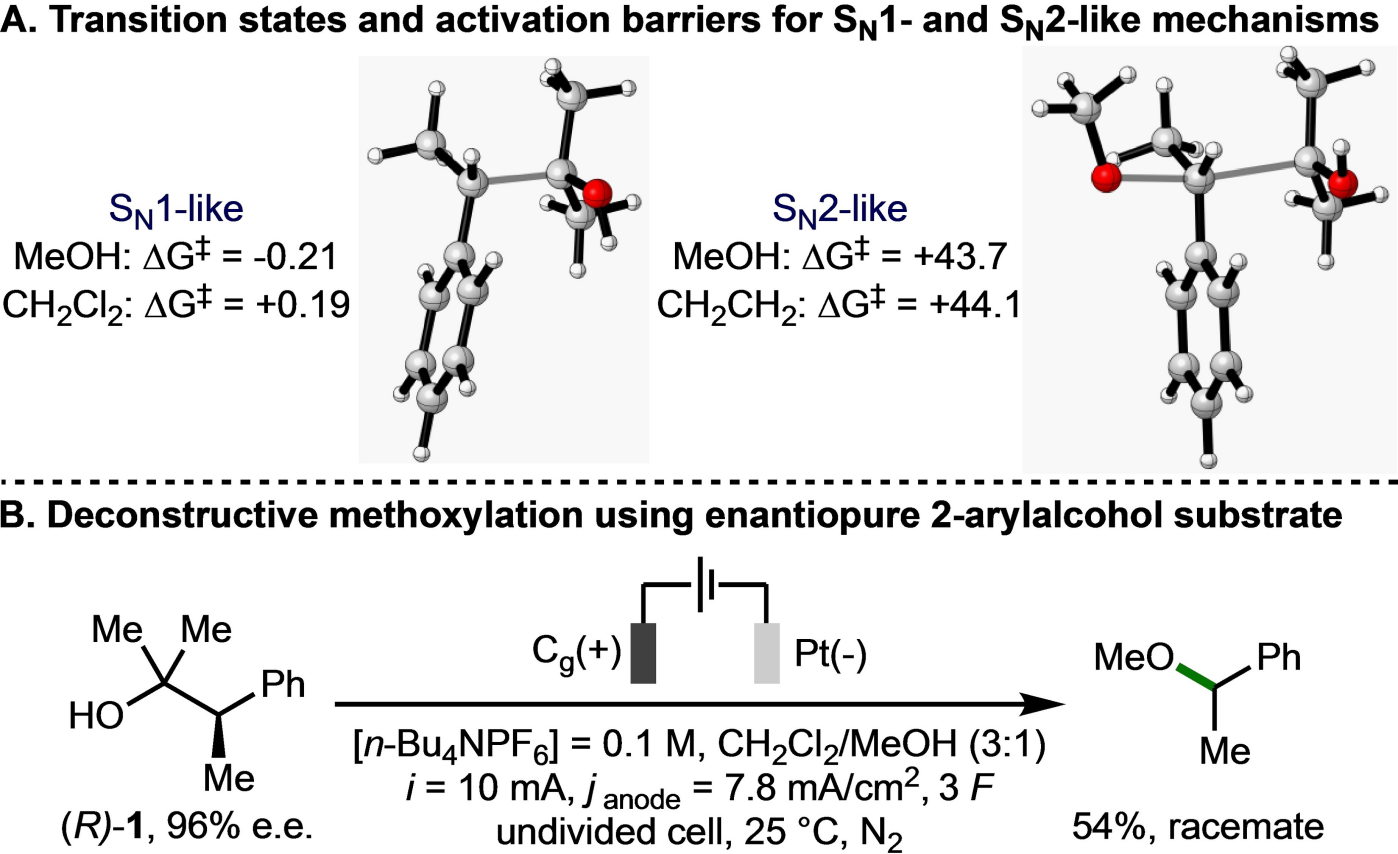
Investigation into S_N_1‐ and S_N_2‐like mechanisms. All energies in kcal/mol. Transition states were visualised in CYLview 1.0b.^[14]^

Next, focus was directed towards differentiating the two postulated mechanistic pathways for converting the anodically generated aromatic radical cation intermediate into the observed methyl ether products (Scheme 2B/C). The transition states for the S_N_1‐ and S_N_2‐like mechanisms were both located (Scheme 4A). The DFT activation barrier of the S_N_1‐like mechanism was calculated to be 0.19 kcal/mol in dichloromethane implicit solvent, and −0.21 kcal/mol in methanol implicit solvent. The activation barrier for the S_N_2‐like mechanism was calculated to be 44.1 and 43.7 kcal/mol in dichloromethane and methanol implicit solvents respectively. As such, the lower energy S_N_1‐like pathway is expected to be operative (c.f., Scheme 2B). This computational summation was reinforced by synthetic studies, which revealed that subjecting enantioenriched (*R*)‐2‐methyl‐3‐phenylbutan‐2‐ol **1** (96 % e.e.) to the electrochemical reaction conditions produced (1‐methoxyethyl)benzene in racemic form (Scheme 4B), which confirmed the involvement of a planar benzylic secondary carbocation intermediate in the reaction mechanism.

**Scheme 5:**
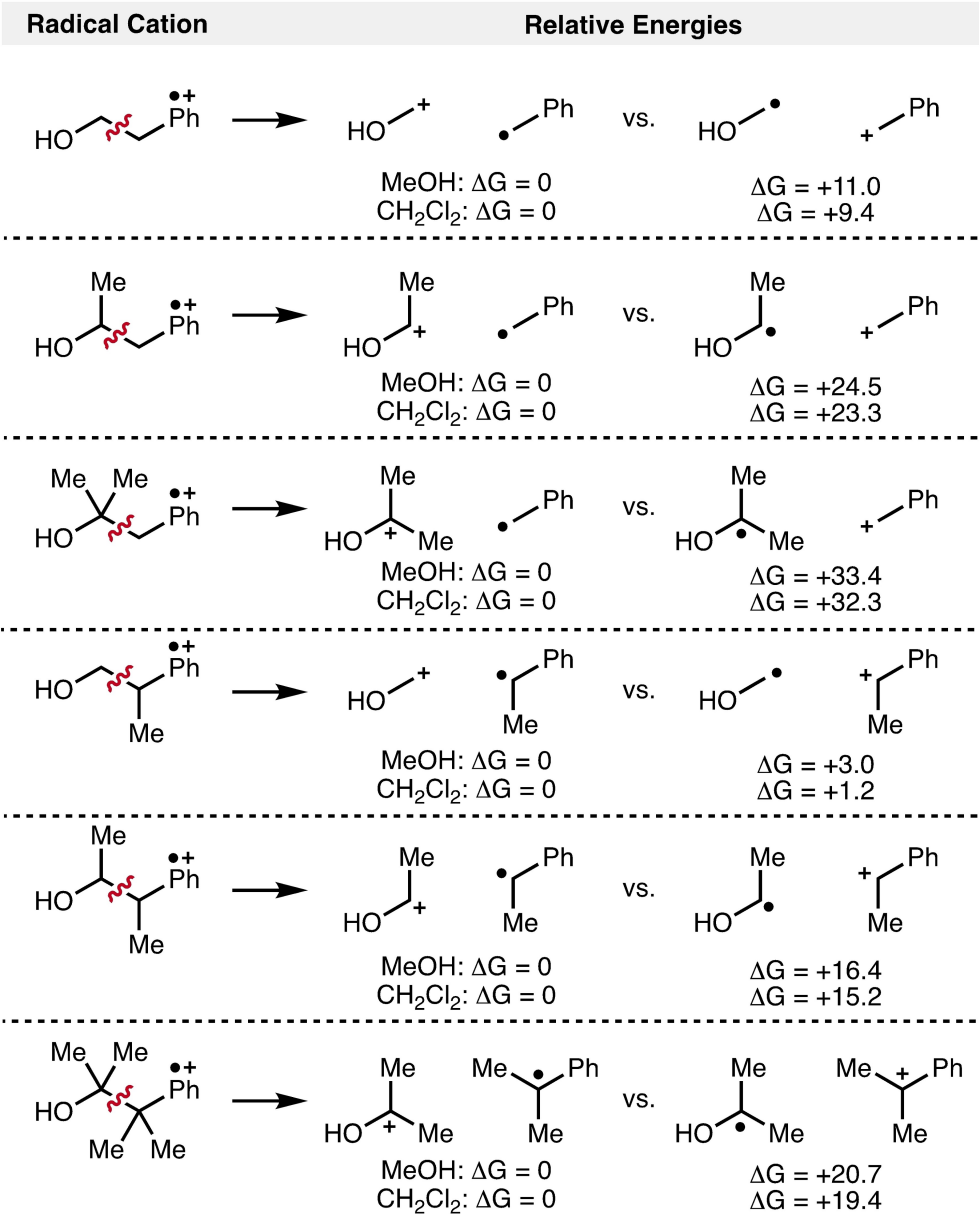
Relative stabilities of possible radical and carbocation fragments. All energies in kcal/mol.

Anodically generated aromatic radical cations from 2‐arylalcohols can undergo mesolytic cleavage to give two electronically distinct pairs of reactive intermediates, namely oxocarbenium ions and benzylic radicals, or α‐hydroxyl radicals and benzylic carbocations. To investigate the respective positions of the radical and cation upon fragmentation, the relative stabilities of the possible radical and cationic fragments that would be formed upon mesolytic cleavage of aromatic radical cations derived from various 2‐arylalcohols were calculated using DFT (Scheme 5). The thermodynamically favoured electronic arrangements are those that provide stabilization of the carbocation by electron‐donating hydroxyl groups (i. e., oxocarbenium ions), as well as stabilization of the radical intermediate from the presence of an aromatic ring (i. e., benzylic radicals). The difference in energy (ΔG) between the two electronically distinct pairs increases upon the formation of more substituted/stabilized oxocarbenium ions.

**Scheme 6:**
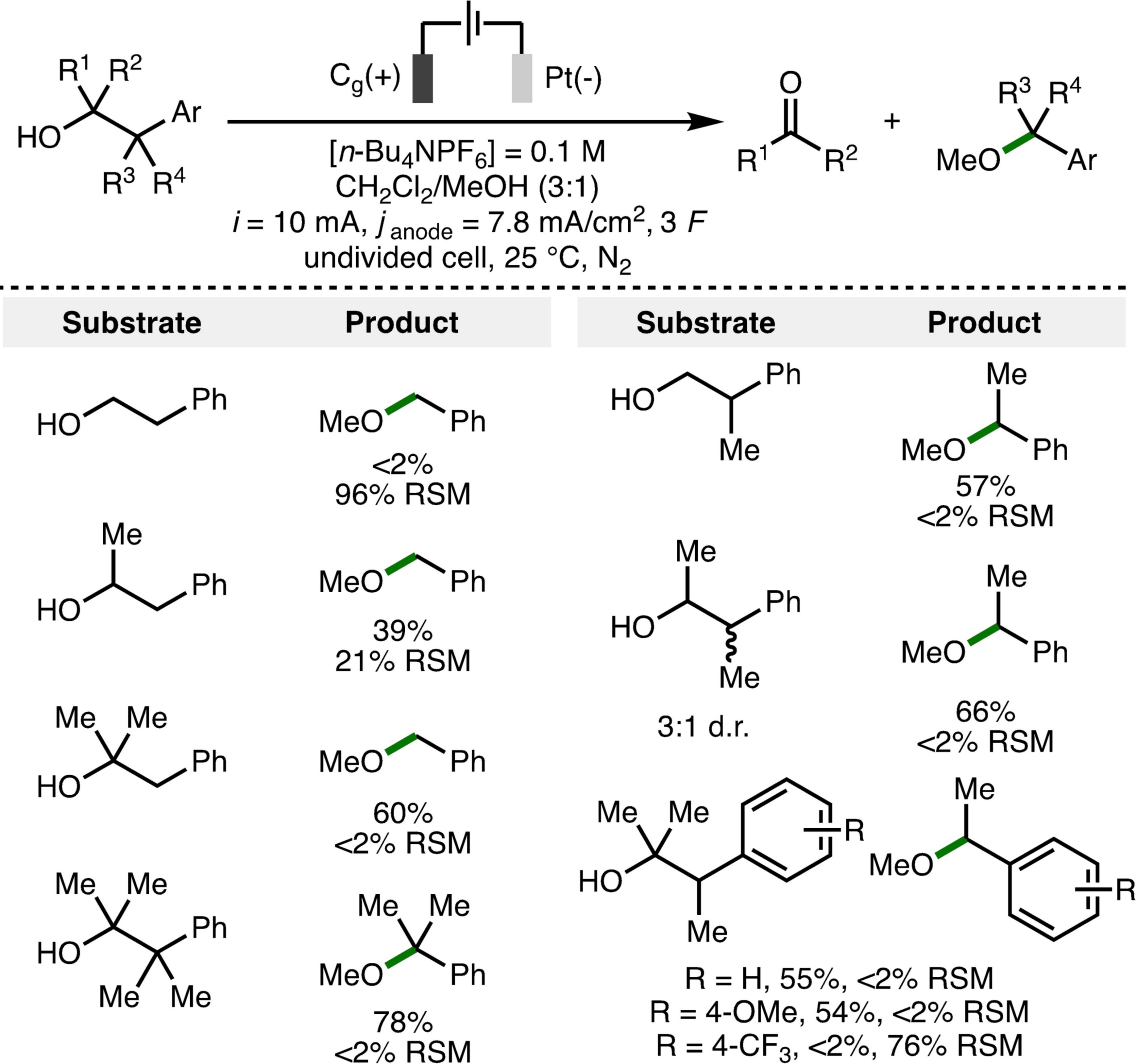
Deconstructive methoxylation of 2‐arylalcohols.

It was anticipated that the electrochemical deconstructive methoxylation would occur more readily using 2‐arylalcohols that contain additional substitution at the 1‐ and/or 2‐positions, due to the formation of more stabilized oxocarbenium ions and/or benzylic radical intermediates, resulting in a more energetically favoured mesolytic cleavage of the anodically generated aromatic radical cations. To probe this hypothesis, a selection of 2‐arylalcohol substrates were subjected to previously optimized electrochemical reaction conditions (Scheme 6). For 2‐arylalcohols that are proposed to undergo deconstructive methoxylation via the formation of primary benzylic radical intermediates (left column, top 3 rows), a clear trend in conversion to the corresponding methyl ether product, (methoxymethyl)benzene, was observed, with the 3° alcohol giving 60 % conversion, the 2° alcohol giving only 39 % conversion, whilst the 1° alcohol was found to be unreactive. For 2‐arylalcohols that contain an additional methyl group at the benzylic position (right column), it was found that 1°, 2° and 3° alcohols could each be converted to (1‐methoxyethyl)benzene in good yields. Taken together, these results support the hypothesis that the electrochemical fragmentation of 2‐arylalkanols requires structural features that stabilize the oxocarbenium ions and/or benzylic radical intermediates formed upon mesolytic cleavage of the aromatic radical cations. The impact of incorporating electron‐releasing (4‐OMe) and electron‐withdrawing (4‐CF_3_) aromatic substituents was also examined (right column, bottom row). It was found that 4‐OMe aryl substitution did not impact formation of the methoxy ether product (54 % yield), however, the formation of 4‐methoxyacetophenone was also observed (28 % yield), which is likely due to overoxidation of the methoxy ether product (E_p/2_ = 1.22 V vs. Fc/Fc^+^). It was also found that 4‐CF_3_ aryl substitution resulted in no observable conversion to the desired deconstructive methoxylation product, which may be attributed towards the higher oxidation potential of the substrate. As anticipated, a 2‐arylalcohol substrate that contained a fully substituted carbon chain (left column, bottom row) was converted into the corresponding methoxy ether product in high yield (78 %).

**Scheme 7:**
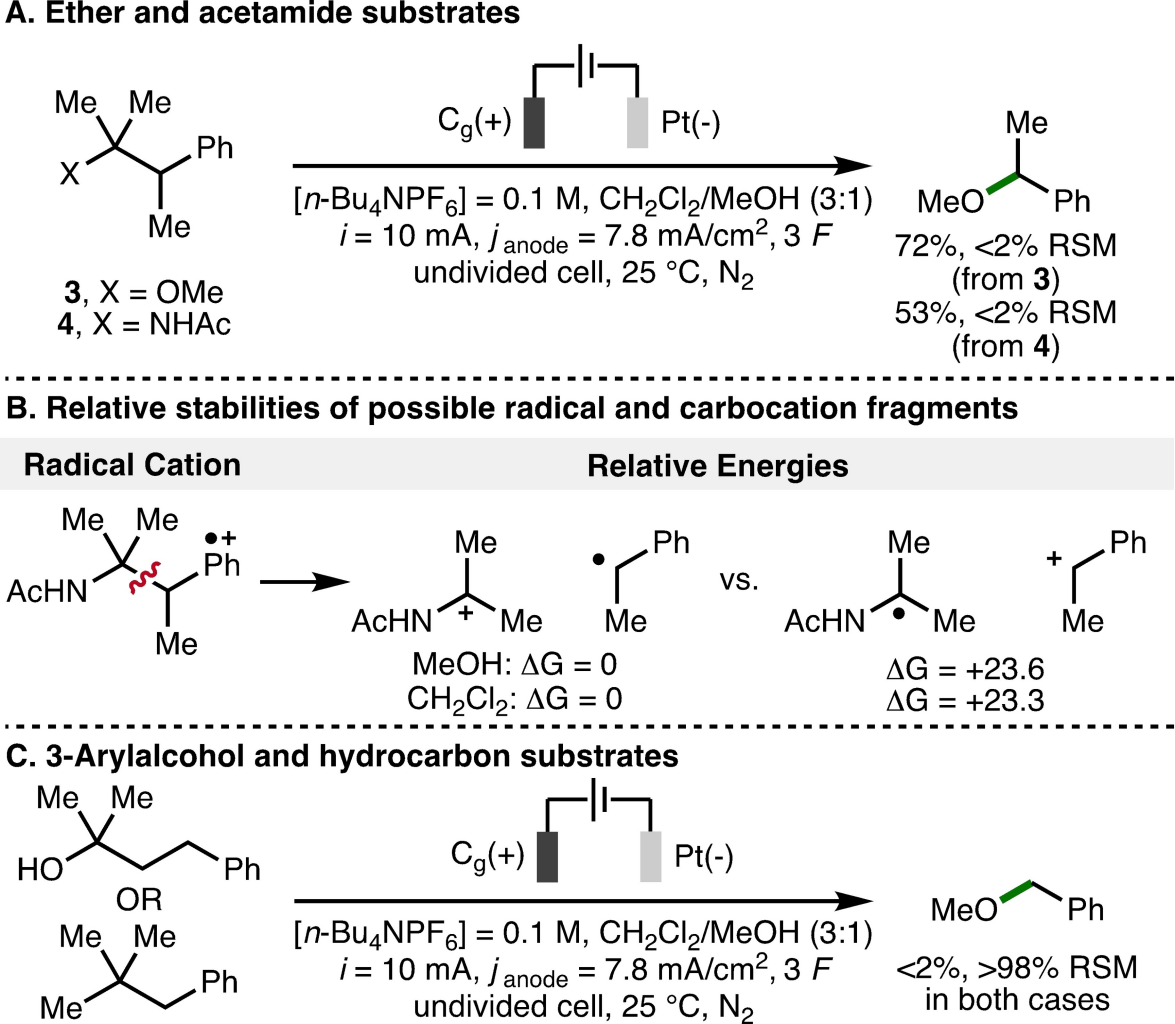
Alternative substrate studies. All energies in kcal/mol.

The impact of substituting the hydroxyl functional group with methyl ether and acetamide motifs upon the electrochemical deconstructive methoxylation process was also investigated (Scheme 7A). It was found that both substrates (3 and 4) could be converted to the same methyl ether product using the optimized electrochemical reaction conditions. The relative stabilities of the possible radical and cationic fragments that would be formed upon mesolytic cleavage of the aromatic radical cation derived from the 2‐arylacetamide substrate 4 were calculated using DFT (Scheme 7B). The lower energy electronic arrangement was found to be the acetamido carbocation and the benzylic radical intermediate. Furthermore, no deconstructive methoxylation product was observed when either a 3‐arylalcohol substrate or a hydrocarbon substrate were separately subjected to the electrochemical reaction conditions (Scheme 7C), which reinforced the requirement for substituents that would stabilize the proposed radical and cation intermediates to be formed upon mesolytic benzylic β−C−C σ‐bond cleavage of the anodically generated aromatic radical cations.

**Scheme 8:**
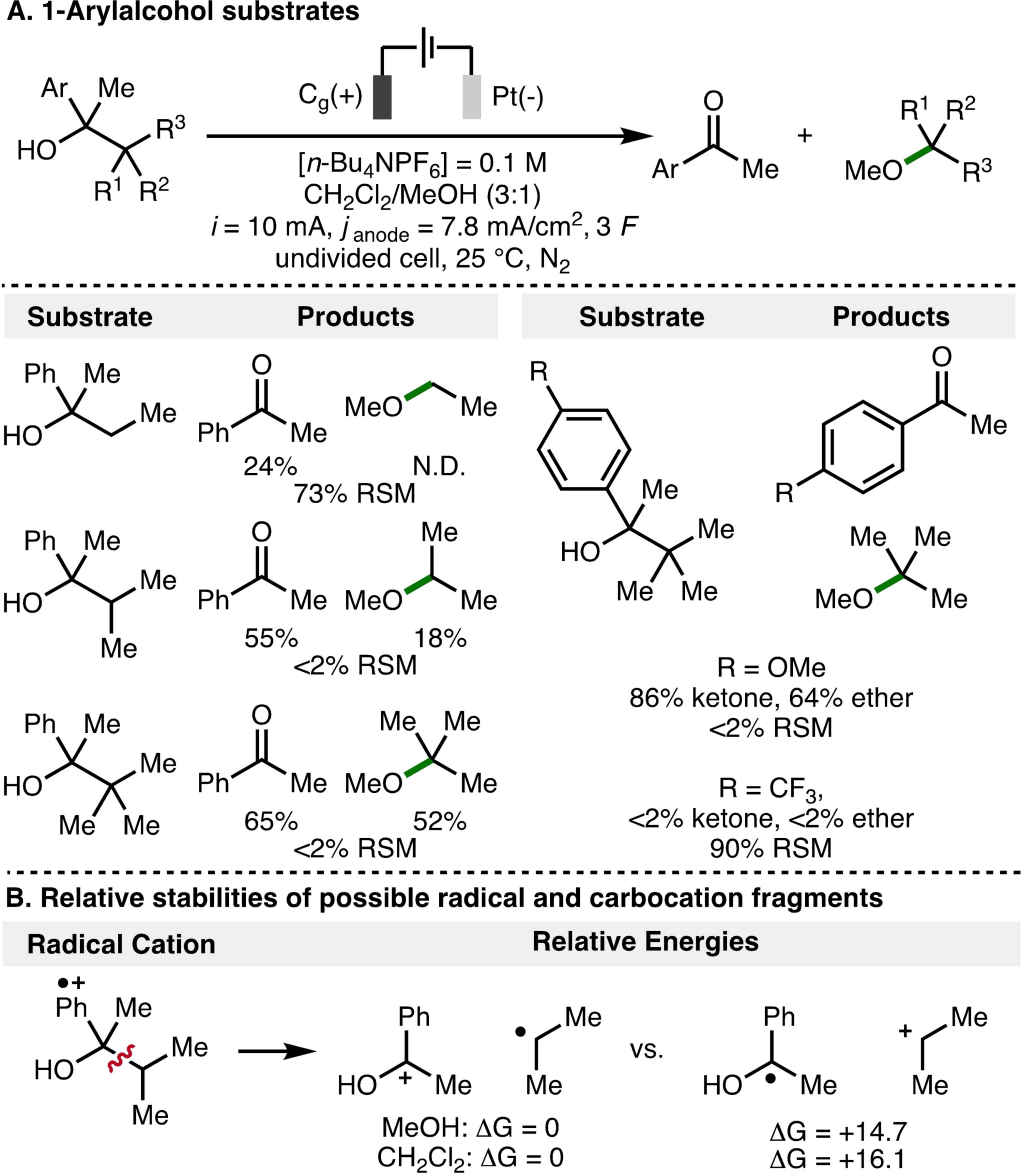
Investigation using 1‐arylalcohol substrates. N.D.=Not Determined. All energies in kcal/mol.

The electrochemical deconstructive methoxylation of a selection of 1‐arylalcohols was also investigated (Scheme 8A). Increased conversion of the 1‐arylalcohol substrates to acetophenone and the corresponding methyl ether products was observed upon increasing methyl substitution at the 2‐position within the 1‐arylalcohol scaffold (left column). The addition of an electron‐releasing 4‐OMe substituent on the aromatic ring further increased conversion, producing 4‐methoxyacetophenone and 2‐methoxy‐2‐methylpropane in 86 % and 64 % yields, respectively. However, a 1‐arylalcohol substrate that contained an electron‐withdrawing 4‐CF_3_ aromatic substituent, and hence has a higher oxidation potential, was unreactive using these electrochemical reaction conditions. The relative stabilities of the possible radical and cationic fragments that would be formed upon mesolytic cleavage of the aromatic radical cation derived from a model 1‐arylalcohol substrate were calculated using DFT (Scheme 8B), where the lower energy electronic arrangement was found to be the benzylic oxocarbenium ion and the alkyl radical intermediate. As such, the increased conversion observed for 1‐arylalcohol substrates that contain multiple methyl substitution at the 2‐position can be attributed towards formation of more stabilized alkyl radical intermediates, which undergo further anodic oxidation and trapping with methanol to form methyl ether products.

## Conclusions

In conclusion, we have performed a mechanistic investigation of the electrochemical deconstructive methoxylation of arylalcohols. A variety of synthetic, electroanalytical, and computational experiments have revealed that the reaction proceeds via anodic oxidation of 2‐arylalcohols to form the corresponding aromatic radical cation, which undergoes mesolytic cleavage of the weakened benzylic β−C−C σ‐bond to generate oxocarbenium and benzylic radical intermediates. Further anodic oxidation and trapping of the resulting benzylic carbocation with methanol provides access to the observed methyl ether products. The reactivity of various 2‐arylalcohol substrates were investigated to identify the structural features that promote electrochemical deconstructive methoxylation. It was found that groups which stabilize the oxocarbenium ions and/or benzylic radical intermediates, formed upon mesolytic cleavage of the aromatic radical cations, were required for efficient deconstructive methoxylation to occur. With an enhanced understanding of the reaction mechanism and the structural features that promote fragmentation, it is anticipated that alternative electrosynthetic transformations will be developed that utilize this powerful, yet underdeveloped, mode of substrate activation.

## Conflict of Interests

The authors declare no conflict of interest

1

## Supporting information

As a service to our authors and readers, this journal provides supporting information supplied by the authors. Such materials are peer reviewed and may be re‐organized for online delivery, but are not copy‐edited or typeset. Technical support issues arising from supporting information (other than missing files) should be addressed to the authors.

Supporting Information

## Data Availability

The data that support the findings of this study are openly available in the Cardiff University data catalogue at: https://doi.org/10.17035/d.2024.0325653068. Energies and Cartesian coordinates of all computed stationary points are available in the Supporting Information.
