# Supplementary material for: Electrochemical Deconstructive Methoxylation of Arylalcohols–A Synthetic and Mechanistic Investigation
Source: Chemistry. 2024 Oct 30;30(65):e202403413. doi: 10.1002/chem.202403413 (PMC11580601; doi:10.1002/chem.202403413)
Supplement: Supplementary file 1 — Supporting Information [file CHEM-30-e202403413-s001.pdf]

Chemistry–A European Journal

Supporting Information

Electrochemical Deconstructive Methoxylation of Arylalcohols–A Synthetic and Mechanistic Investigation

Hussain A. Maashi, Toby Lewis-Atwell, James Harnedy, Matthew N. Grayson,* and Louis C. Morrill*

Content

General Information 3

General Procedures 6

Substrate Synthesis and Characterisation 8

Electrochemical General Procedure and Products Interpretation 37

Cyclic Voltammetry Studies 53

Computational Studies 56

References 125

General Information

Unless otherwise stated, all non-electrochemical reactions were conducted in flame-dried glassware under an atmosphere of dry nitrogen, sealed with septum seals and were stirred with Teflon coated magnetic stirrer bars. Unless otherwise stated, all electrochemical reactions were performed using oven-dried 10 mL ElectraSyn vials under an atmosphere of dry nitrogen, sealed with an ElectraSyn Teflon cap fitted with a graphite anode and platinum cathode and were stirred with Teflon coated magnetic stirrer bars. Dry tetrahydrofuran (THF), diethyl ether (Et_2O) and acetonitrile (MeCN) and dichloromethane (CH_2Cl_2) were obtained after passing these previously degassed solvents through activated alumina columns (Mbraun, SPS-800). Tetra-*n*-butylammonium hexafluorophosphate (*n*-Bu_4NPF_6) was recrystallised from ethanol and dried in the oven before use. Spectroscopic grade anhydrous methanol was purchased and used for the electrochemical methoxylation. All other solvents and commercial reagents were used as supplied without further purification unless stated otherwise.

All electrochemical reactions were conducted using an ElectraSyn 2.0 apparatus, purchased from IKA. Graphite electrodes were purchased from IKA and are of uniform dimensions. Graphite electrodes were used as supplied from IKA or were cut from a sheet of carbon foil (2 mm thickness) purchased from Goodfellow. The electrodes were cut to the dimension of 8 mm × 52 mm using a Startrite Bandsaw (model 18-T-5) with a Starrett, Durate SFB high carbon steel blade (2870 mm x 10 mm x 0.65 mm, 3 mm pitch, regular tooth). Graphite electrodes could be used several times by renewing the top surface of the graphite. This was achieved by scraping away the top layer with a razor blade, sonicating in MeCN for 5 minutes, followed by oven drying for 30 mins. Platinum electrodes were cut from a sheet of platinum foil (0.05 mm thickness) purchased from Goodfellow using scissors to a width of 5 mm and to a standard length of an IKA supplied electrode (52 mm). Platinum electrodes were washed with water and acetone, then burned over a Bunsen burner before every reaction. The electrodes were set up in the standard IKA supplied Electrasyn vial cap with an electrode distance of 7 mm. Reactions were stirred at 400 rpm at room temperature.

Cyclic voltammetry (CV) experiments were conducted at room temperature using an Autolab PGSTAT204, controlled using Nova 2.1 software. The working electrode was a GC disc (3 mm dia., BASi part number MF-2012), the counter electrode was a Pt-wire (BASi part number MW-4130) and an Ag/AgCl reference electrode was used (BASi part number – MF-2052). The working electrode was polished on a water wet alumina pad in a figure of 8 motion for 30 seconds before being rinsed with deionised water and acetone. The counter electrode was rinsed with deionised water and acetone before being burned over a Bunsen burner. The scans were oxidative, and the oxidative potential window was (0.0 – 2.5 V vs. Fc/Fc^+), then referenced to Fc. The solvent was deoxygenated by bubbling through N_2 for 5 minutes before scan was initiated.

Room temperature (rt) refers to 20-25 °C. Ice/water and CO_2(s)/acetone baths were used to obtain temperatures of 0 °C and -78 °C respectively. All reactions involving heating were conducted using DrySyn blocks and a contact thermometer. *In vacuo* refers to reduced pressure through the use of a rotary evaporator.

Analytical thin layer chromatography (TLC) was carried out using aluminium plates coated with silica (Kieselgel 60 F254 silica) and visualisation was achieved using ultraviolet light (254 nm), followed by staining with a 1% aqueous $KMnO_4$ solution, or a 10% w/v solution of phosphomolybdic acid in ethanol. Flash column chromatography was performed using Kieselgel 60 silica in the solvent system stated using head-pressure by means of a compressed air line.

Melting points were recorded on an a Gallenkamp melting point apparatus and are reported corrected by linear calibration to benzophenone (47 - 49 °C) and benzoic acid (121 - 123 °C). Infrared spectra were recorded on a Shimadzu IRAffinity-1 Fourier Transform ATR spectrometer as thin films using a Pike MIRacle ATR accessory. The most intense peaks and structurally important peaks are quoted. Absorption maxima (ν_{max}) are recorded in wavenumbers (cm^{-1}). ^1H, ^{13}C and ^{19}F NMR spectra were obtained on a Bruker Avance 300 (300 MHz ^1H, 75 MHz ^{13}C), Bruker Avance 400 (400 MHz ^1H, 101 MHz ^{13}C, 376 MHz ^{19}F) or a Bruker Avance 500 (500 MHz ^1H, 126 MHz ^{13}C, 471 MHz ^{19}F) spectrometer at rt in the solvent stated. Chemical shifts are reported in parts per million (ppm) relative to the residual solvent signal. All coupling constants, *J*, are quoted in Hz. Multiplicites are reported with the following

symbols: br = broad, s = singlet, d = doublet, t = triplet, q = quartet, m = multiplet and combinations of these were used to denote higher order multiplicities. High resolution mass spectrometry (HRMS, m/z) data was acquired at Cardiff University. EI/CI HRMS data was collected on a Thermo Scientific Exactive GC machine with an orbitrap mass analyser. ES HRMS data was collected on a Walters Xevo G2XS machine with a TOF (Time of Flight) mass analyser.

“Petrol” and ‘’hexanes’’ refers to the fraction boiling in the range of 40-60 ^{o}C unless otherwise stated.

General Procedures

General Procedure A:

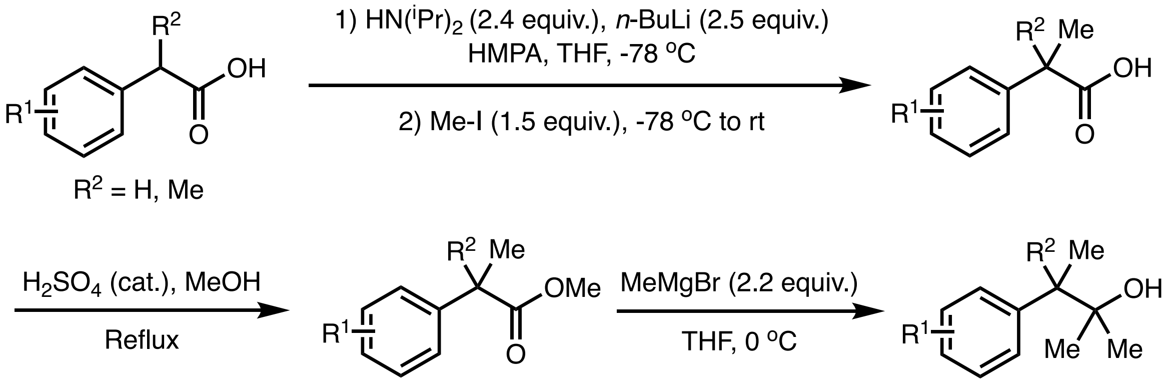

Step 1: To a flame dried flask was added diisopropylamine (2.5 equiv.) in THF (1 M) and the subsequent solution was cooled to -78 °C. *n*-butyllithium (2.4 equiv.) was added slowly and the mixture was stirred at -78 °C for 1 h. Arylacetic acid (1 equiv.) in HMPA (10 mL) was added slowly at -78 °C. After complete addition, the mixture was warmed up to 0 °C and stirred for 1 h. The mixture was cooled down to -78 °C and Iodomethane (1.5 equiv.) was added, and the mixture was allowed to stir up to room temperature overnight. The reaction mixture was quenched with 1 M HCl, extracted with ethyl acetate (2 x), organics combined, dried over $MgSO_4$, filtered and concentrated *in vacuo* yielding crude carboxylic acid.

Step 2: The crude residue was dissolved in MeOH (100 mL), concentrated H_2SO_4 (5 drops) was added, and the mixture heated to reflux for 5 h. The solvent was removed in vacuo, residue dissolved in EtOAc (100 mL), washed with a saturated solution of sodium bicarbonate (25 mL), organics dried over $MgSO_4$, filtered and concentrated in vacuo yielding crude methyl ester product. The crude residue was purified by flash column chromatography (eluent = EtOAc in hexanes, silica gel) yielding pure methyl ester product.

Step 3: To a flame dried flask was added methyl ester (1 equiv.) in THF (0.5 M) and the solution was cooled to 0 °C using an ice bath. Methylmagnesium bromide (2.2 equiv.) was added dropwise, and the mixture was allowed to stir up to room temperature overnight. The mixture was quenched with saturated NH_4Cl solution, extracted with EtOAc (2 x), organics combined, dried over $MgSO_4$, filtered and concentrated *in vacuo* yielding crude alcohol product. The

crude residue was purified by flash column chromatography (eluent = EtOAc in hexanes, silica gel) yielding pure alcohol product.

General Procedure B:

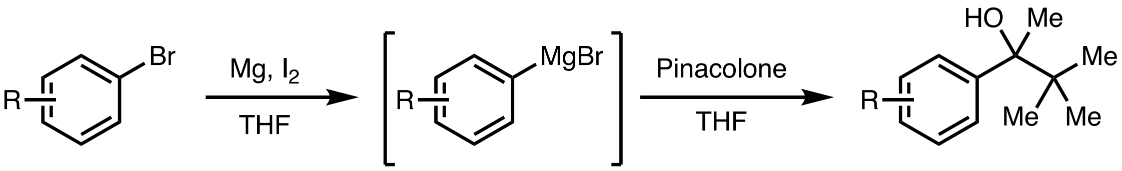

To a flame dried flask was added magnesium (1.8 - 2.4 equiv.), iodine (1 crystal) in THF (0.5 M). Arylbromide (1.5 - 2.0 equiv.) was added portionwise and the resultant black mixture was heated at reflux for 1-2 h. The mixture was cooled down to room temperature and 0 °C using an ice bath. Pinacolone (1 equiv.) in THF (1 M) was added and the mixture was stirred overnight. The mixture was quenched with saturated NH_4Cl solution, extracted with EtOAc (2 x), organics combined, dried over $MgSO_4$, filtered and concentrated *in vacuo* yielding crude alcohol product. The crude residue was purified by flash column chromatography (eluent = EtOAc in hexanes, silica gel) yielding pure alcohol product.

General Procedure C:

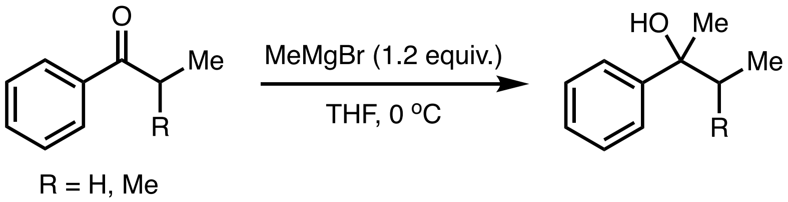

To a flame dried flask was added ketone (1 equiv.) in THF (0.5 M) and the solution was cooled to 0 °C using an ice bath. Methylmagnesium bromide (1.2 equiv.) was added dropwise, and the mixture was allowed to stir up to room temperature overnight. The mixture was quenched with saturated NH_4Cl solution, extracted with EtOAc (2 x), organics combined, dried over $MgSO_4$, filtered and concentrated *in vacuo* yielding crude alcohol product. The crude residue was purified by flash column chromatography (eluent = EtOAc in hexanes, silica gel) yielding pure alcohol product.

Substrate Synthesis and Characterisation

Sourced Commercially:

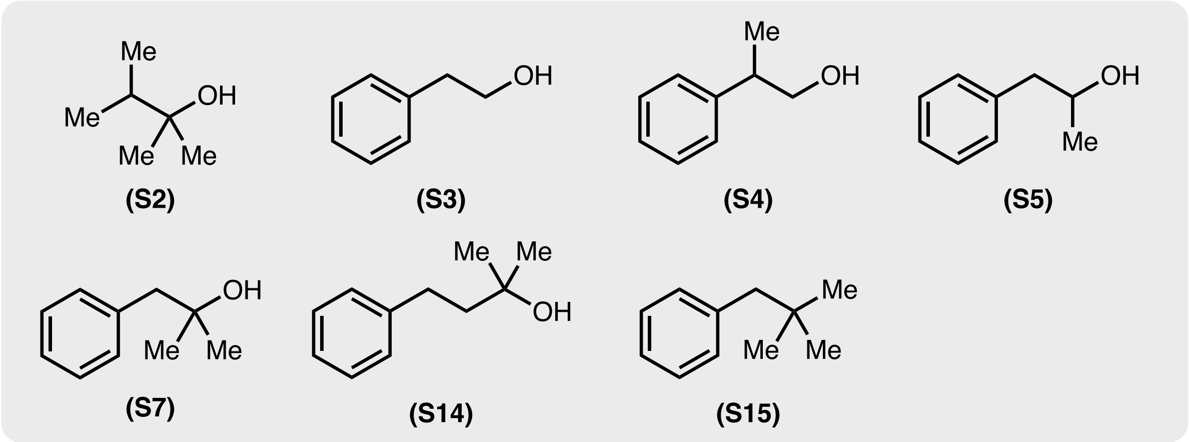

(S1)

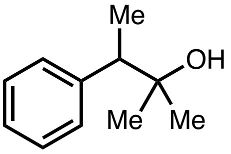

Prepared according to General Procedure A, step 3, using methyl 2-phenylpropanoate (3.28 g, 20 mmol), THF (40 mL) and methylmagnesium bromide (14.7 mL, 44 mmol, 2.2 equiv., 3M in Et_2O). The crude residue was purified by flash column chromatography (eluent = 5 to 10% EtOAc in hexanes, silica gel) to afford product as a white solid (2.82 g, 86% yield) as a racemate.

Mp.: 49-52 °C; **R_f** = 0.29 (eluent = 20% EtOAc in hexanes); **^{1}H NMR (300 MHz, $CDCl_3$)** δ 7.34 – 7.23 (m, 5H), 2.84 – 2.77 (q, *J* = 7.2 Hz, 1H), 1.36 – 1.33 (d, *J* = 7.2 Hz, 3H), 1.18 (s, 6H); **^{13}C NMR (126 MHz, $CDCl_3$)** δ 143.5, 129.1, 128.2, 126.7, 72.7, 50.5, 28.3, 27.0, 15.9.

Data consistent with the literature.[1]

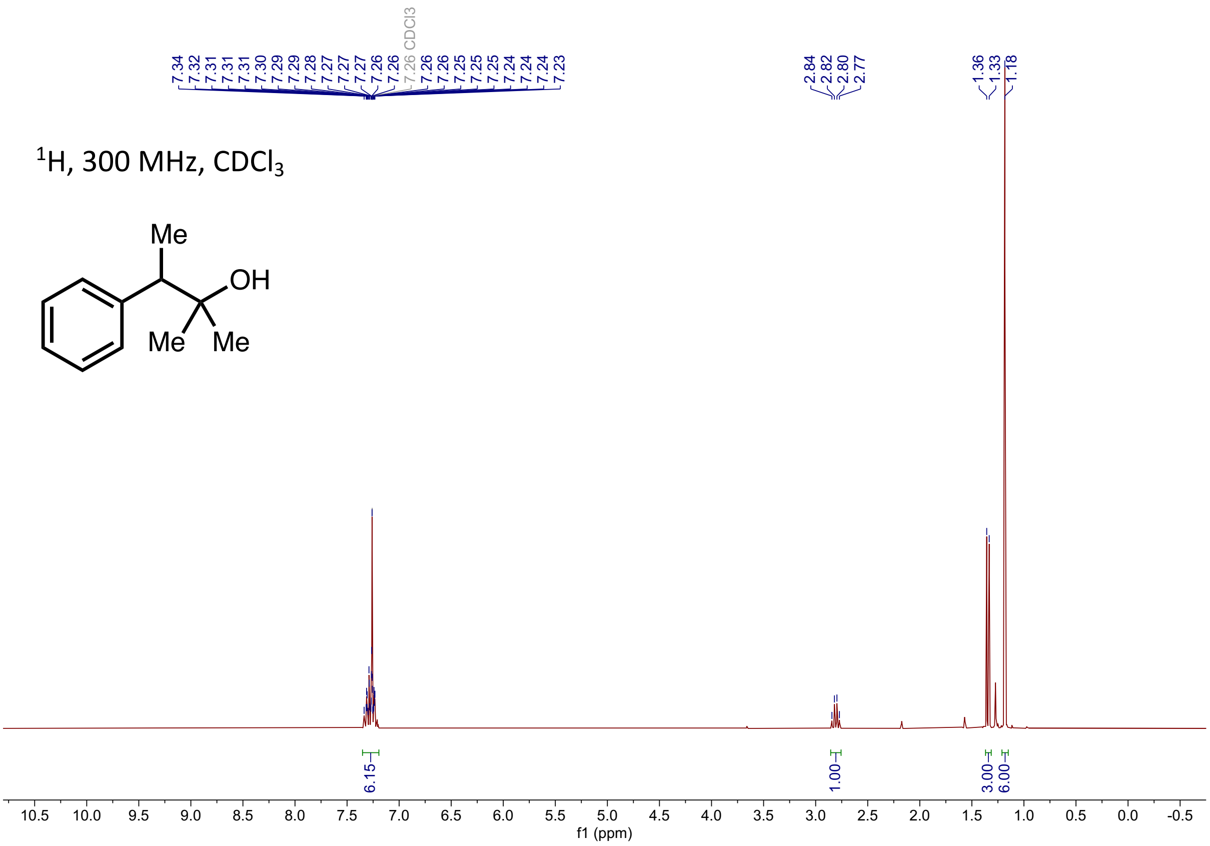
7.34 7.32 7.31 7.31 7.30 7.29 7.29 7.28 7.27 7.27 7.26 7.26 7.26 CDCl3 7.26 7.25 7.25 7.24 7.24 7.24 7.23
2.84 2.82 2.80 2.77
1.36 1.33 1.18
^{1}H, 300 MHz, $CDCl_3$
Me
OH
Me
Me
6.15
1.00
3.00
6.00
10.5 10.0 9.5 9.0 8.5 8.0 7.5 7.0 6.5 6.0 5.5 5.0 4.5 4.0 3.5 3.0 2.5 2.0 1.5 1.0 0.5 0.0 -0.5
f1 (ppm)

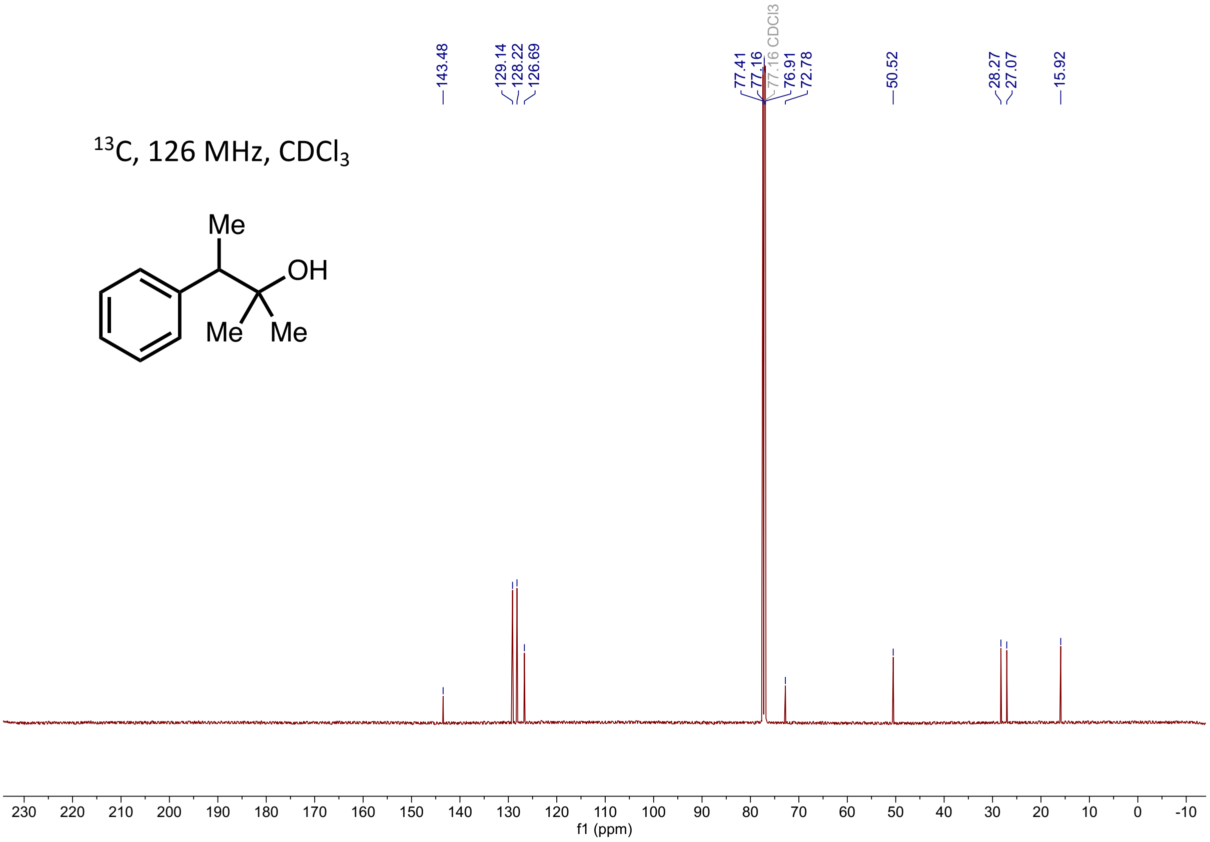
143.48
129.14 128.22 126.69
77.41 77.16 77.16 CDCl3 76.91 72.78
50.52
28.27 27.07
15.92
^{13}C, 126 MHz, $CDCl_3$
Me
OH
Me
Me
230 220 210 200 190 180 170 160 150 140 130 120 110 100 90 80 70 60 50 40 30 20 10 0 -10
f1 (ppm)

	Inj. Number	Peak Name	R. Time	Area	Area %
1	1.00	*1	7.39	532106.69	49.43
2	1.00	2	10.13	544347.50	50.57

HPLC Chiralpak IA column, 25 °C, 95:5 Hexane/IPA, 1 mL/min, 60 min; *S* = 7.4 min *R* = 10.1 min

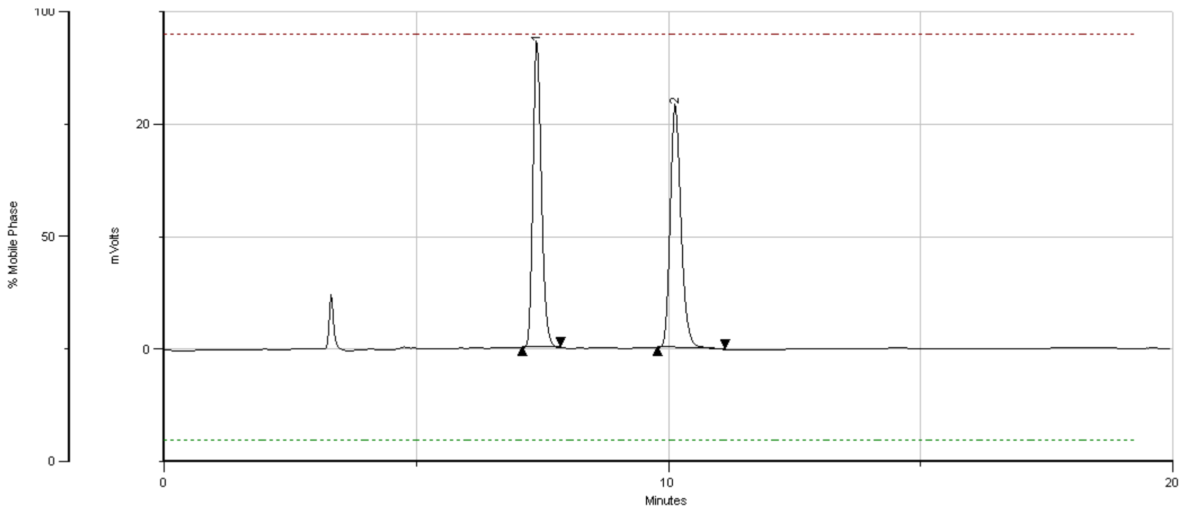

Figure S1. *HPLC trace for racemic* ***S1****.*

(*R*)-(S1)

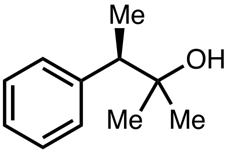

Prepared according to General Procedure A, step 3, using methyl (*R*)-2-phenylpropanoate (821 mg, 5 mmol), THF (10 mL), Methylmagnesium bromide (3.7 mL, 11 mmol, 2.2 equiv., 3M in Et_2O). The crude residue was purified by flash column chromatography (eluent = 5 to 10% EtOAc in hexanes, silica gel) to afford product as a white solid (665 mg, 81% yield).

Mp.: 45-48 °C; **R_f** = 0.29 (eluent = 20% EtOAc in hexanes); **v_{max} / cm^{-1}** (thin film) 3315, 2987, 2968, 1446, 1371, 1143, 948, 867, 769, 702; **^{1}H NMR (300 MHz, $CDCl_3$)** δ 7.34 – 7.20 (m, 5H), 2.84 – 2.77 (q, *J* = 7.2 Hz, 1H), 1.33 – 1.36 (d, *J* = 7.2 Hz, 3H), 1.18 (s, 6H); **^{13}C NMR (126 MHz, $CDCl_3$)** δ 143.5, 129.1, 128.2, 126.7, 72.8, 50.5, 28.3, 27.0, 15.9; **HRMS (EI$^+$)** [$C_{11}H_{16}O$] requires $[M-CH_3]^+$ 149.0971, found 149.0959 (- 1.58 ppm).

Data consistent with the literature.[2]

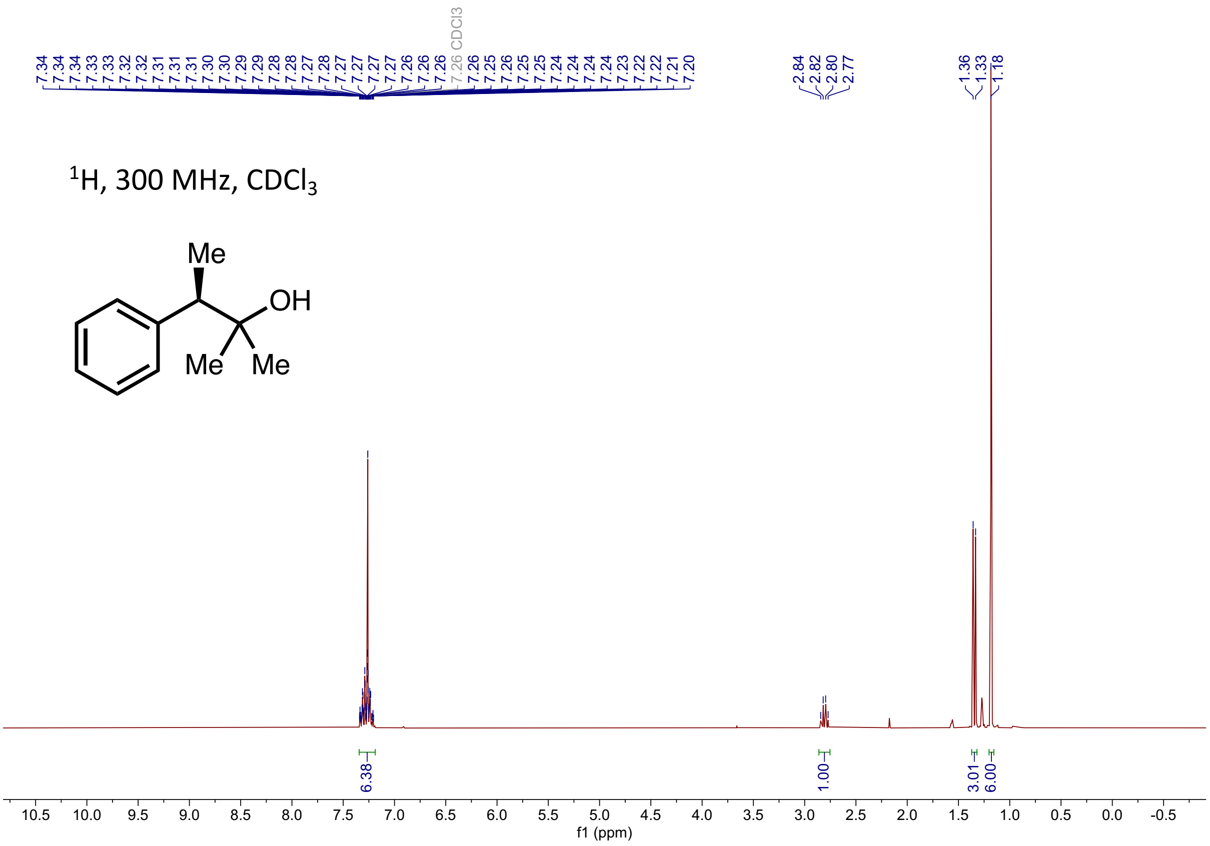
7.34
7.34
7.34
7.33
7.33
7.32
7.32
7.31
7.31
7.31
7.30
7.30
7.29
7.29
7.28
7.28
7.28
7.27
7.27
7.27
7.27
7.26
7.26
7.26
7.26 CDCl3
7.26
7.25
7.26
7.25
7.25
7.24
7.24
7.24
7.24
7.23
7.22
7.22
7.21
7.20
2.84
2.82
2.80
2.77
1.36
1.33
1.18
¹H, 300 MHz, CDCl₃
Me
OH
Me
Me
6.38
1.00
3.01
6.00
10.5 10.0 9.5 9.0 8.5 8.0 7.5 7.0 6.5 6.0 5.5 5.0 4.5 4.0 3.5 3.0 2.5 2.0 1.5 1.0 0.5 0.0 -0.5
f1 (ppm)

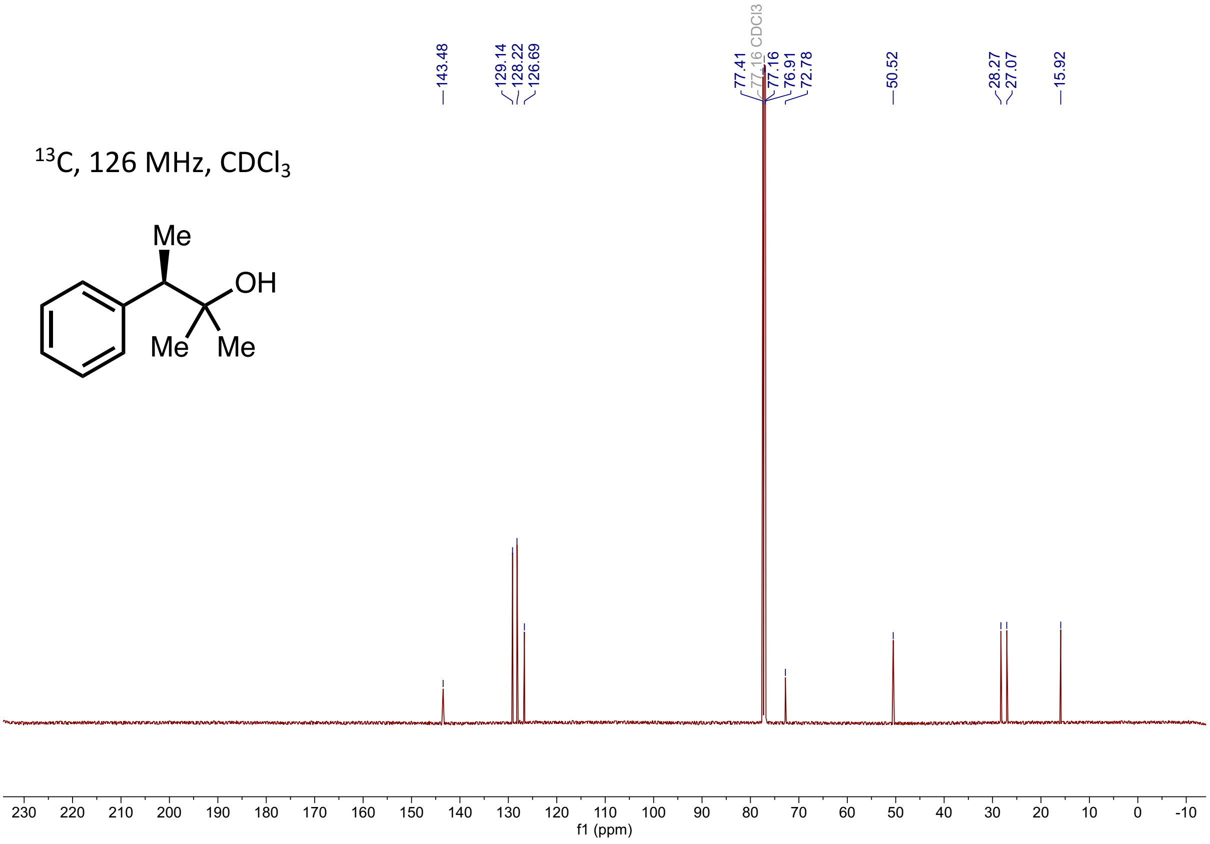
143.48
129.14
128.22
126.69
77.41
77.16 CDCl3
77.16
76.91
72.78
50.52
28.27
27.07
15.92
¹³C, 126 MHz, CDCl₃
Me
OH
Me
Me
230 220 210 200 190 180 170 160 150 140 130 120 110 100 90 80 70 60 50 40 30 20 10 0 -10
f1 (ppm)

	Inj. Number	Peak Name	R. Time	Area	Area %
1	1.00	*1	7.48	21866.67	1.76
2	1.00	*2	10.03	1221818.25	98.24

HPLC Chiralpak IA column, 25 °C, 95:5 Hexane/IPA, 1 mL/min, 60 min; *S* = 7.5 min, *R* = 10 min, e.e. = 96%

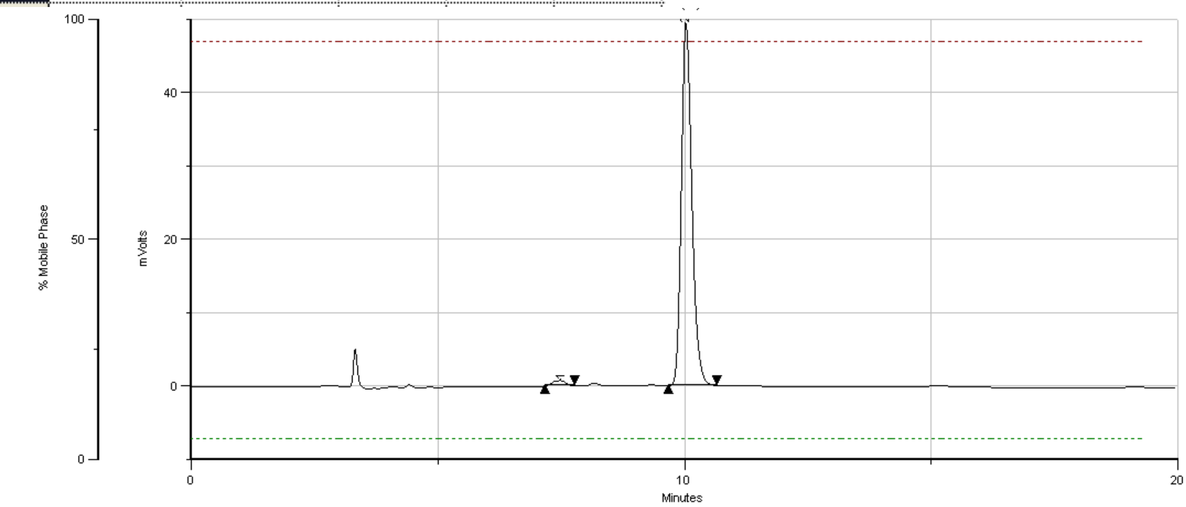

Figure S2. *HPLC trace for* ***(R)-S1****.*

(S6)

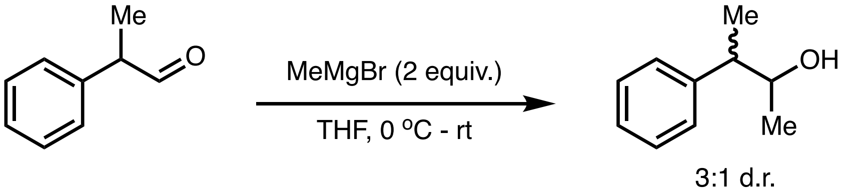

To a flame dried flask was added 2-phenylpropanal (2.68 g, 20 mmol, 1 equiv.) and THF (40 mL) and the solution was cooled to 0 °C using an ice bath. Methylmagnesium bromide (13.4 mL, 40 mmol, 2 equiv., 3M in Et_2O) was added dropwise, and the mixture was allowed to stir up to room temperature overnight. The mixture was quenched with saturated NH_4Cl solution (50 mL), extracted with EtOAc (2 x 50 mL), organics combined, dried over $MgSO_4$, filtered and concentrated *in vacuo* yielding crude alcohol product. The crude residue was purified by flash column chromatography (eluent = 5 to 10% EtOAc in hexanes, silica gel) to afford product as a colourless oil (1.84 g, 61% yield) as a 3:1 mixture of diastereomers.

R_f = 0.31 (eluent = 20% EtOAc in hexanes)

Selected data for major diastereomer:

^{1}H NMR (500 MHz, $CDCl_3$) δ 2.7 – 2.64 (m, *J* = 6.9 Hz, 1H), 1.27 – 2.25 (d, *J* = 7.0 Hz, 3H), 1.03 – 1.02 (d, *J* = 6.3 Hz, 3H); **^{13}C NMR (126 MHz, $CDCl_3$)** δ144.3, 128.6, 128.0, 126.6, 72.5, 47.3, 21.2, 16.1.

Selected data for minor diastereomer:

^{1}H NMR (500 MHz, $CDCl_3$) δ 2.64 – 2.58 (m, J = 7.3 Hz, 1H), 1.21 – 1.19 (d, *J* = 7.1 Hz, 3H), 1.17 – 1.16 (d, *J* = 6.2 Hz, 3H); **^{13}C NMR (126 MHz, $CDCl_3$)** δ143.7, 128.8, 128.2, 126.9, 72.5, 48.1, 20.7, 18.0.

Selected data for both diastereomers:

^{1}H NMR (500 MHz, $CDCl_3$) δ 7.29 – 7.10 (m, 5H), 3.86 – 3.75 (m, 1H).

Data consistent with the literature.[3]

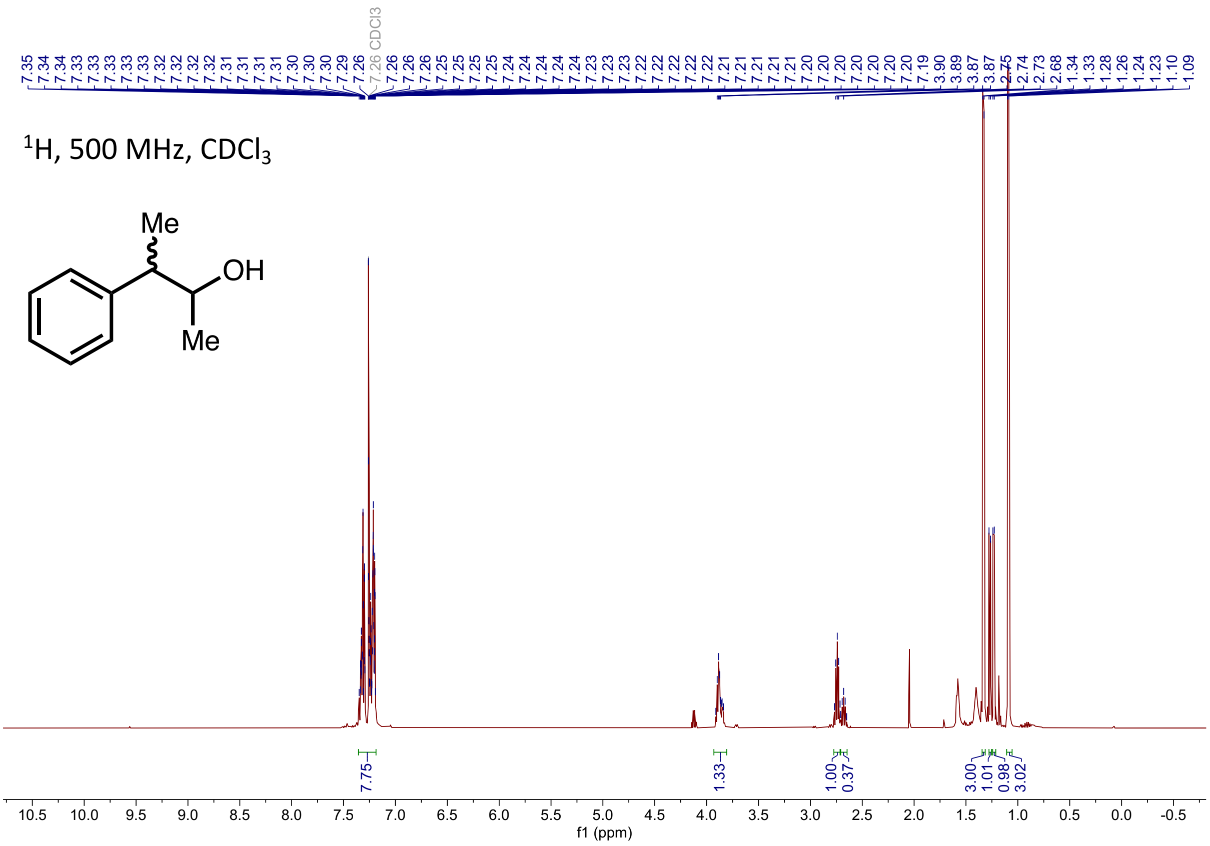
7.35 7.34 7.33 7.33 7.33 7.33 7.32 7.32 7.32 7.31 7.31 7.31 7.30 7.30 7.30 7.29 7.26 7.26 CDCl3 7.26 7.26 7.25 7.25 7.25 7.24 7.24 7.24 7.24 7.23 7.23 7.23 7.22 7.22 7.22 7.22 7.22 7.21 7.21 7.21 7.21 7.20 7.20 7.20 7.20 7.20 7.20 7.19 3.90 3.89 3.87 3.87 2.75 2.74 2.73 2.68 1.34 1.33 1.28 1.26 1.24 1.23 1.10 1.09
1H, 500 MHz, CDCl3
Me
OH
Me
7.75
1.33
1.00
0.37
3.00
1.01
0.98
3.02
10.5 10.0 9.5 9.0 8.5 8.0 7.5 7.0 6.5 6.0 5.5 5.0 4.5 4.0 3.5 3.0 2.5 2.0 1.5 1.0 0.5 0.0 -0.5
f1 (ppm)

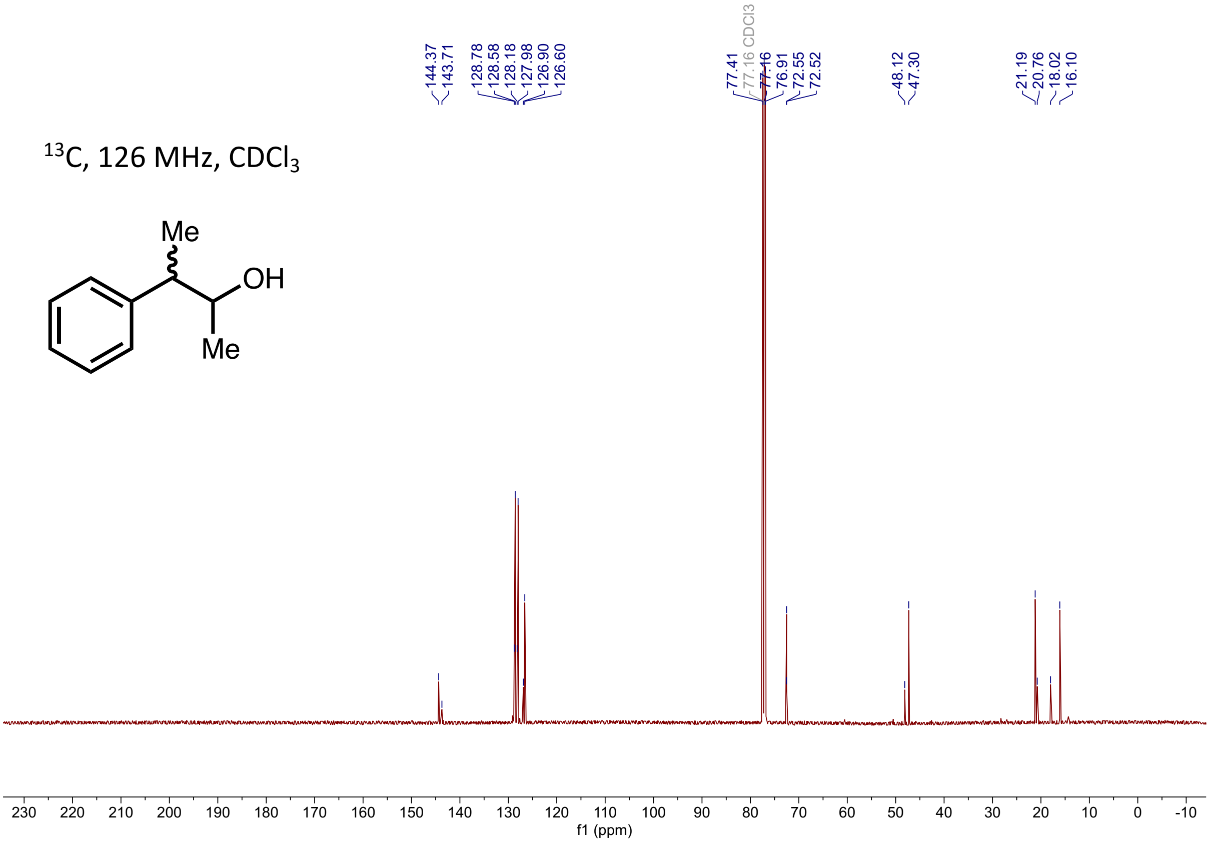
144.37 143.71 128.78 128.58 128.18 127.98 126.90 126.60 77.41 77.16 CDCl3 77.16 76.91 72.55 72.52 48.12 47.30 21.19 20.76 18.02 16.10
13C, 126 MHz, CDCl3
Me
OH
Me
230 220 210 200 190 180 170 160 150 140 130 120 110 100 90 80 70 60 50 40 30 20 10 0 -10
f1 (ppm)

(S9)

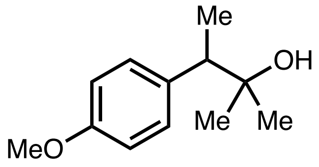

Prepared according to General Procedure A, step 3, using methyl 2-(4-methoxyphenyl)acetate (901 mg, 5 mmol), THF (10 mL), Methylmagnesium bromide (3.7 mL, 11 mmol, 2.2 equiv., 3M in Et_2O). The crude residue was purified by flash column chromatography (eluent = 5 to 10% EtOAc in hexanes, silica gel) to afford product as a pale-yellow oil (845 mg, 87% yield).

R_f = 0.28 (eluent = 20% EtOAc in hexanes); **ν_{max} / cm^{-1}** (thin film) 3437, 2968, 1512, 1463, 1365, 1240, 1176, 1033, 835; **^{1}H NMR (500 MHz, $CDCl_3$)** δ 7.28 – 7.26 (m, 2H), 6.96 – 6.94 (m, 2H), 3.88 (s, *J* = 3.8 Hz, 3H), 2.88 – 2.83 (m, 1H), 1.42 – 1.40 (m, 3H), 1.27 – 1.26 (m, 6H); **^{13}C NMR (126 MHz, $CDCl_3$)** δ 158.3, 135.5, 129.9, 113.5, 72.7, 55.3, 49.6, 28.0, 26.9, 16.0; **HRMS (EI$^+$)** [$C_{12}H_{18}O_2$] requires [M-CH_3]$^+$ 179.1077, found 179.1063 (- 2.20 ppm).

Data consistent with the literature.[4]

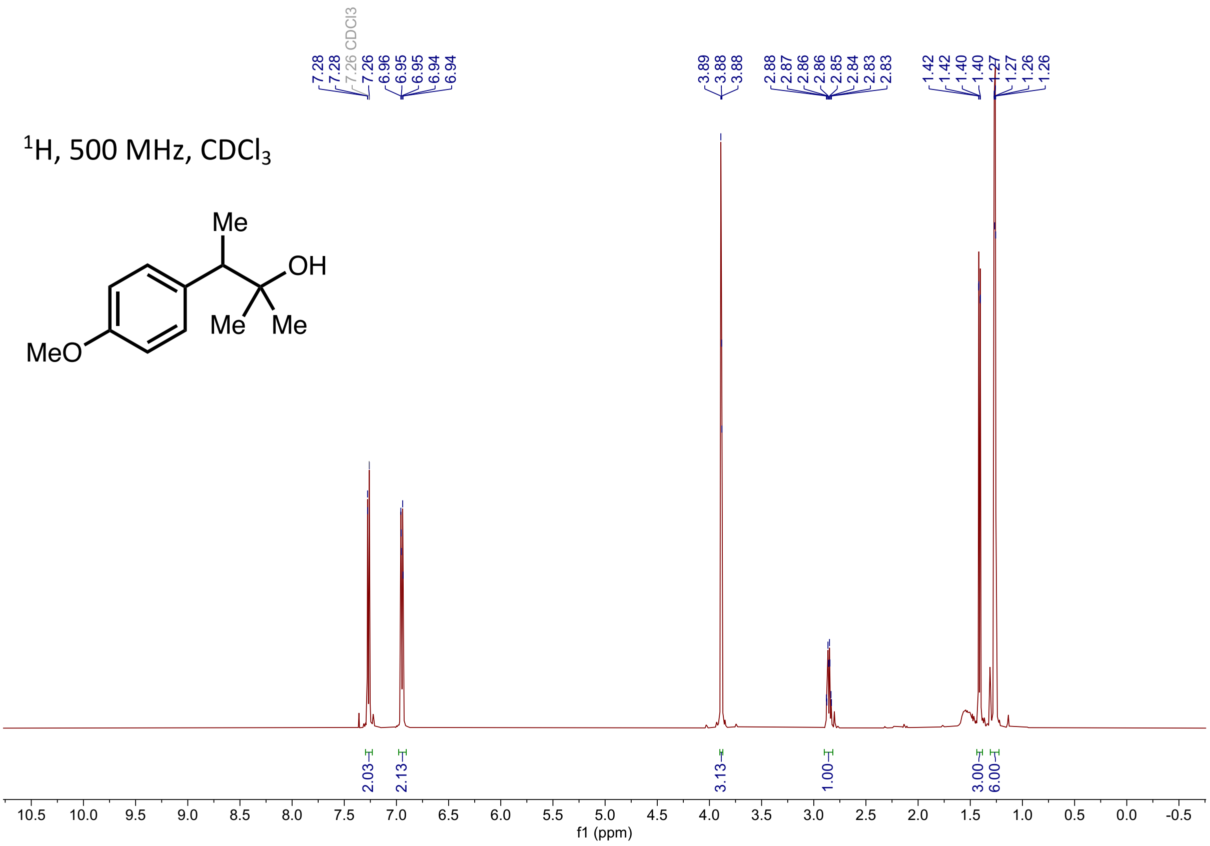
7.28
7.28
7.26 CDCl3
7.26
6.96
6.95
6.95
6.94
6.94
3.89
3.88
3.88
2.88
2.87
2.86
2.86
2.85
2.84
2.83
2.83
1.42
1.42
1.40
1.40
1.27
1.27
1.26
1.26
1H, 500 MHz, CDCl3
Me
OH
Me
Me
MeO
2.03
2.13
3.13
1.00
3.00
6.00
10.5
10.0
9.5
9.0
8.5
8.0
7.5
7.0
6.5
6.0
5.5
5.0
4.5
4.0
3.5
3.0
2.5
2.0
1.5
1.0
0.5
0.0
-0.5
f1 (ppm)

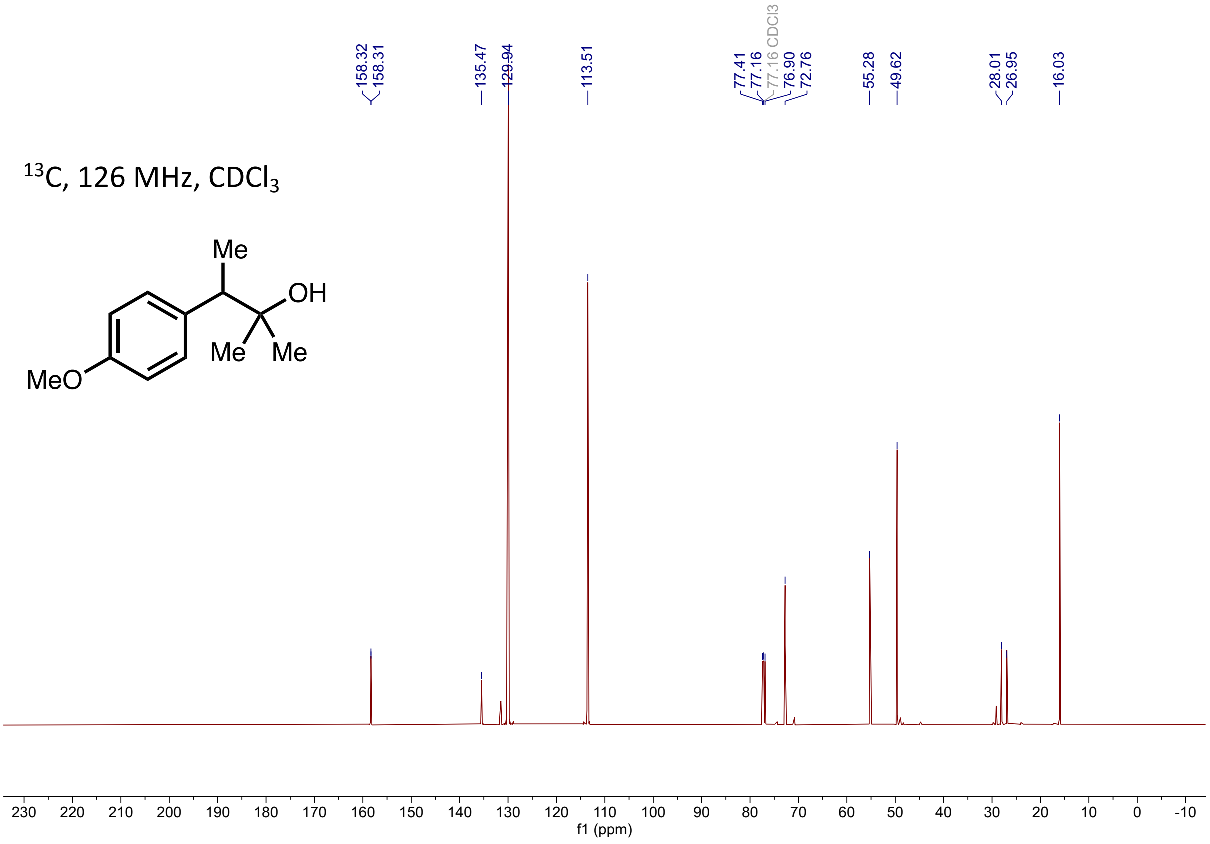
158.32
158.31
135.47
129.94
113.51
77.41
77.16
77.16 CDCl3
76.90
72.76
55.28
49.62
28.01
26.95
16.03
13C, 126 MHz, CDCl3
Me
OH
Me
Me
MeO
230
220
210
200
190
180
170
160
150
140
130
120
110
100
90
80
70
60
50
40
30
20
10
0
-10
f1 (ppm)

(S10)

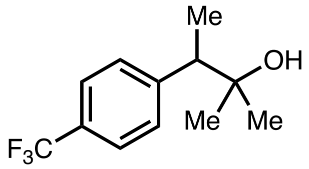

Prepared according to General Procedure A, step 3, using methyl 2-(4-(trifluoromethyl)phenyl)propanoate (1.16 g, 5 mmol), THF (10 mL), Methylmagnesium bromide (3.7 mL, 11 mmol, 2.2 equiv., 3M in Et_2O). The crude residue was purified by flash column chromatography (eluent = 5 to 10% EtOAc in hexanes, silica gel) to afford product as an orange oil (906 mg, 78% yield).

R_f = 0.24 (eluent = 20% EtOAc in hexanes); **ν_{max} / cm^{-1}** (thin film) 3420, 2978, 1462, 1422, 1373, 1320, 1163, 1116, 1066, 840; **^{1}H NMR (500 MHz, $CDCl_3$)** δ 7.43 – 7.42 (d, *J* = 8.1 Hz, 2H), 7.26 – 7.24 (d, *J* = 8.1 Hz, 2H), 2.75 – 2.70 (m, 1H), 1.23 – 1.22 (m, 3H), 1.07 (s, 2H), 1.03 (s, 2H); **^{13}C NMR (126 MHz, $CDCl_3$)** δ 147.9, 129.4, 128.9 (d, *J* = 1.4 Hz), 128.7 – 128.6 (m), 124.9 (tt, *J* = 3.8, 1.9 Hz), 72.6, 50.3, 28.3, 27.4, 15.8; **^{19}F NMR (471 MHz, $CDCl_3$)** δ -62.37; **HRMS (EI$^+$)** [$C_{12}H_{15}F_3O$] requires $[M-H]^+$ 231.0097, found 231.0090 (- 0.75 ppm).

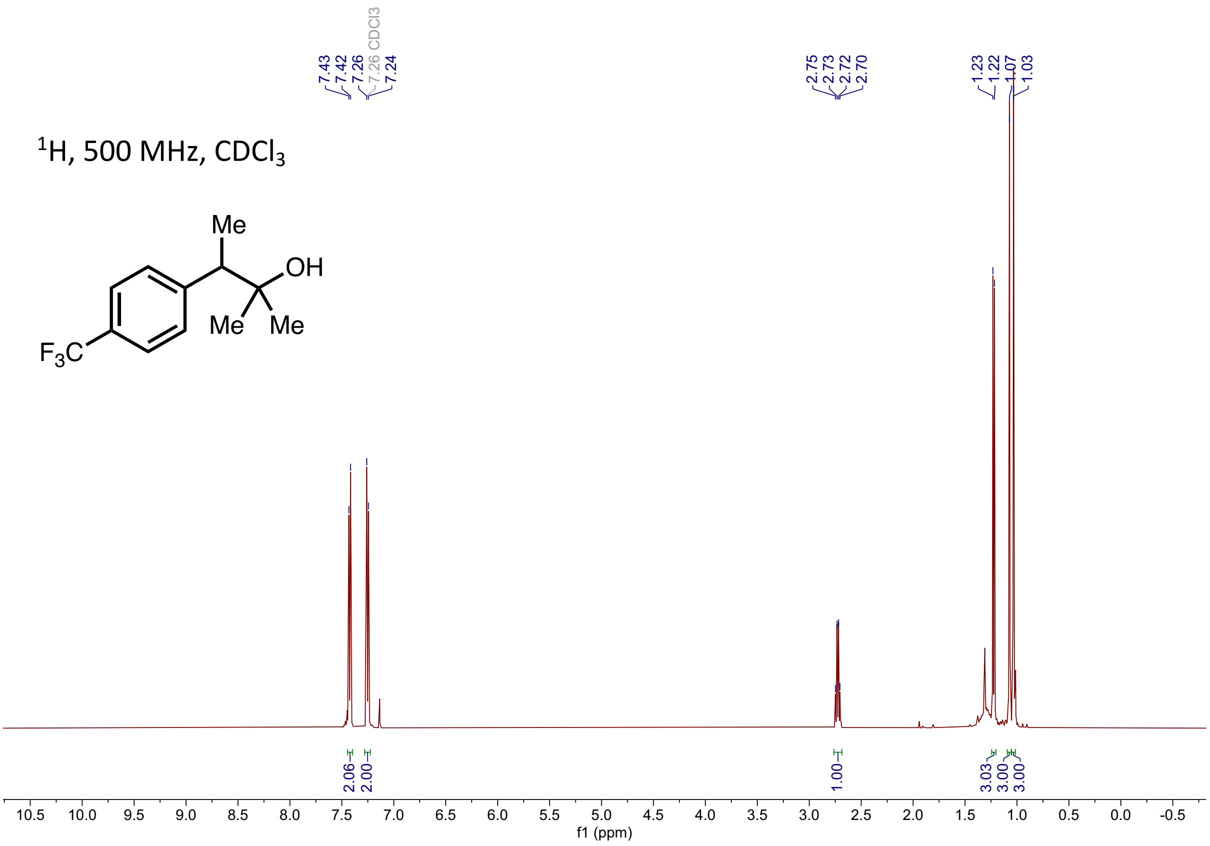

1H, 500 MHz, CDCl3
Me
OH
Me Me
F3C
7.43
7.42
7.26
7.26 CDCl3
7.24
2.75
2.73
2.72
2.70
1.23
1.22
1.07
1.03
2.06
2.00
1.00
3.03
3.00
3.00
10.5 10.0 9.5 9.0 8.5 8.0 7.5 7.0 6.5 6.0 5.5 5.0 4.5 4.0 3.5 3.0 2.5 2.0 1.5 1.0 0.5 0.0 -0.5
f1 (ppm)

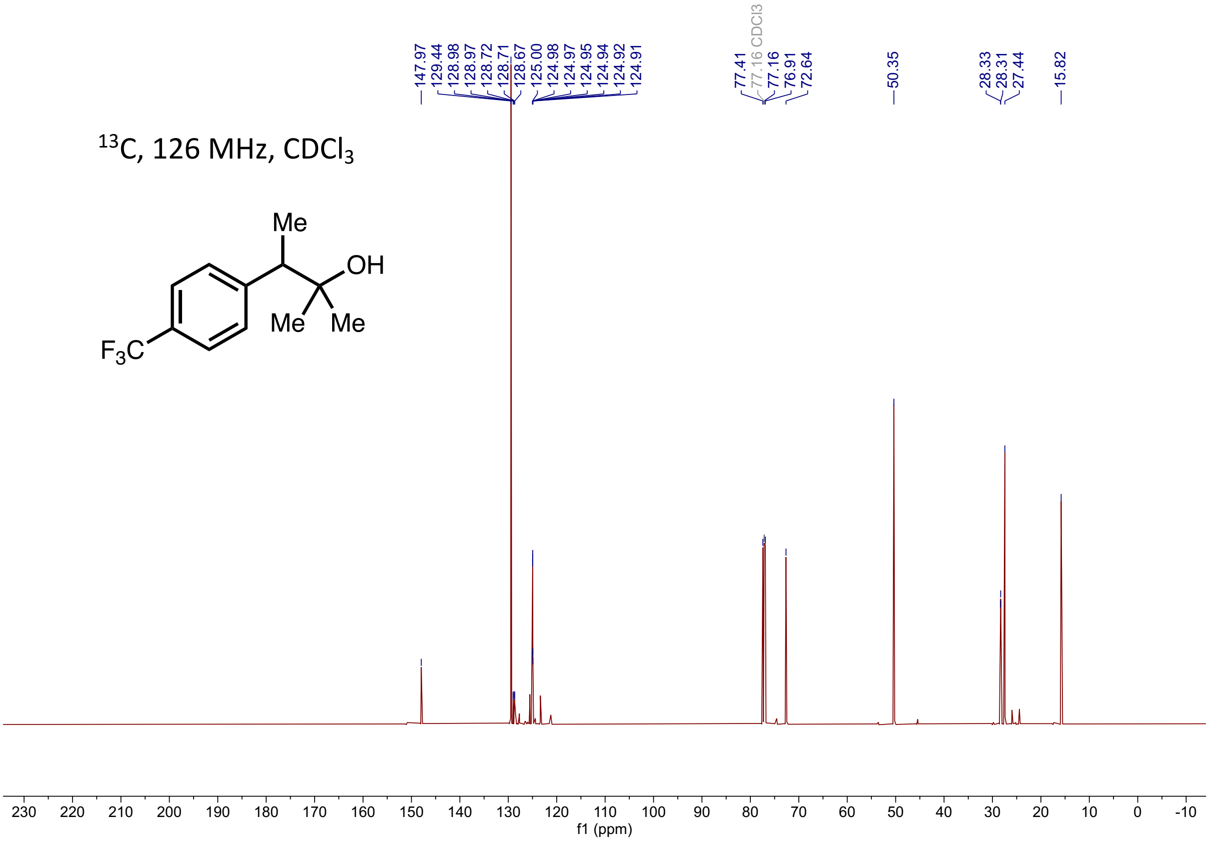

13C, 126 MHz, CDCl3
Me
OH
Me Me
F3C
147.97
129.44
128.98
128.97
128.72
128.71
128.67
125.00
124.98
124.97
124.95
124.94
124.92
124.91
77.41
77.16 CDCl3
77.16
76.91
72.64
50.35
28.33
28.31
27.44
15.82
230 220 210 200 190 180 170 160 150 140 130 120 110 100 90 80 70 60 50 40 30 20 10 0 -10
f1 (ppm)

^{19}F, 471 MHz, $CDCl_3$

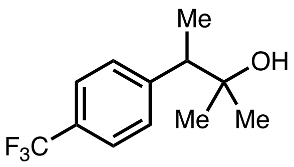

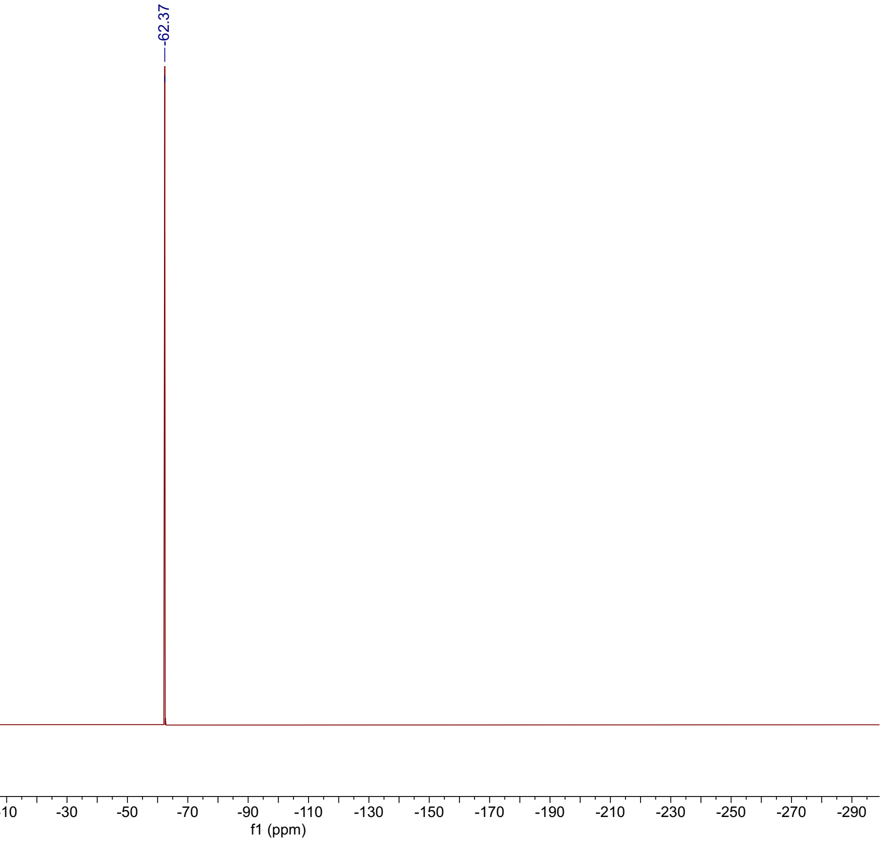

(S11)

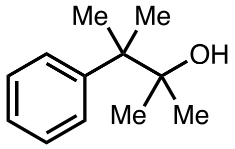

Prepared according to General Procedure A, step 3, using methyl 2-methyl-2-phenylpropanoate (891 mg, 5 mmol), THF (10 mL), methylmagnesium bromide (3.7 mL, 11 mmol, 2.2 equiv., 3M in Et_2O). The crude residue was purified by flash column chromatography (eluent = 5 to 10% EtOAc in hexanes, silica gel) to afford product as a pale-yellow oil (597 mg, 67% yield).

R_f = 0.61 (eluent = 20% EtOAc in hexanes); **ν_{max} / cm^{-1}** (thin film) 3468, 2976, 1442, 1373, 702; **^{1}H NMR (500 MHz, $CDCl_3$)** δ 7.48 – 7.45 (m, 2H), 7.35 – 7.31 (m, 2H), 7.25 – 7.21 (m, 1H), 1.44 (s, 6H), 1.31 (br, 1H), 1.15 (s, 6H); **^{13}C NMR (126 MHz, $CDCl_3$)** δ 146.3, 128.2, 127.7, 126.1, 74.6, 45.2, 25.7, 24.5; **HRMS (EI$^+$)** [$C_{12}H_{18}O$] requires [M-CH_3]$^+$ 163.1128, found 163.1115 (-1.41 ppm).

Data consistent with the literature.[4]

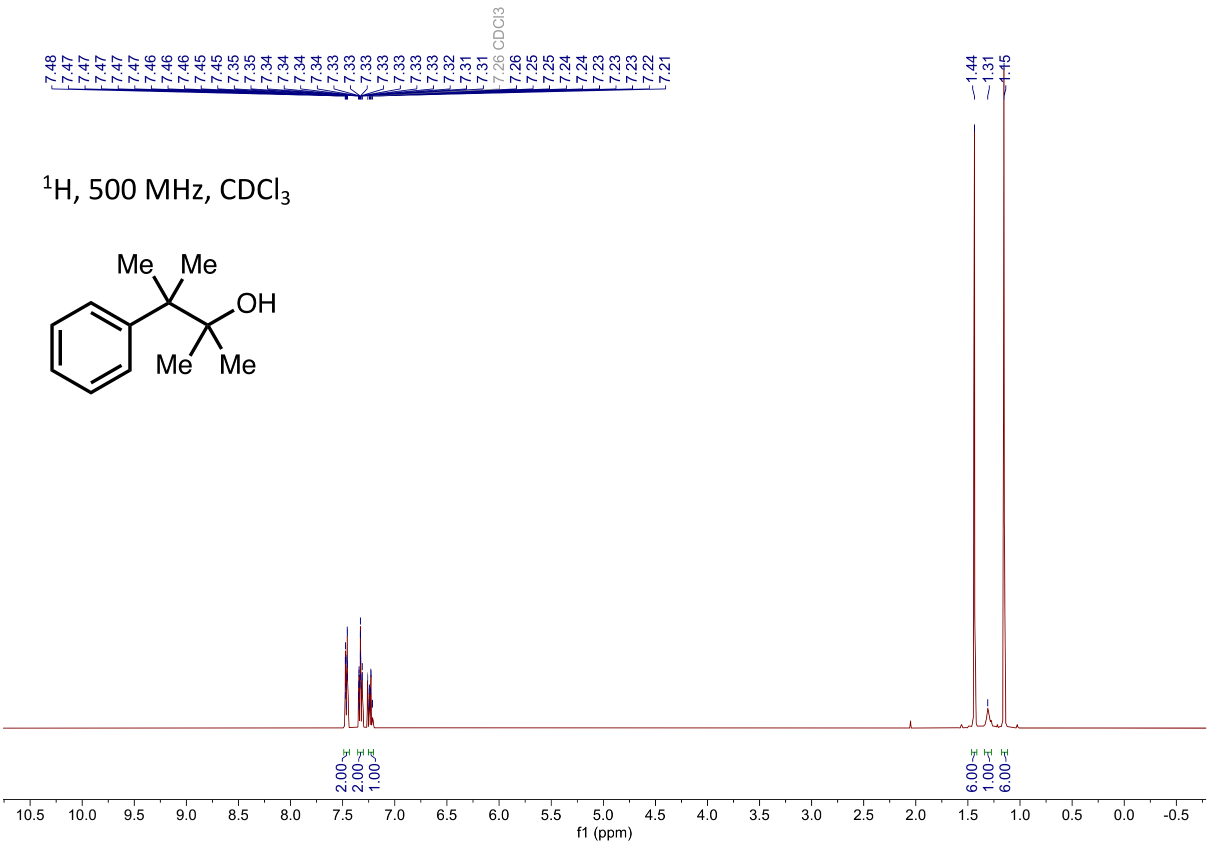
7.48
7.47
7.46
7.45
7.35
7.34
7.33
7.32
7.31
7.26 CDCl3
7.25
7.24
7.23
7.22
7.21
1.44
1.31
1.15
1H, 500 MHz, CDCl3
Me
Me
OH
Me
Me
2.00
2.00
1.00
6.00
1.00
6.00
10.5
10.0
9.5
9.0
8.5
8.0
7.5
7.0
6.5
6.0
5.5
5.0
4.5
4.0
3.5
3.0
2.5
2.0
1.5
1.0
0.5
0.0
-0.5
f1 (ppm)

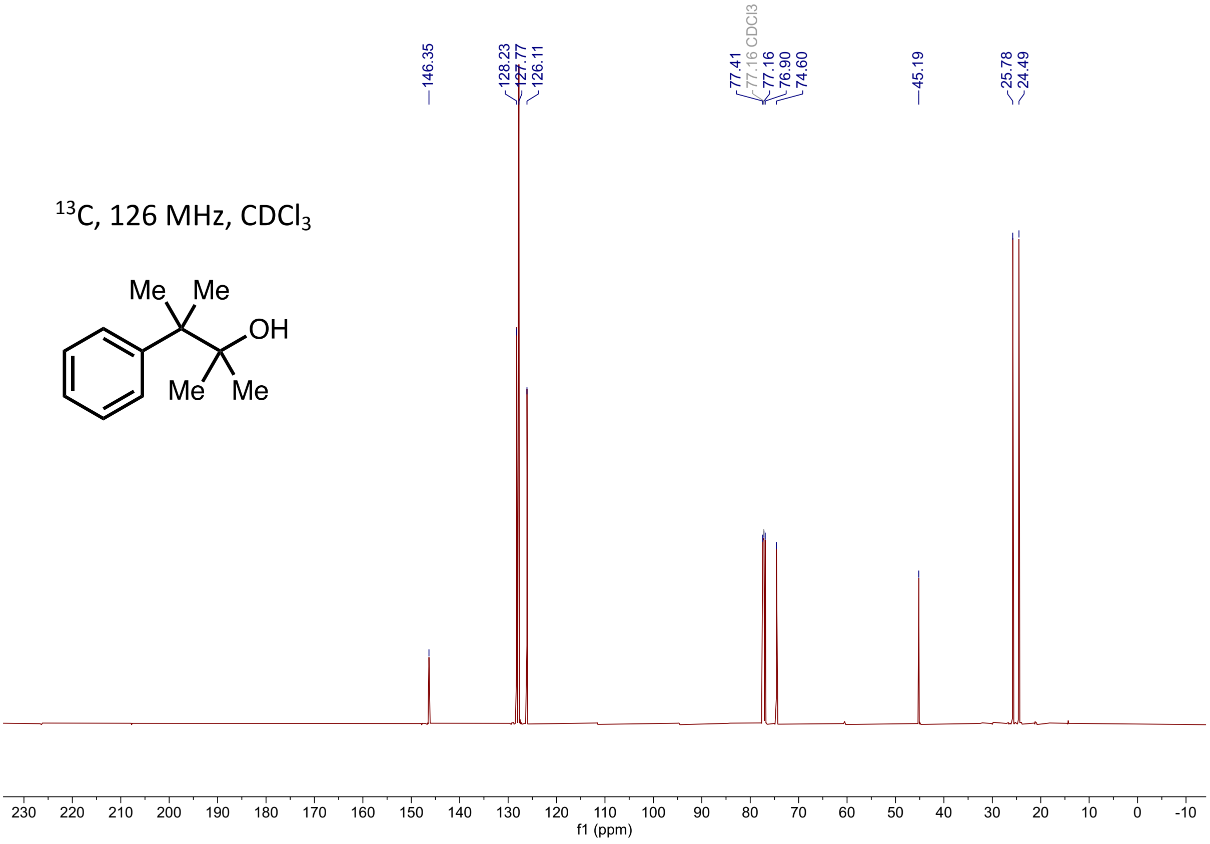
146.35
128.23
127.77
126.11
77.41
77.16 CDCl3
77.16
76.90
74.60
45.19
25.78
24.49
13C, 126 MHz, CDCl3
Me
Me
OH
Me
Me
230
220
210
200
190
180
170
160
150
140
130
120
110
100
90
80
70
60
50
40
30
20
10
0
-10
f1 (ppm)

(S12)

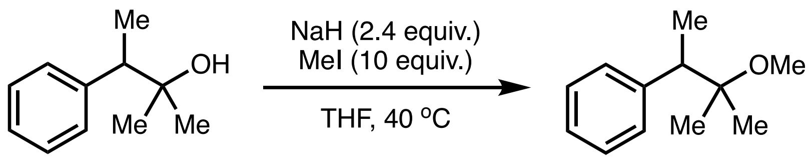

To a solution of **1** (493 mg, 3 mmol, 1 equiv.) in THF (15 mL) at 0 °C was added sodium hydride (288 mg, 7.2 mmol, 2.4 equiv., 60 % in paraffin oil). The mixture was stirred at 0 °C for 15 minutes before methyl iodide (4.26 g, 30 mmol, 10 equiv.) was added and the mixture was warmed to room temperature, stirred for 3 h and then heated to 40 °C overnight. The mixture was then quenched with a saturated solution of ammonium chloride (7.5 mL) and extracted with EtOAc (3 x 15 mL). The organics were combined, dried over $MgSO_4$, filtered and concentrated *in vacuo* affording crude product. The crude residue was purified by flash column chromatography (eluent = 10 to 20% EtOAc in hexanes, silica gel) to afford product as a colourless oil (246 mg, 46% yield).

R_f = 0.41 (eluent = 10% EtOAc in hexanes); **ν_{max} / cm^{-1}** (thin film) 2970, 2931, 1454, 1365, 1186, 1145, 1076, 769, 696; **^{1}H NMR (500 MHz, $CDCl_3$)** δ 7.28 – 7.18 (m, 5H), 3.23 (s, 3H), 2.94 – 2.90 (q, *J* = 7.2 Hz, 1H), 1.31 – 1.29 (d, *J* = 7.2 Hz, 3H), 1.08 (s, 3H), 1.06 (s, 3H); **^{13}C NMR (126 MHz, $CDCl_3$)** δ 144.2, 129.3, 127.8, 126.2, 76.9, 49.2, 48.0, 23.4, 22.1, 15.5; **HRMS (EI^+)** [$C_{12}H_{18}O$] requires $[M-CH_3]^+$ 163.1123, found 163.1117 (-1.41 ppm).

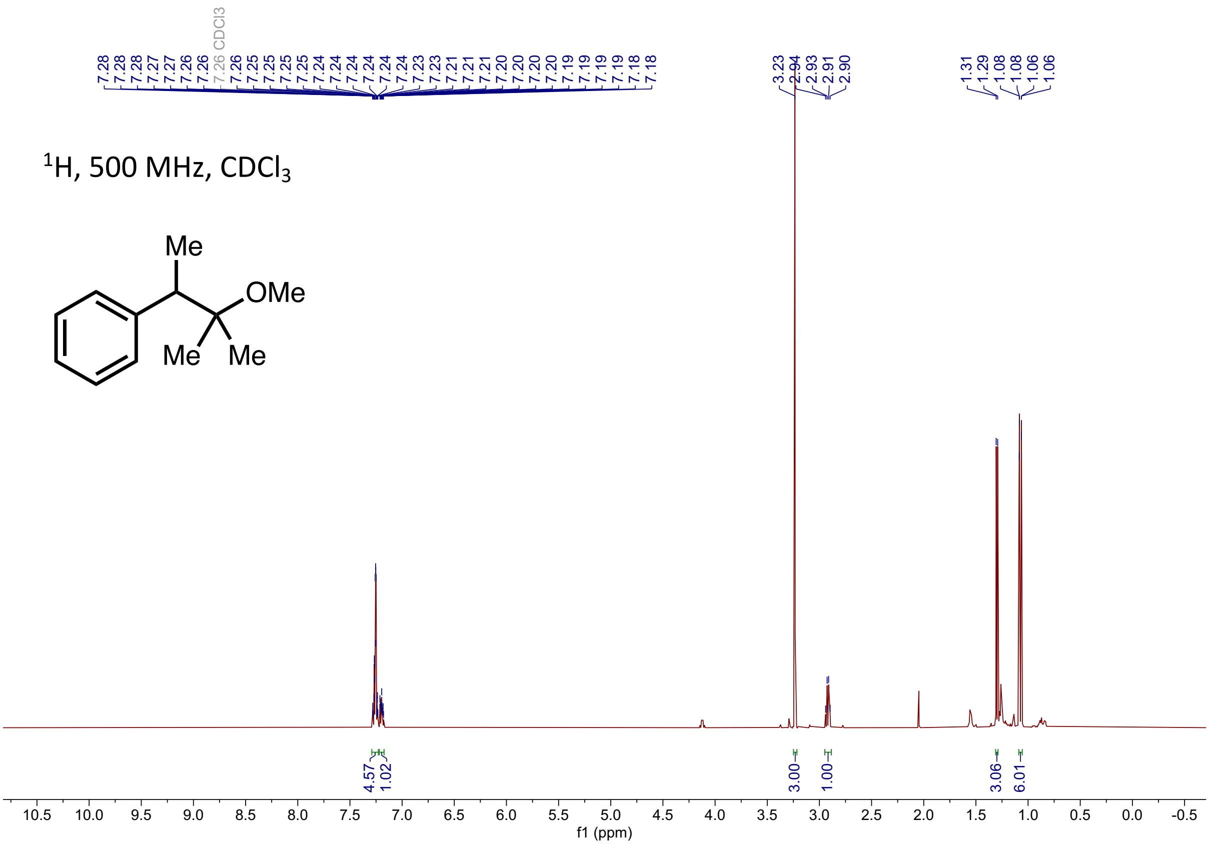

^{1}H, 500 MHz, CDCl$_3$
Me
OMe
Me
Me
7.28 7.28 7.28 7.27 7.26 7.26 7.26 CDCl3 7.26 7.25 7.25 7.25 7.25 7.24 7.24 7.24 7.24 7.24 7.24 7.23 7.23 7.21 7.21 7.21 7.20 7.20 7.20 7.20 7.20 7.19 7.19 7.19 7.19 7.18 7.18
3.23 2.94 2.93 2.91 2.90
1.31 1.29 1.08 1.08 1.06 1.06
4.57 1.02 3.00 1.00 3.06 6.01
f1 (ppm)

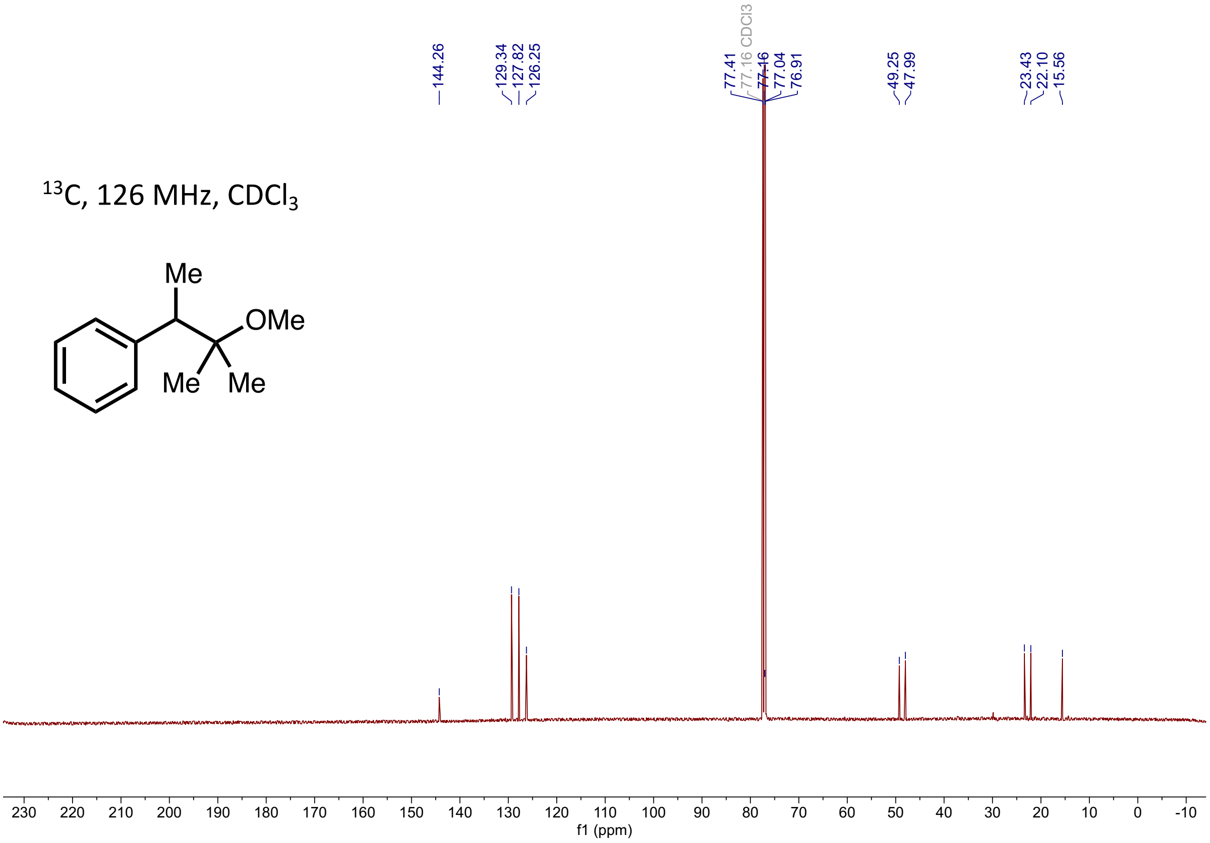

^{13}C, 126 MHz, CDCl$_3$
Me
OMe
Me
Me
144.26
129.34 127.82 126.25
77.41 77.16 CDCl3 77.16 77.04 76.91
49.25 47.99
23.43 22.10 15.56
f1 (ppm)

(S13)

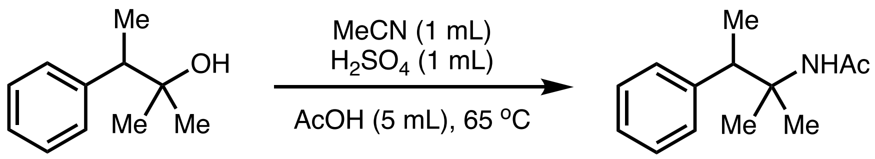

To a solution of **1** (600 mg, 3.65 mmol, 1 equiv.) in AcOH (5 mL) at room temperature was added MeCN (1 mL), followed concentrated H_2SO_4 (1 mL) dropwise. The mixture was heated to 65 °C and stirred for 3 h. After that, the mixture was poured into ice-water (200 mL) and the aqueous mixture was basified with addition of saturated aqueous NaOH solution until pH>11. The resulted suspension was stirred for further 0.5 h, and then the precipitate was filtered and washed with water. The resulted residue was concentrated *in vacuo* affording crude product. The crude residue was purified by flash column chromatography (eluent = 5 to 10% EtOAc in hexanes, silica gel) to afford product as a yellow oil (532 mg, 71% yield).

R_f = 0.15 (eluent = 25% EtOAc in hexanes); **v_{max} / cm^{-1}** (thin film) 3292, 2974, 1651, 1546, 1450, 1369, 1269, 740, 700; **^{1}H NMR (500 MHz, $CDCl_3$)** δ 7.34 – 7.24 (m, 5H), 5.10 (s, 1H), 3.73 – 3.68 (q, *J* = 7.3 Hz, 1H), 1.91 (s, 3H), 1.45 (s, 3H), 1.33 – 1.32 (d, *J* = 7.3 Hz, 3H), 1.22 (s, 3H); **^{13}C NMR (126 MHz, $CDCl_3$)** δ 169.8, 143.4, 129.2, 127.9, 126.5, 57.0, 44.9, 25.2, 24.7, 24.1, 15.6; **HRMS (ES$^+$)** [$C_{13}H_{19}NO$] requires $[M+H]^+$ 206.1545, found 206.1537 (-3.9 ppm).

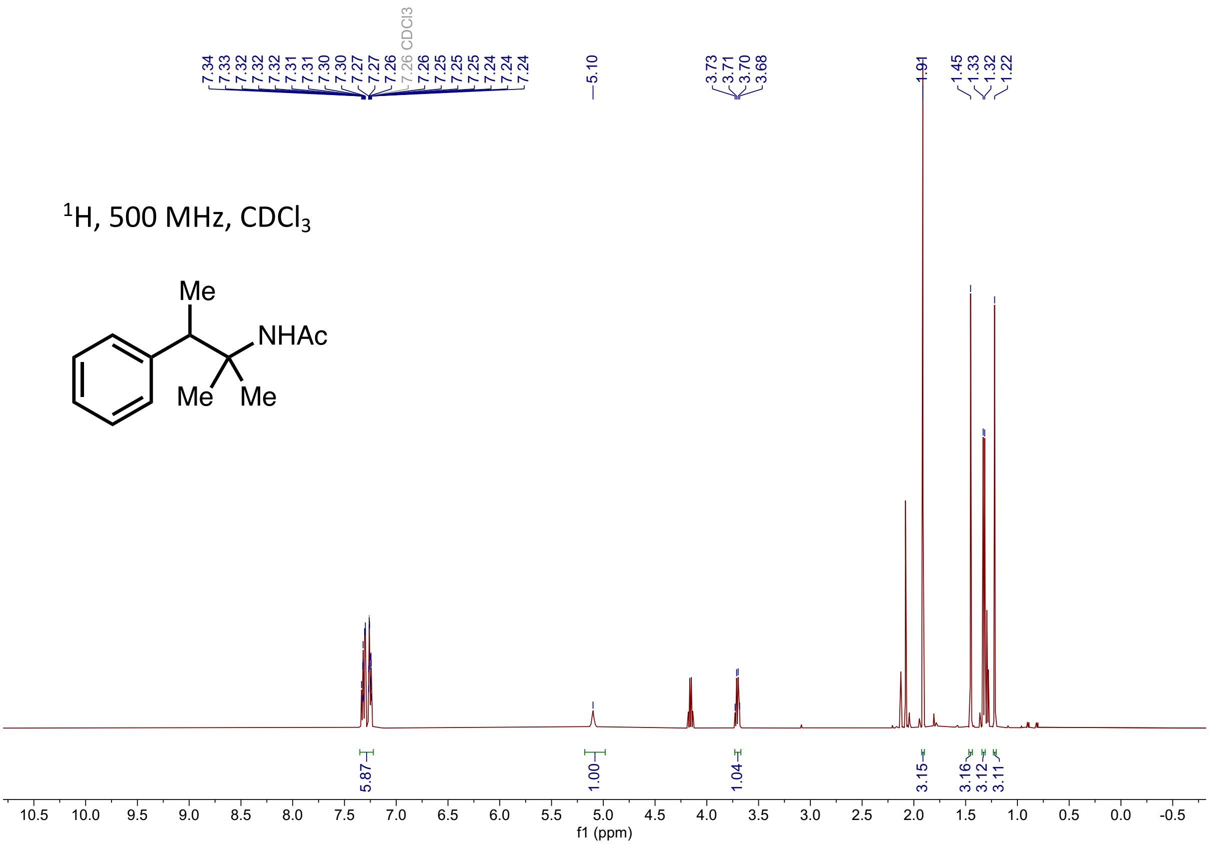

7.34 7.33 7.32 7.32 7.32 7.31 7.31 7.30 7.30 7.27 7.27 7.26 7.26 CDCl3 7.26 7.25 7.25 7.25 7.25 7.24 7.24 7.24
5.10
3.73 3.71 3.70 3.68
1.91
1.45 1.33 1.32 1.22
^{1}H, 500 MHz, CDCl$_3$
Me
NHAc
Me Me
5.87
1.00
1.04
3.15
3.16 3.12 3.11
f1 (ppm)

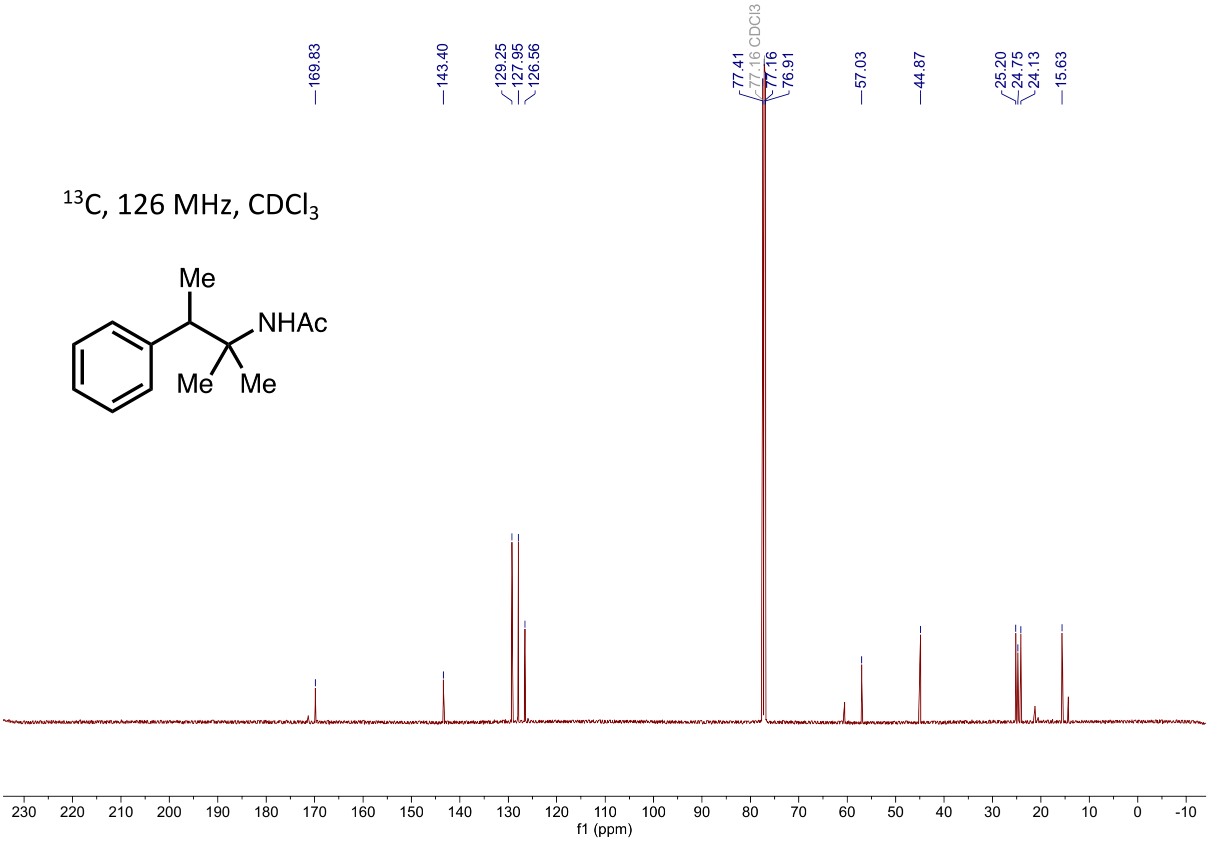

169.83
143.40
129.25 127.95 126.56
77.41 77.16 CDCl3 77.16 76.91
57.03
44.87
25.20 24.75 24.13
15.63
^{13}C, 126 MHz, CDCl$_3$
Me
NHAc
Me Me
f1 (ppm)

(S16)

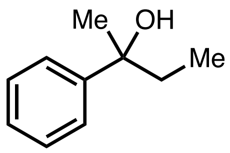

Prepared according to General Procedure C, using propiophenone (671 mg, 5 mmol), THF (10 mL), Methylmagnesium bromide (2 mL, 6 mmol, 1.2 equiv., 3M in Et_2O). The crude residue was purified by flash column chromatography (eluent = 5 to 10% EtOAc in hexanes, silica gel) to afford product as a colourless oil (684 mg, 91% yield).

R_f = 0.28 (eluent = 20% EtOAc in hexanes); **^{1}H NMR (500 MHz, $CDCl_3$)** δ 7.45 – 7.42 (m, 2H), 7.36 – 7.32 (m, 2H), 7.26 – 7.22 (m, 1H), 1.90 – 1.78 (m, 2H), 1.55 (s, 3H), 0.82 – 0.79 (t, *J* = 7.5 Hz, 3H); **^{13}C NMR (126 MHz, $CDCl_3$)** δ 147.8, 128.2, 126.6, 125.0, 75.0, 36.8, 29.7, 8.4.

Data consistent with the literature.[5]

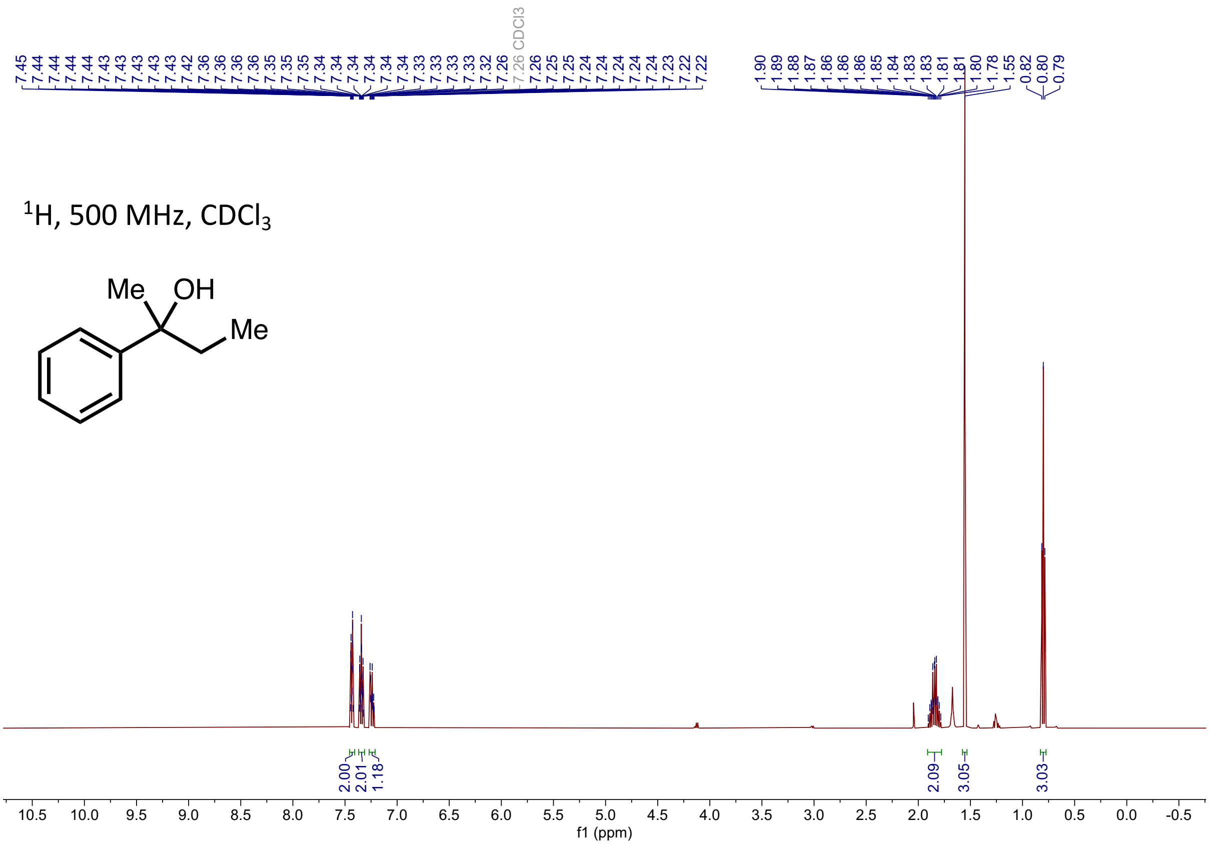
7.45 7.44 7.44 7.44 7.44 7.43 7.43 7.43 7.43 7.42 7.36 7.36 7.36 7.36 7.35 7.35 7.34 7.34 7.34 7.34 7.34 7.33 7.33 7.33 7.33 7.32 7.26 CDCl3 7.26 7.25 7.25 7.24 7.24 7.24 7.24 7.24 7.23 7.22 7.22
1.90 1.89 1.88 1.87 1.86 1.86 1.86 1.85 1.84 1.83 1.83 1.81 1.81 1.80 1.78 1.55 0.82 0.80 0.79
¹H, 500 MHz, CDCl3
Me
OH
Me
2.00 2.01 1.18
2.09 3.05 3.03
f1 (ppm)

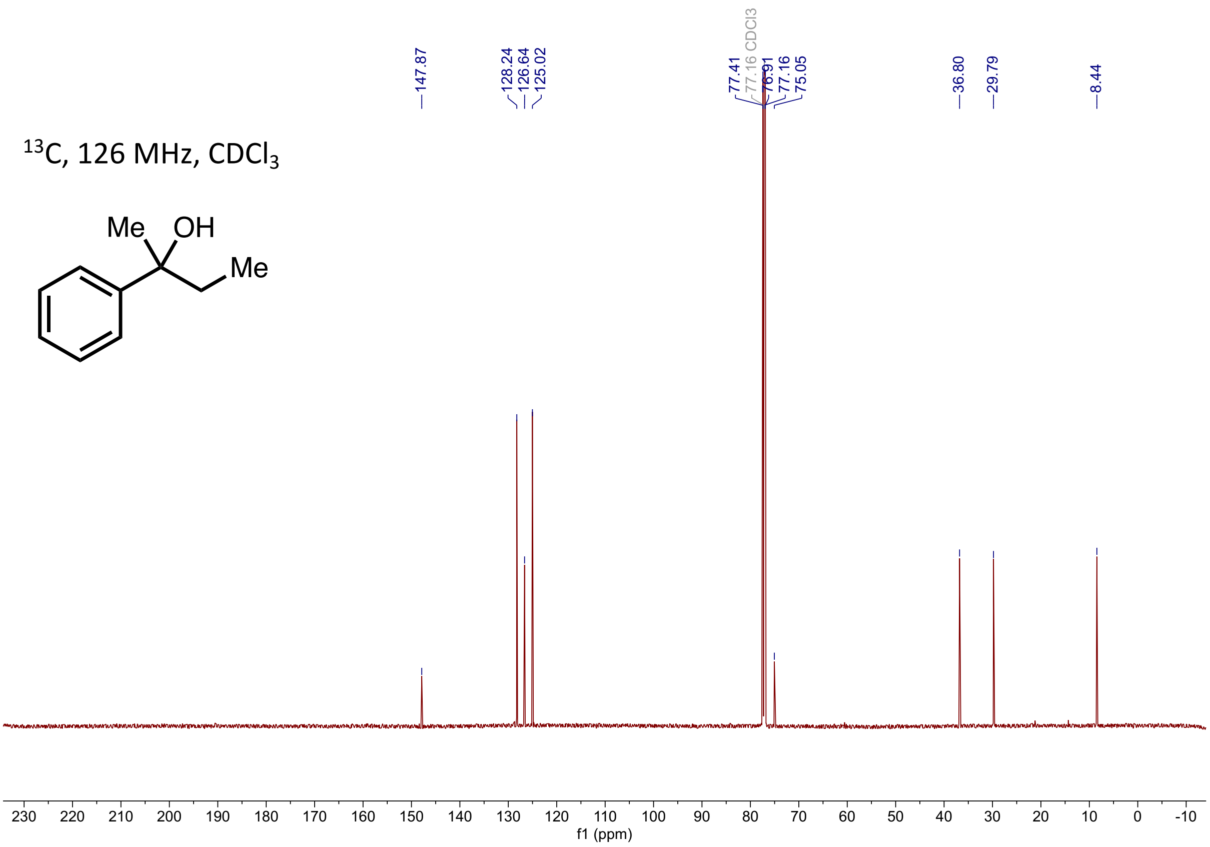
147.87
128.24 126.64 125.02
77.41 77.16 CDCl3 76.91 77.16 75.05
36.80 29.79 8.44
¹³C, 126 MHz, CDCl3
Me
OH
Me
f1 (ppm)

(S17)

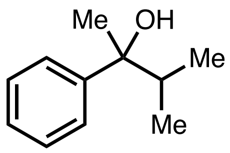

Prepared according to General Procedure C, using 2-methyl-1-phenylpropan-1-one (741 mg, 5 mmol), THF (10 mL), Methylmagnesium bromide (2 mL, 6 mmol, 1.2 equiv., 3M in Et_2O). The crude residue was purified by flash column chromatography (eluent = 5 to 10% EtOAc in hexanes, silica gel) to afford product as a yellow oil (706 mg, 86% yield).

R_f = 0.31 (eluent = 20% EtOAc in hexanes); **^{1}H NMR (300 MHz, $CDCl_3$)** δ 7.44 – 7.40 (m, 2H), 7.36 – 7.30 (m, 2H), 7.26 – 7.20 (m, 1H), 2.07 – 1.98 (p, *J* = 6.9 Hz, 1H), 1.53 (s, 3H), 0.91 – 0.88 (d, *J* = 6.8 Hz, 3H), 0.82 – 0.80 (d, *J* = 6.8 Hz, 3H); **^{13}C NMR (75 MHz, $CDCl_3$)** δ 147.9, 126.5, 125.4, 76.9, 38.7, 26.8, 17.5, 17.3.

Data consistent with the literature.[6]

^{1}H, 300 MHz, $CDCl_3$

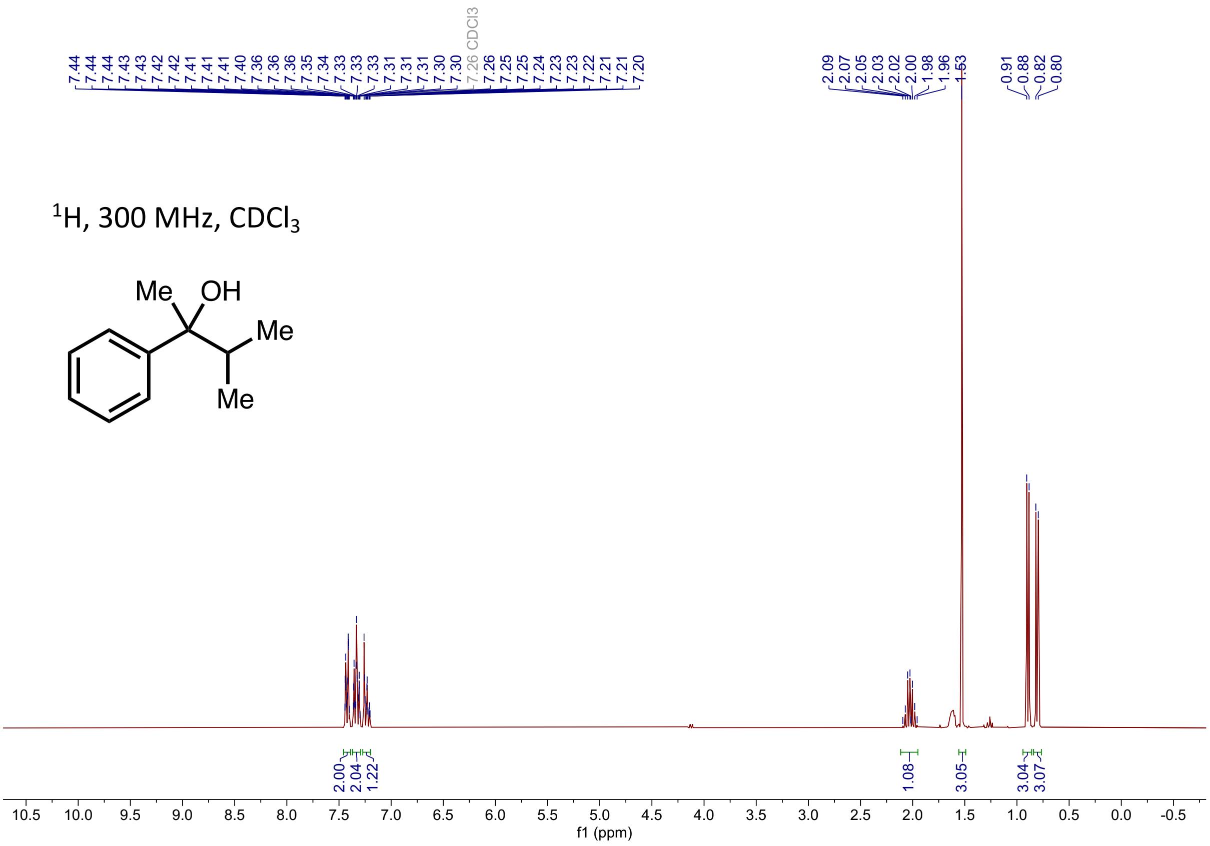

^{13}C, 75 MHz, $CDCl_3$

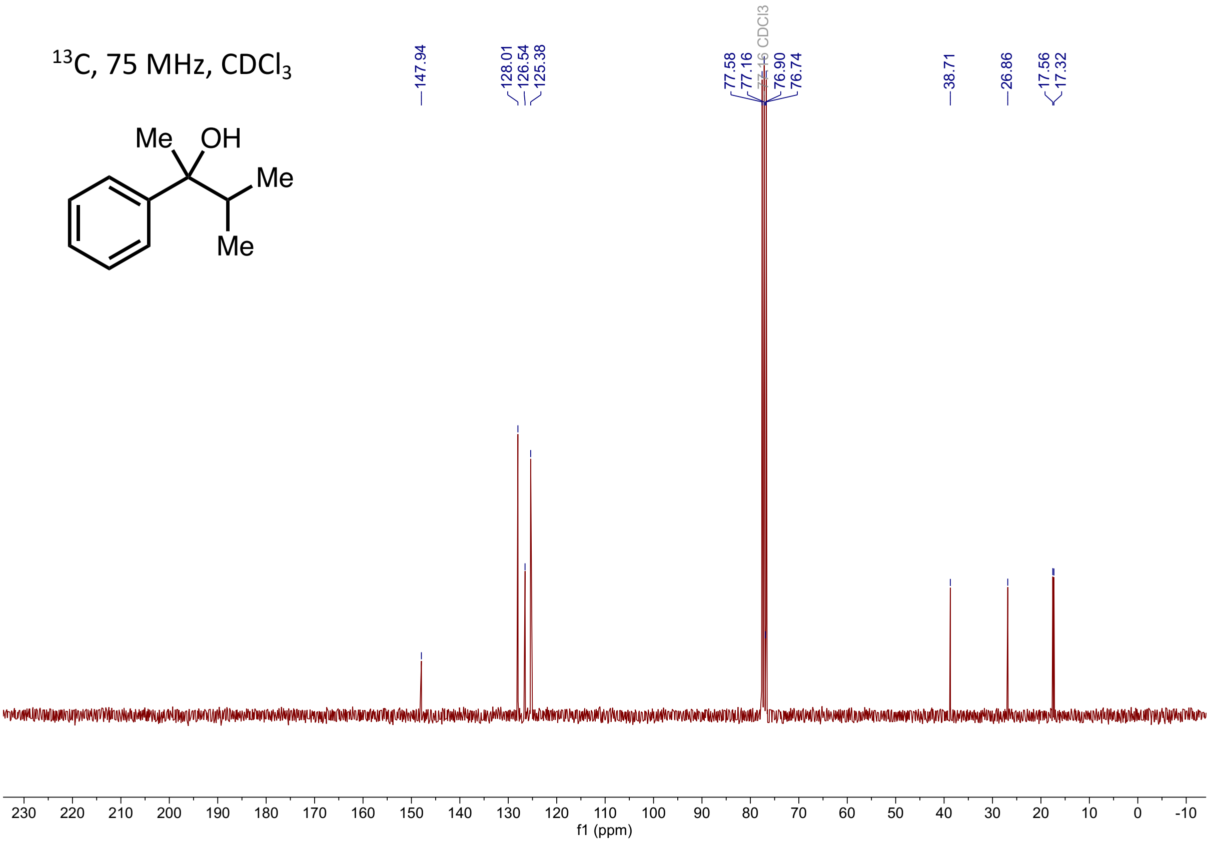

(S18)

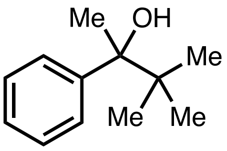

Prepared according to General Procedure B, using magnesium turnings (219 mg, 9 mmol), iodine (1 crystal), bromobenzene (0.80 mL, 7.5 mmol), and pinacolone (501 mg, 5 mmol). The crude residue was purified by flash column chromatography (eluent = 5 to 10% EtOAc in hexanes, silica gel) to afford product as pale-yellow oil (419 mg, 47% yield).

R_f = 0.33 (eluent = 20% EtOAc in hexanes); **^{1}H NMR (500 MHz, $CDCl_3$)** δ 7.47 – 7.44 (m, 2H), 7.33 – 7.29 (m, 2H), 7.26 – 7.22 (m, 1H), 1.61 (s, 3H), 0.94 (9, 3H); **^{13}C NMR (126 MHz, $CDCl_3$)** δ 146.3, 127.2, 127.2, 126.4, 78.7, 38.0, 25.8, 25.3.

Data consistent with the literature.[2]

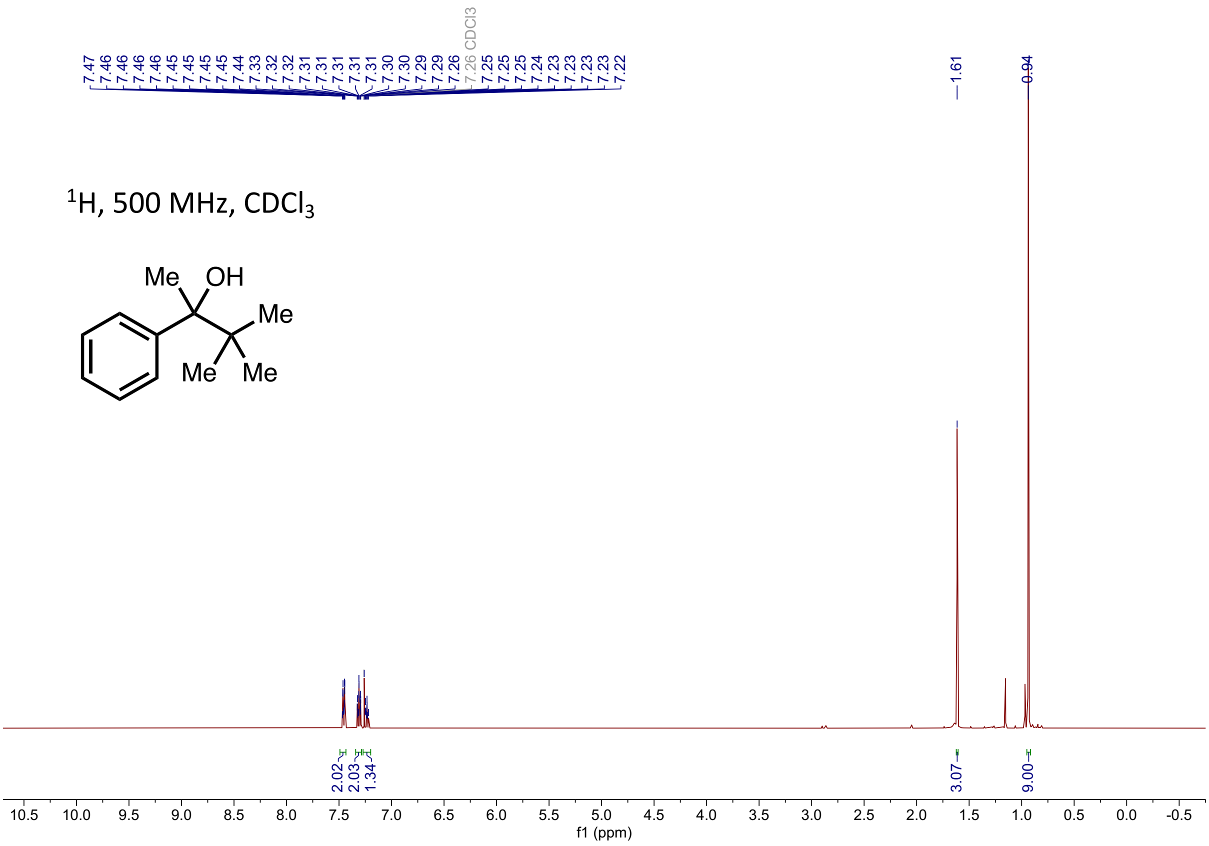
^1H, 500 MHz, $CDCl_3$
Me OH Me Me Me
7.47 7.46 7.46 7.46 7.45 7.45 7.45 7.45 7.44 7.33 7.32 7.32 7.31 7.31 7.31 7.31 7.31 7.30 7.30 7.29 7.29 7.26 7.26 CDCl3 7.25 7.25 7.25 7.24 7.23 7.23 7.23 7.23 7.23 7.22
1.61
0.94
2.02 2.03 1.34 3.07 9.00
10.5 10.0 9.5 9.0 8.5 8.0 7.5 7.0 6.5 6.0 5.5 5.0 4.5 4.0 3.5 3.0 2.5 2.0 1.5 1.0 0.5 0.0 -0.5
f1 (ppm)

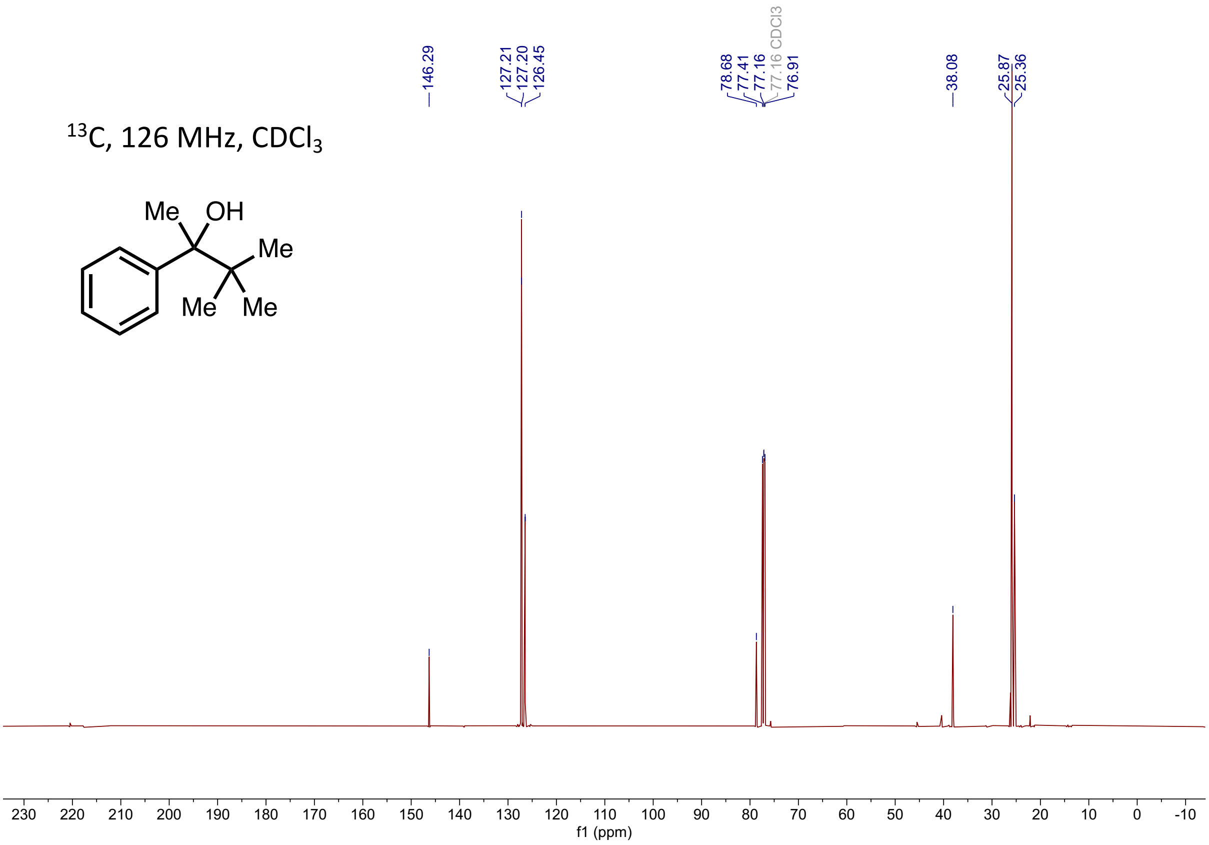
^{13}C, 126 MHz, $CDCl_3$
Me OH Me Me Me
146.29
127.21 127.20 126.45
78.68 77.41 77.16 77.16 CDCl3 76.91
38.08
25.87 25.36
230 220 210 200 190 180 170 160 150 140 130 120 110 100 90 80 70 60 50 40 30 20 10 0 -10
f1 (ppm)

(S19)

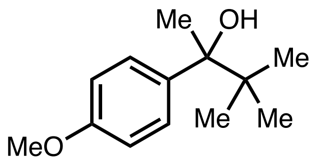

Prepared according to General Procedure B, using magnesium turnings (219 mg, 9 mmol), iodine (1 crystal), bromoanisole (0.94 mL, 7.5 mmol), and pinacolone (501 mg, 5 mmol). The crude residue was purified by flash column chromatography (eluent = 5 to 10% EtOAc in hexanes, silica gel) to afford product as colourless oil (917 mg, 88% yield).

R_f = 0.31 (eluent = 20% EtOAc in hexanes); **^{1}H NMR (500 MHz, $CDCl_3$)** δ 7.37 – 7.34 (m, 2H), 6.85 – 6.82 (m, 2H), 3.81 (s, 3H), 1.59 (s, 3H), 0.92 (s, 9H); **^{13}C NMR (126 MHz, $CDCl_3$)** δ 158.2, 138.5, 128.3, 112.5, 78.4, 55.3, 38.2, 25.9, 25.5.

Data consistent with the literature.[7]

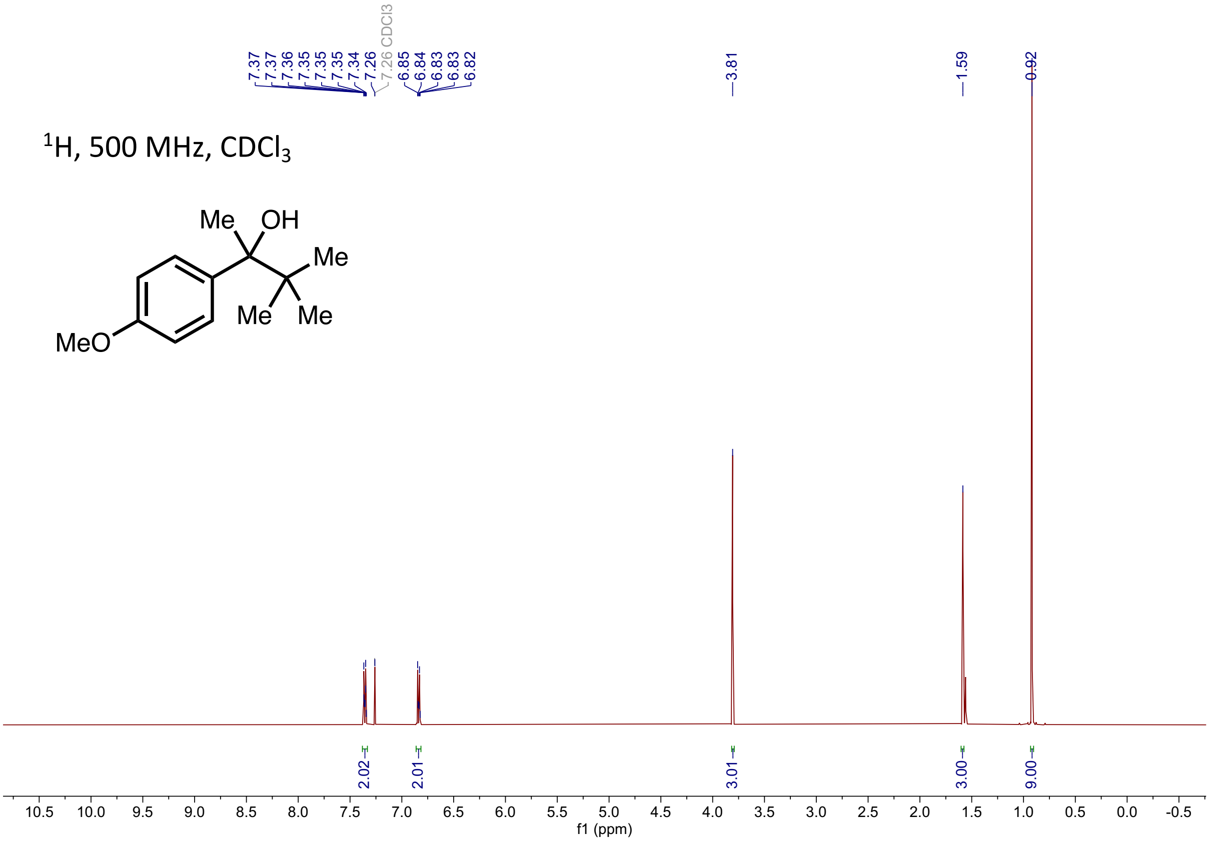

7.37
7.37
7.36
7.35
7.35
7.35
7.34
7.26
7.26 CDCl3
6.85
6.84
6.83
6.83
6.82
3.81
1.59
0.92
¹H, 500 MHz, CDCl₃
Me
OH
Me
Me
Me
MeO
2.02
2.01
3.01
3.00
9.00
10.5 10.0 9.5 9.0 8.5 8.0 7.5 7.0 6.5 6.0 5.5 5.0 4.5 4.0 3.5 3.0 2.5 2.0 1.5 1.0 0.5 0.0 -0.5
f1 (ppm)

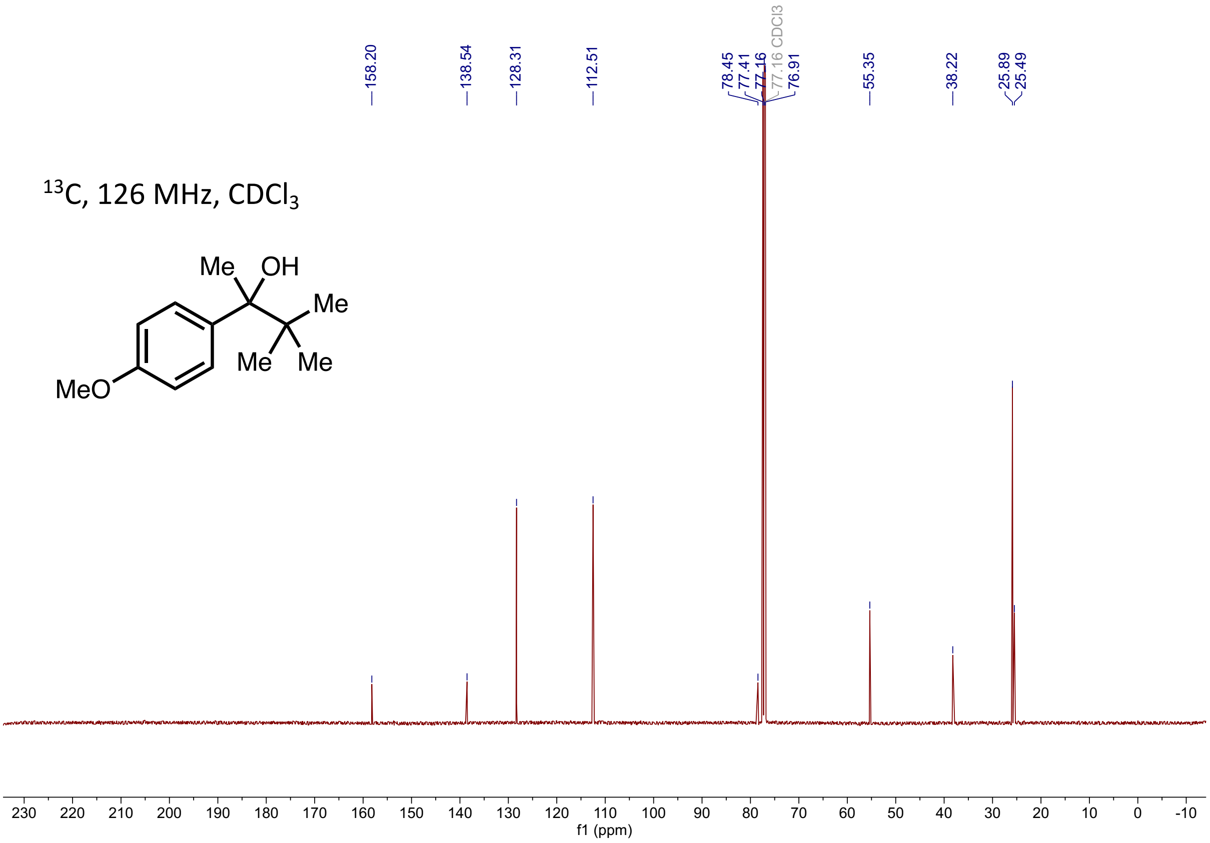

158.20
138.54
128.31
112.51
78.45
77.41
77.16
77.16 CDCl3
76.91
55.35
38.22
25.89
25.49
¹³C, 126 MHz, CDCl₃
Me
OH
Me
Me
Me
MeO
230 220 210 200 190 180 170 160 150 140 130 120 110 100 90 80 70 60 50 40 30 20 10 0 -10
f1 (ppm)

(S20)

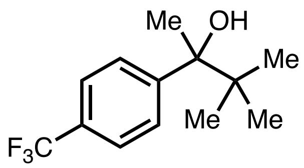

Prepared according to General Procedure B, using magnesium turnings (292 mg, 12 mmol), iodine (1 crystal), 4-bromobenzotrifluoride (2.25 g, 10 mmol), and pinacolone (501 mg, 5 mmol). The crude residue was purified by flash column chromatography (eluent = 5 to 10% EtOAc in hexanes, silica gel) to afford product as a colourless oil (665 mg, 54% yield).

R_f = 0.28 (eluent = 20% EtOAc in hexanes); **ν_{max} / cm^{-1}** (thin film) 3485, 2980, 1323, 1163, 1122, 1074, 840; **^{1}H NMR (500 MHz, $CDCl_3$)** δ 7.58 – 7.54 (m, 4H), 1.62 (s, 3H), 0.93 (s, 9H); **^{13}C NMR (126 MHz, $CDCl_3$)** δ 150.3, 129.1 (d, *J* = 6.3 Hz), 128.9, 128.6, 128.2 (d, *J* = 4.6 Hz), 127.0, 125.5, 124.7 (dt, *J* = 5.3, 3.1 Hz), 123.5 (dd, *J* = 8.6, 4.2 Hz), 78.6, 38.2, 26.3 – 24.2 (m); **^{19}F NMR (471 MHz, $CDCl_3$)** δ -62.36; **HRMS (EI$^+$)** [$C_{13}H_{17}F_3O$] requires $[M-CH_3]^+$ 231.1001, found 231.0986 (-2.07 ppm).

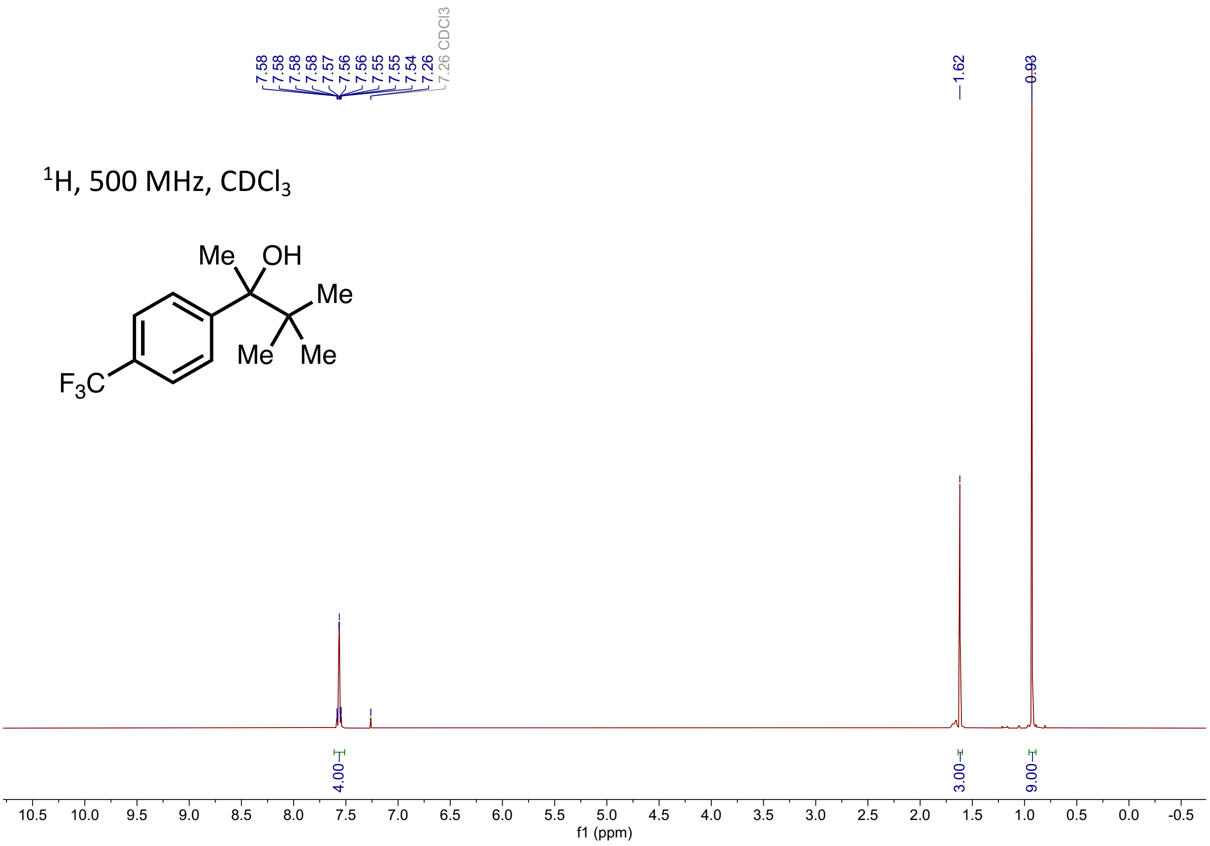
7.58
7.58
7.58
7.58
7.57
7.56
7.56
7.55
7.55
7.54
7.26
7.26 CDCl3
1.62
0.93
^{1}H, 500 MHz, $CDCl_3$
Me
OH
Me
Me
Me
F_3C
4.00
3.00
9.00
f1 (ppm)

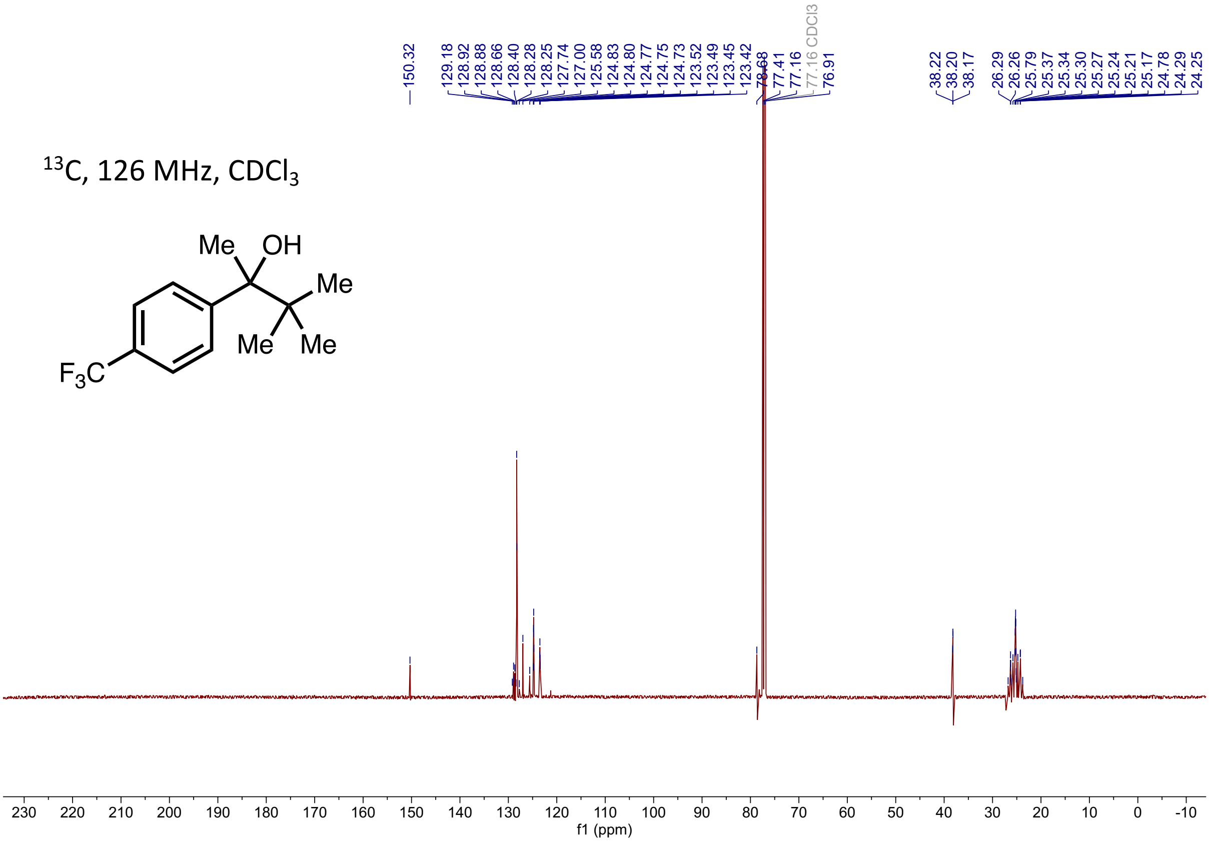
150.32
129.18
128.92
128.88
128.66
128.40
128.28
128.25
127.74
127.00
125.58
124.83
124.80
124.77
124.75
124.73
123.52
123.49
123.45
123.42
78.68
77.41
77.16
77.16 CDCl3
76.91
38.22
38.20
38.17
26.29
26.26
25.79
25.37
25.34
25.30
25.27
25.24
25.21
25.17
24.78
24.29
24.25
^{13}C, 126 MHz, $CDCl_3$
Me
OH
Me
Me
Me
F_3C
f1 (ppm)

^{19}F, 471 MHz, $CDCl_3$

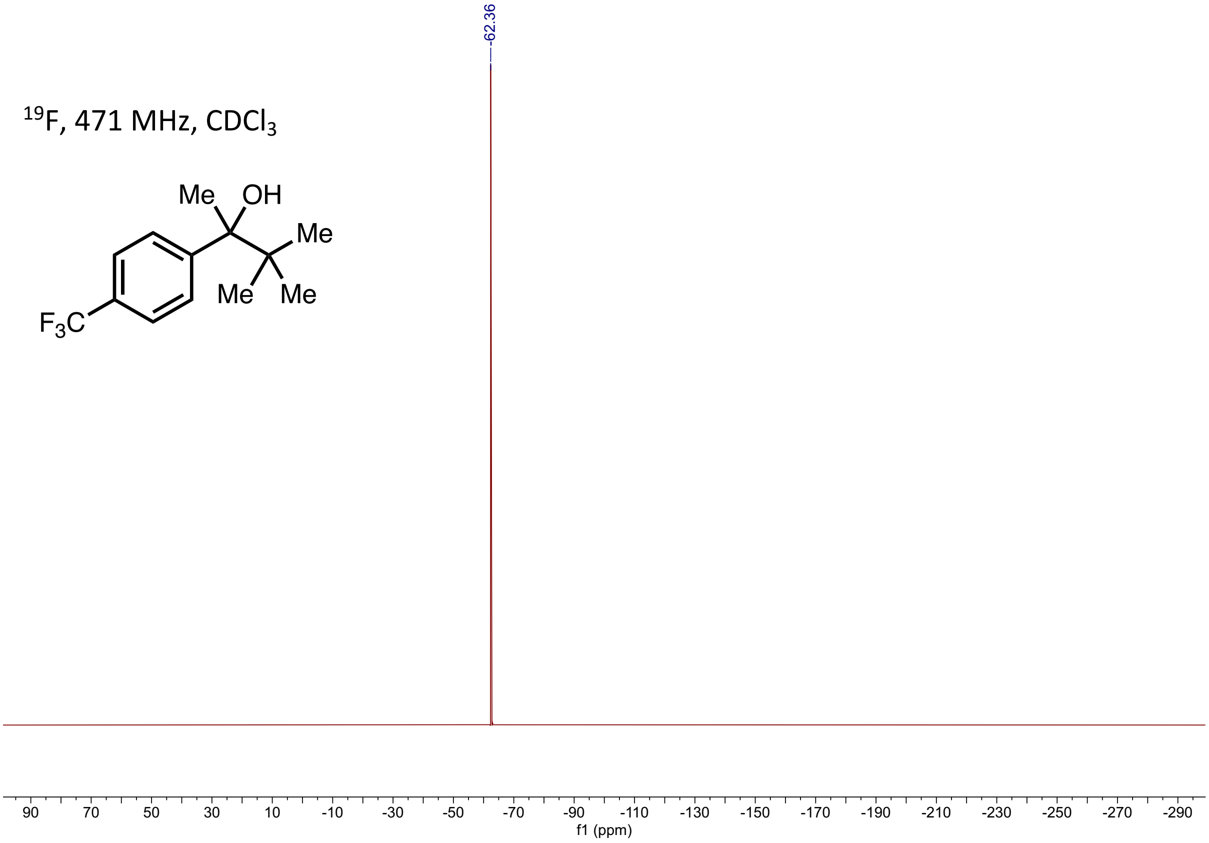

Electrochemical General Procedure and Products Interpretation

Electrochemical General Procedure:

To an oven-dried 10 mL ElectraSyn vial equipped with a magnetic stirrer bar, was added substrate (0.30 mmol) and *n*-Bu_4NPF_6 (232 mg, 0.60 mmol). The threaded glass of the vial was wrapped with PTFE tape and connected to the ElectraSyn cap, which was fitted with a graphite anode and a graphite cathode. The vial was purged with N_2 gas via evacuate-refill cycles (× 3). Dichloromethane (4.5 mL) was added, followed by MeOH (1.5 mL) and the mixture was stirred to facilitate dissolution. The mixture was then purged via bubbling with N_2 gas for 3 minutes. The vial was then connected to an ElectraSyn. Electrolysis at 10 mA was conducted until 3.0 *F* of charge had been passed, under N_2 with continuous stirring. After electrolysis was complete mesitylene internal standard (42 µL, 0.30 mmol) was added to the reaction mixture followed by sampling for crude ^{1}H NMR analysis.

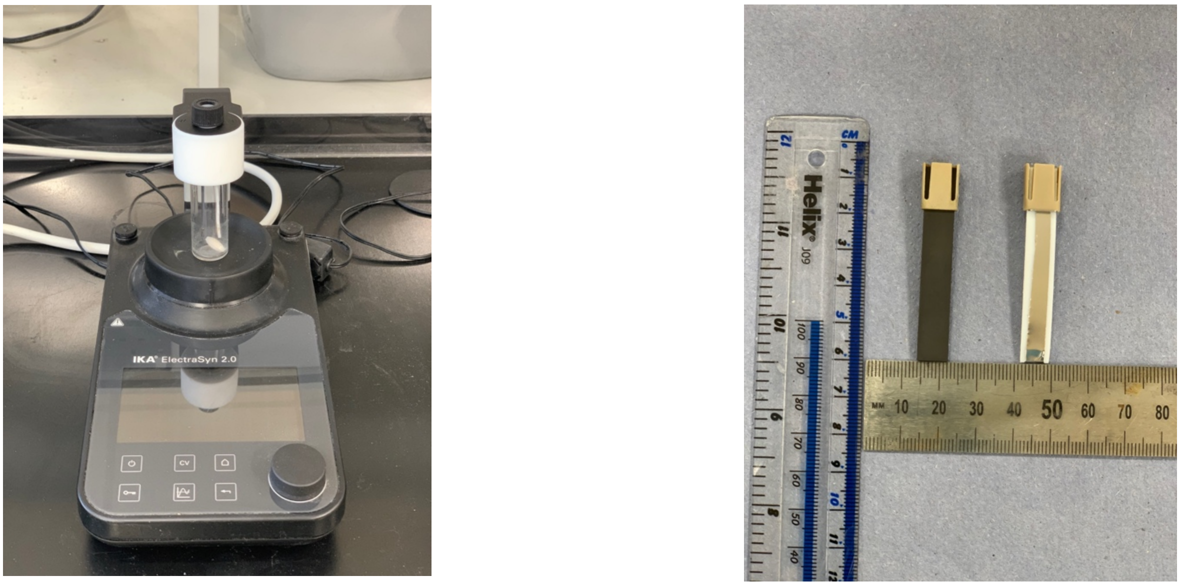

Figure S3. *Left: Reaction set up (IKA ElectraSyn 2.0); Right: Exemplar electrodes (graphite anode and platinum cathode).*

Products Interpretation:

% Yield of the products and return starting material (% RSM) were determined by crude ^{1}H NMR analysis using 1,3,5-trimethylbenzene (42 µL, 0.3 mmol, 1 equiv.) as internal standard.

$$\% \text{ Yield of the Products} = \left(\frac{\text{Compound Integral}}{\text{Standard Integral}}\right) \times \left(\frac{\text{Standard Proton}}{\text{Compound Proton}}\right) \times 100$$

Isolated yield and product characterization are given only when product isolated.

(P1)

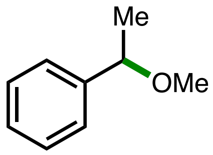

Prepared according to electrochemical general procedure using **S1** & **(*R*)-S1** (49.3 mg, 0.3 mmol), *n*-Bu_4NPF_6 (232 mg, 0.60 mmol), dichloromethane (4.5 mL) and MeOH (1.5 mL). Yield determined by crude ^{1}H NMR using 1,3,5-trimethylbenzene as internal standard: 55% & 54% respectively. Purification by flash column chromatography (eluent = 0 – 5% Et_2O in pentane, silica gel) to afford product as a colourless oil (21.0 mg, 51% yield) and as a racemate with both substrates.

R_f = 0.61 (eluent = 10% Et_2O in hexanes); **^{1}H NMR (500 MHz, $CDCl_3$)** δ 7.31 – 7.19 (m, 5H), 4.26 – 4.22 (q, J = 6.5 Hz, 1H), 3.17 (s, 3H), 1.39 – 1.38 (d, J = 6.5 Hz, 1H); **^{13}C NMR (126 MHz, $CDCl_3$)** δ 143.6, 24.0, 56.6, 79.8, 126.3, 127.6, 128.6.

Data consistent with the literature.[1]

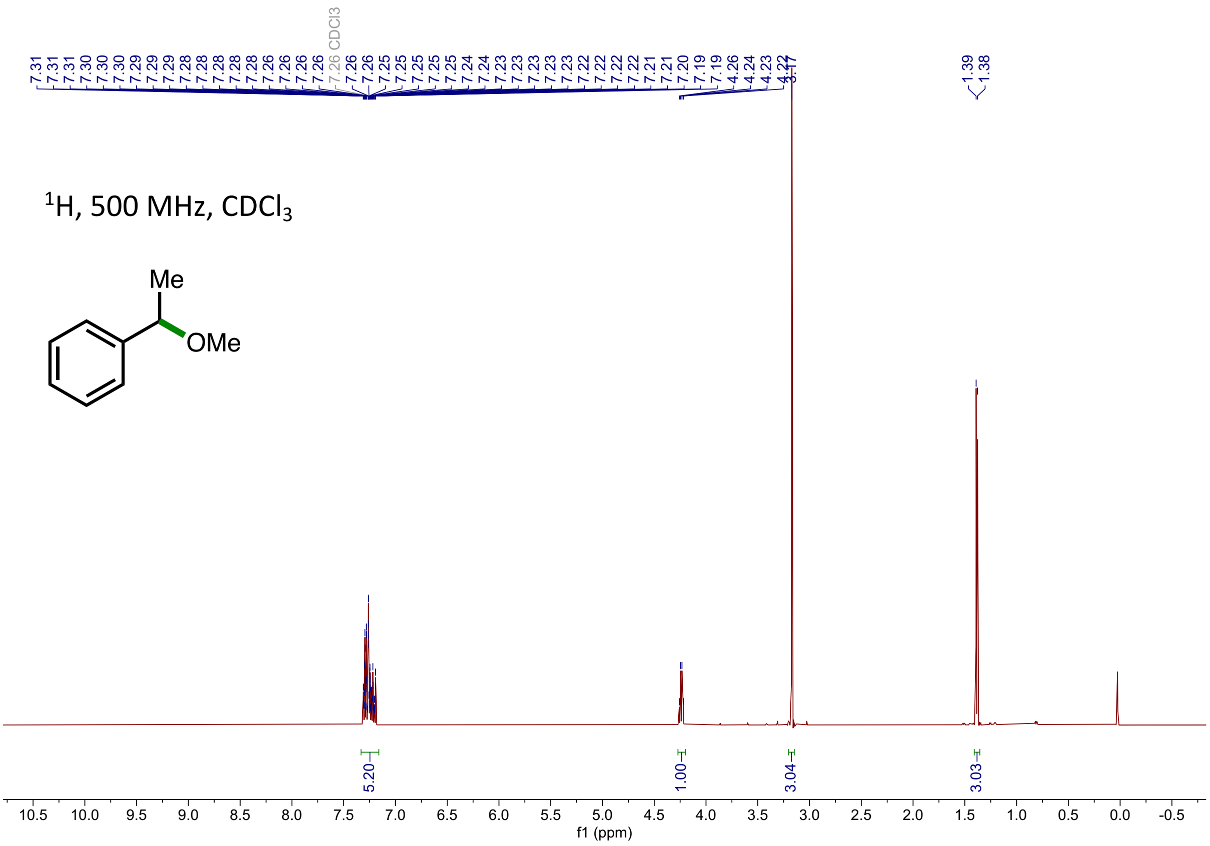

7.31 7.31 7.31 7.30 7.30 7.30 7.29 7.29 7.29 7.28 7.28 7.28 7.28 7.26 7.26 7.26 7.26 CDCl3 7.26 7.26 7.25 7.25 7.25 7.25 7.24 7.24 7.23 7.23 7.23 7.23 7.22 7.22 7.22 7.22 7.21 7.20 7.19 4.26 4.24 4.23 4.22 3.17
1.39 1.38
^{1}H, 500 MHz, CDCl$_3$
Me
OMe
5.20
1.00
3.04
3.03
f1 (ppm)

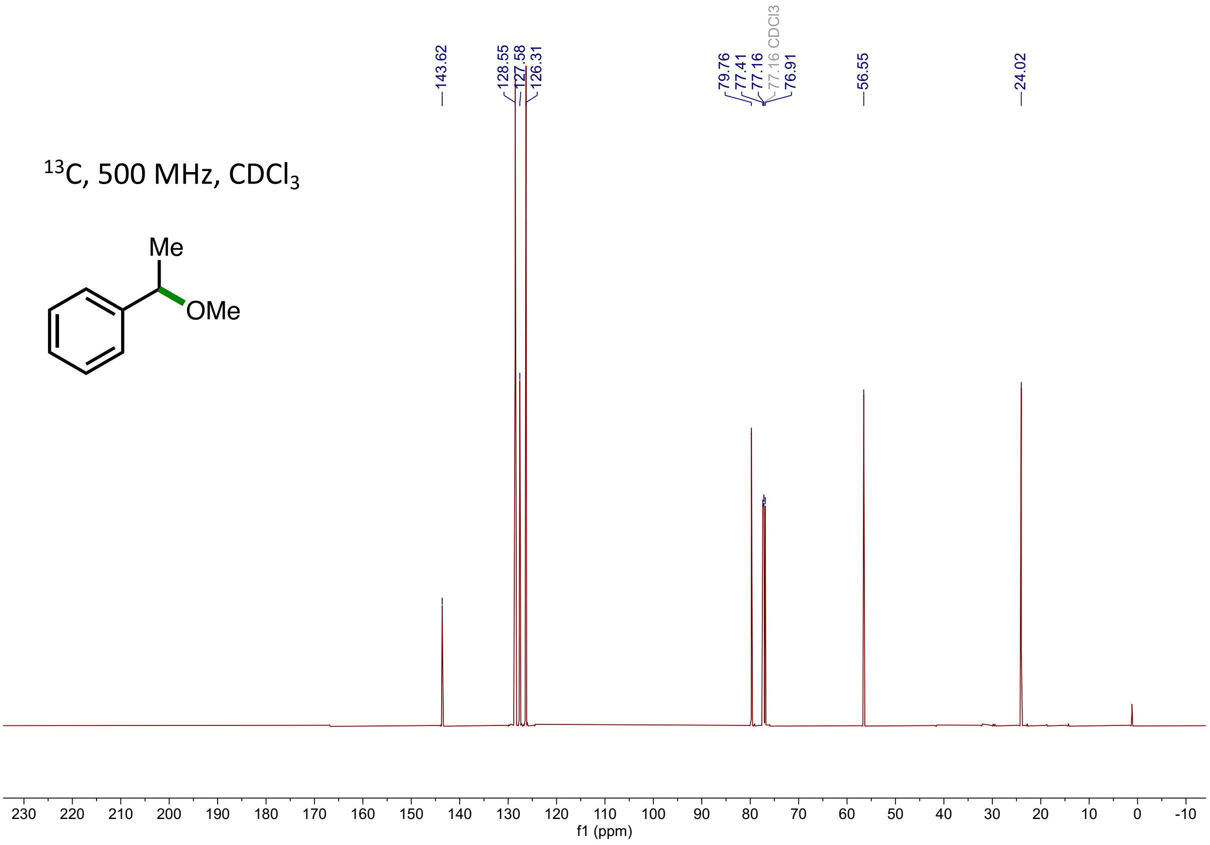

143.62
128.55 127.58 126.31
79.76 77.41 77.16 77.16 CDCl3 76.91
56.55
24.02
^{13}C, 500 MHz, CDCl$_3$
Me
OMe
f1 (ppm)

	Inj. Number	Peak Name	R. Time	Area	Area %
1	1.00	*1	11.60	5355912.50	49.33
2	1.00	*2	12.88	5501105.50	50.67

HPLC Chiralpak IB column, 25 ^{o}C, 99:1 Hexane/IPA, 0.5 mL/min, 30 min; enantiomer 1 = 11.6 min
enantiomer 2 = 12.9 min

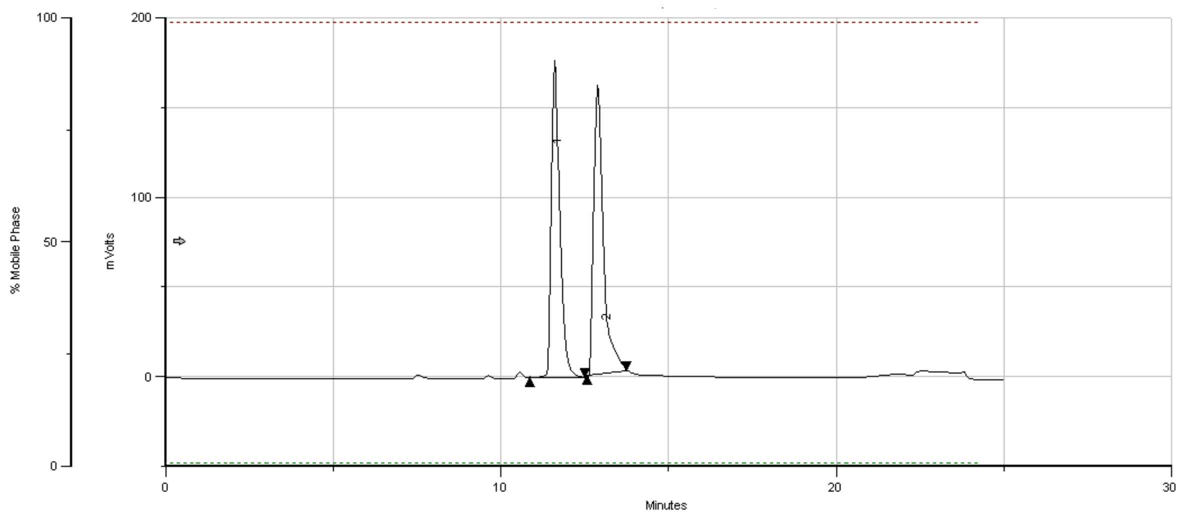

Figure S4. *HPLC trace for racemic **P1**.*

(P4)

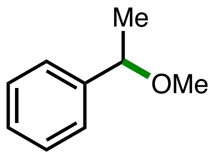

Prepared according to electrochemical general procedure using **S4** (41 mg, 0.3 mmol), *n*-Bu_4NPF_6 (232 mg, 0.60 mmol), dichloromethane (4.5 mL) and MeOH (1.5 mL). Yield determined by crude ^{1}H NMR using mesitylene: 57%

Data consistent with the literature[1] and the data of isolated **P1**.

Selected data for the product:

^{1}H NMR (500 MHz, $CDCl_3$) δ 4.27 – 4.24 (m, 1H).

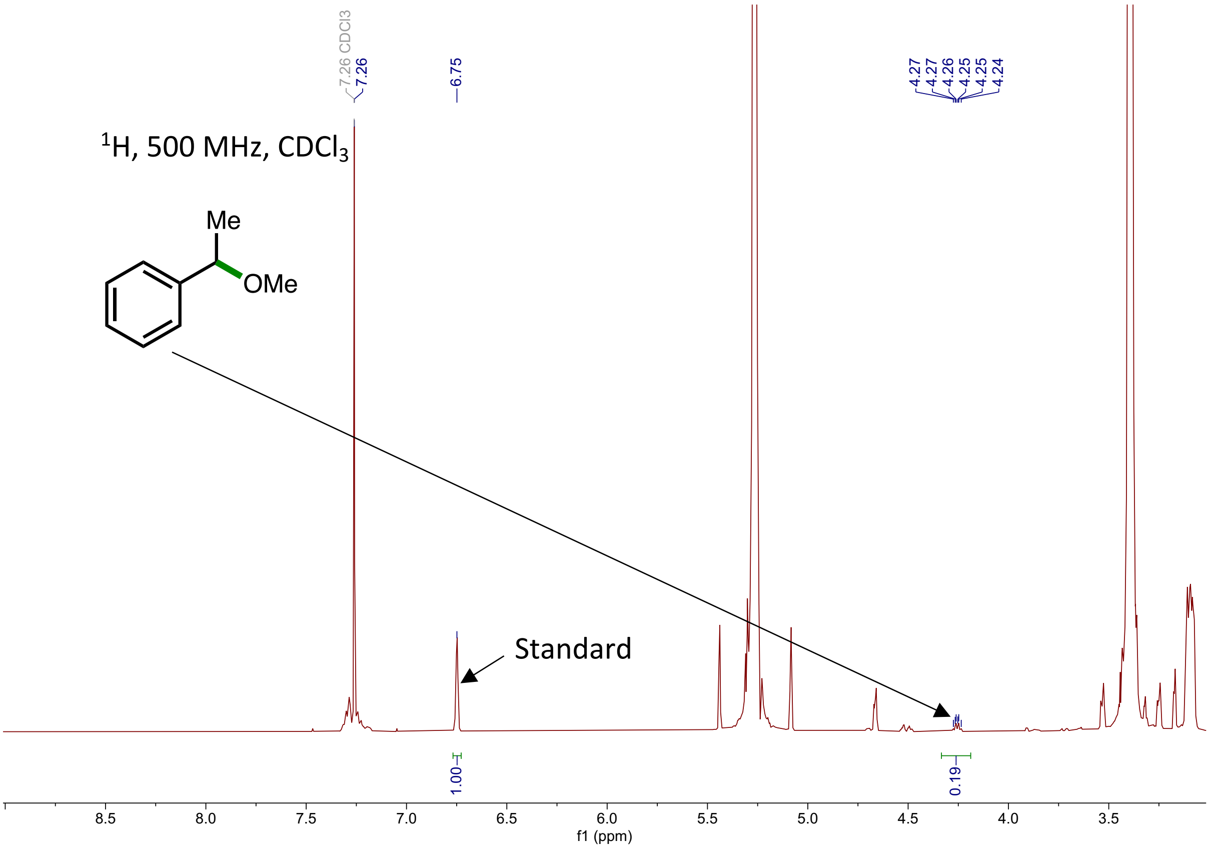

(P5)

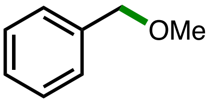

Prepared according to electrochemical general procedure using **S5** (81.7 mg, 0.6 mmol), *n*-Bu_4NPF_6 (232 mg, 0.60 mmol), dichloromethane (4.5 mL) and MeOH (1.5 mL). Yield determined by crude ^{1}H NMR using 1,3,5-trimethylbenzene (42 µL, 0.3 mmol, 0.5 equiv.) as internal standard: 39% with 21% return starting material (RSM).

$$\% \text{ Yield of the Products} = \left(\frac{0.53}{1}\right) \times \left(\frac{1.5\ (0.5\ \text{equiv.})}{2\ (1\ \text{equiv.})}\right) \times 100 = 39\%$$

$$\% \text{ RSM} = \left(\frac{0.28}{1}\right) \times \left(\frac{1.5\ (0.5\ \text{equiv.})}{2\ (1\ \text{equiv.})}\right) \times 100 = 21\%$$

Selected data for the product:

^{1}H NMR (500 MHz, CDCl$_3$) δ 4.44 (s, 2H), 3.37 (s, 3H).

Data consistent with the literature.[1]

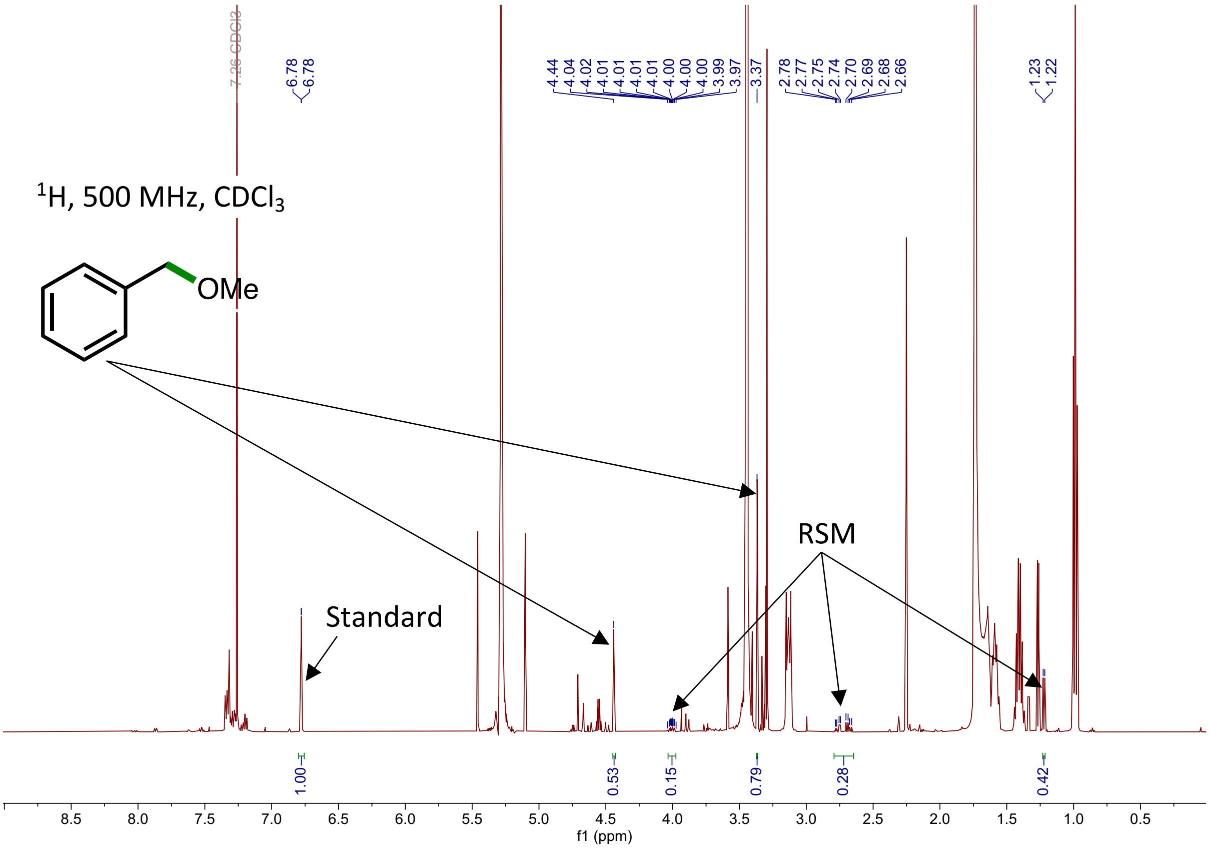

(P6)

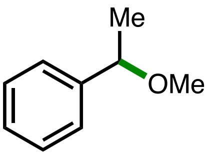

Prepared according to electrochemical general procedure using **S6** (45 mg, 0.3 mmol), *n*-Bu_4NPF_6 (232 mg, 0.60 mmol), dichloromethane (4.5 mL) and MeOH (1.5 mL). Yield determined by crude ^{1}H NMR using mesitylene: 66%

Data consistent with the literature[1] and the data of isolated **P1**.

Selected data for the product:

^{1}H NMR (500 MHz, $CDCl_3$) δ 4.28 – 4.24 (m, 1H).

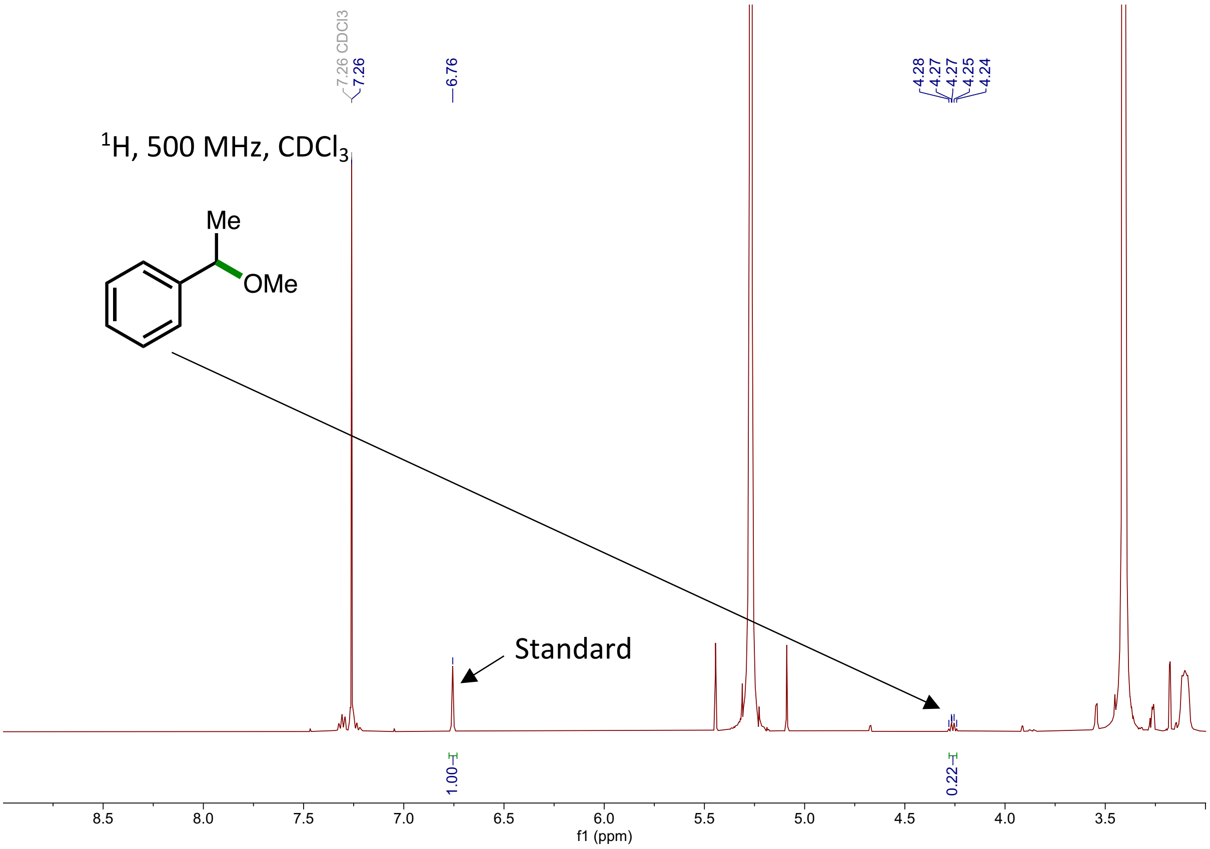

(P7)

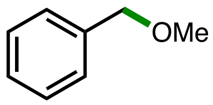

Prepared according to electrochemical general procedure using **S7** (45 mg, 0.3 mmol), *n*-Bu_4NPF_6 (232 mg, 0.60 mmol), dichloromethane (4.5 mL) and MeOH (1.5 mL). Yield determined by crude ^1H NMR using 1,3,5-trimethylbenzene (42 µL, 0.3 mmol, 0.5 equiv.) as internal standard: 60%

Selected data for the product:

^1H NMR (500 MHz, $CDCl_3$) δ 4.39 (s, 2H).

Data consistent with the literature.[1]

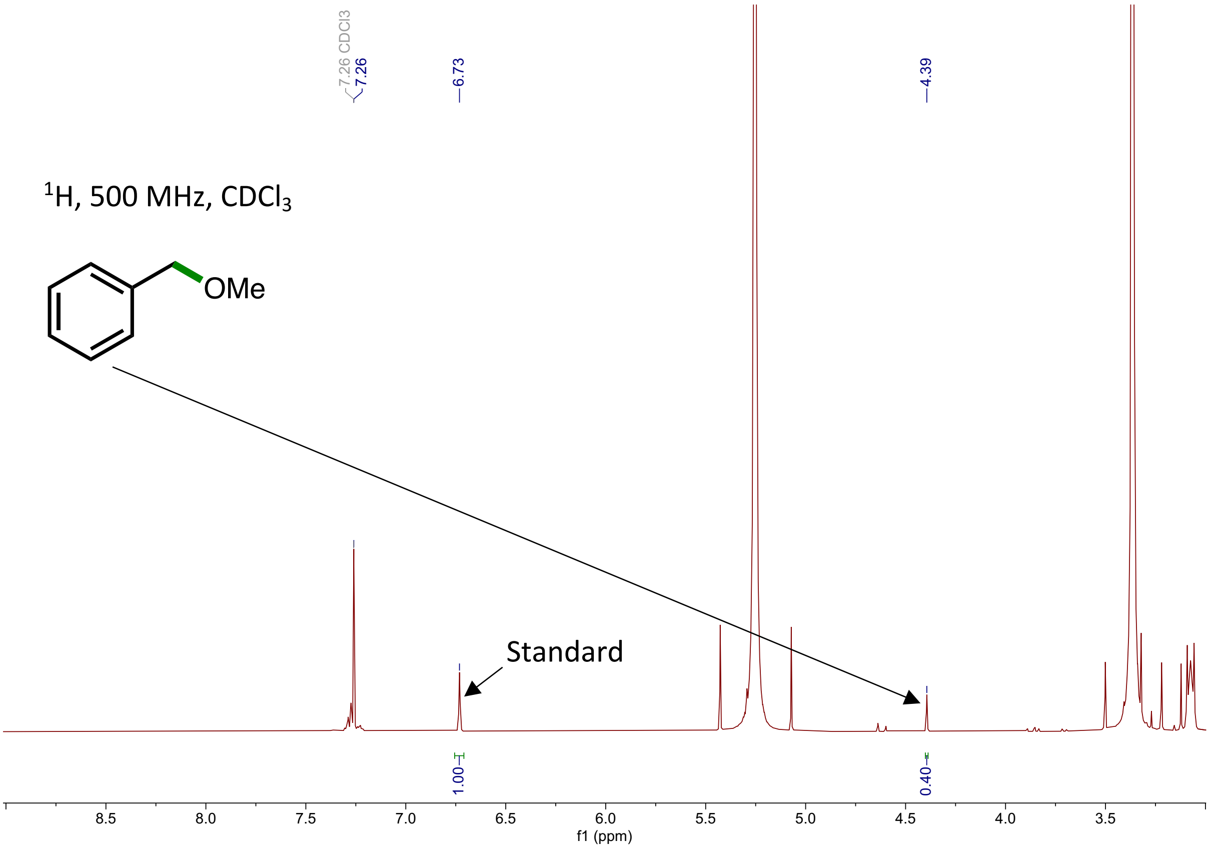

(P9)

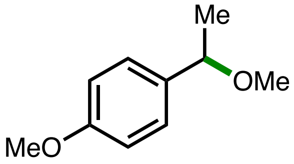

Prepared according to electrochemical general procedure using **S9** (58.3 mg, 0.3 mmol), *n*-Bu_4NPF_6 (232 mg, 0.60 mmol), dichloromethane (4.5 mL) and MeOH (1.5 mL). Yield determined by crude ^{1}H NMR using 1,3,5-trimethylbenzene as internal standard: 54%

Selected data for the product:

^{1}H NMR (300 MHz, $CDCl_3$) δ 7.22 – 7.19 (d, *J* = 8.6 Hz, 2H), 6.88 – 6.85 (d, *J* = 8.6 Hz, 2H), 3.78 (s, 2H).

Selected data for 4-methoxyacetophenone (Observed in 28% yield):

^{1}H NMR (300 MHz, $CDCl_3$) δ 7.93 – 7.90 (d, *J* = 8.9 Hz, 2H), 6.93 – 6.90 (d, *J* = 8.9 Hz, 2H), 3.85 (s, 3H).

Data consistent with the literature.[8,9]

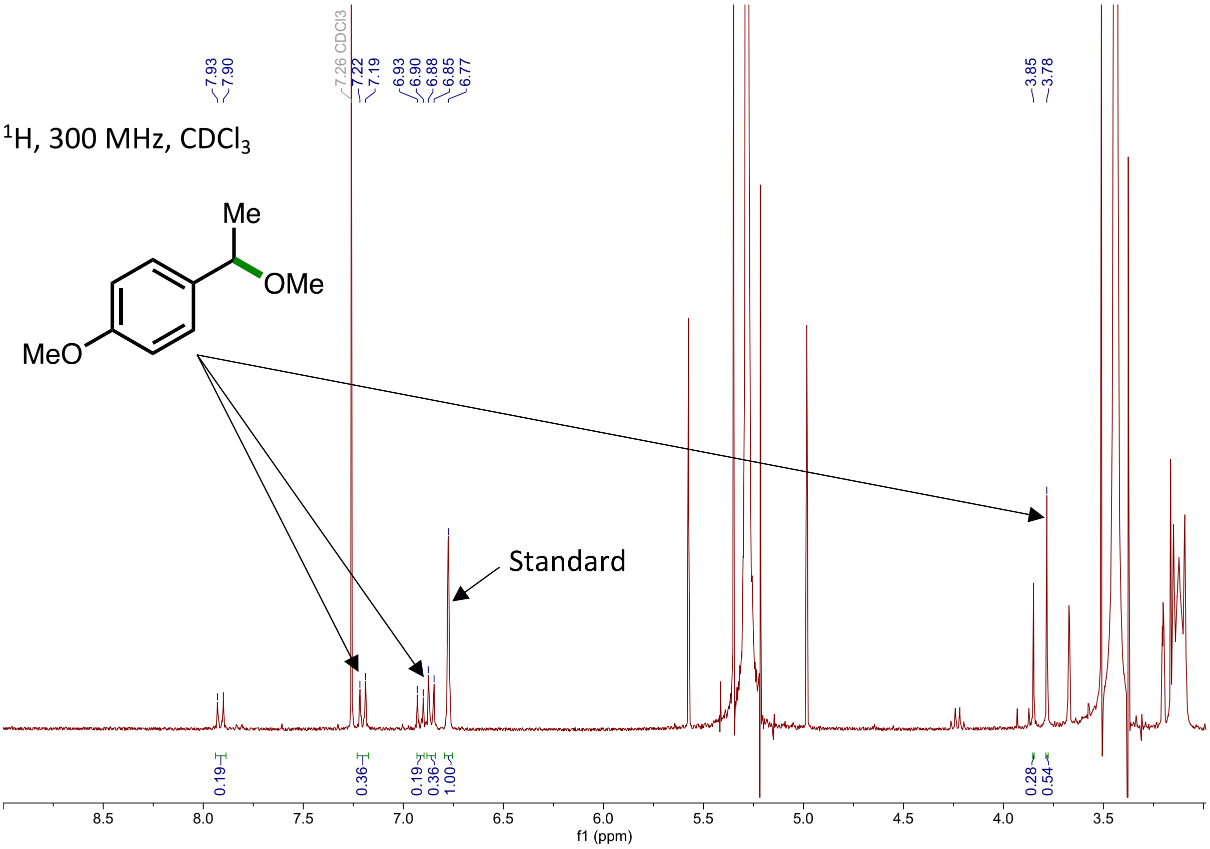

(P11)

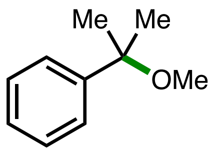

Prepared according to electrochemical general procedure using **S11** (53.5 mg, 0.3 mmol), *n*-Bu_4NPF_6 (232 mg, 0.60 mmol), dichloromethane (4.5 mL) and MeOH (1.5 mL). Yield determined by crude ^{1}H NMR using 1,3,5-trimethylbenzene as internal standard: 78%

Selected data for the product:

^{1}H NMR (300 MHz, $CDCl_3$) δ 7.43 – 7.25 (m, 5H), 3.07 (s, 3H), 1.53 (s, 6H).

Data consistent with the literature.[10]

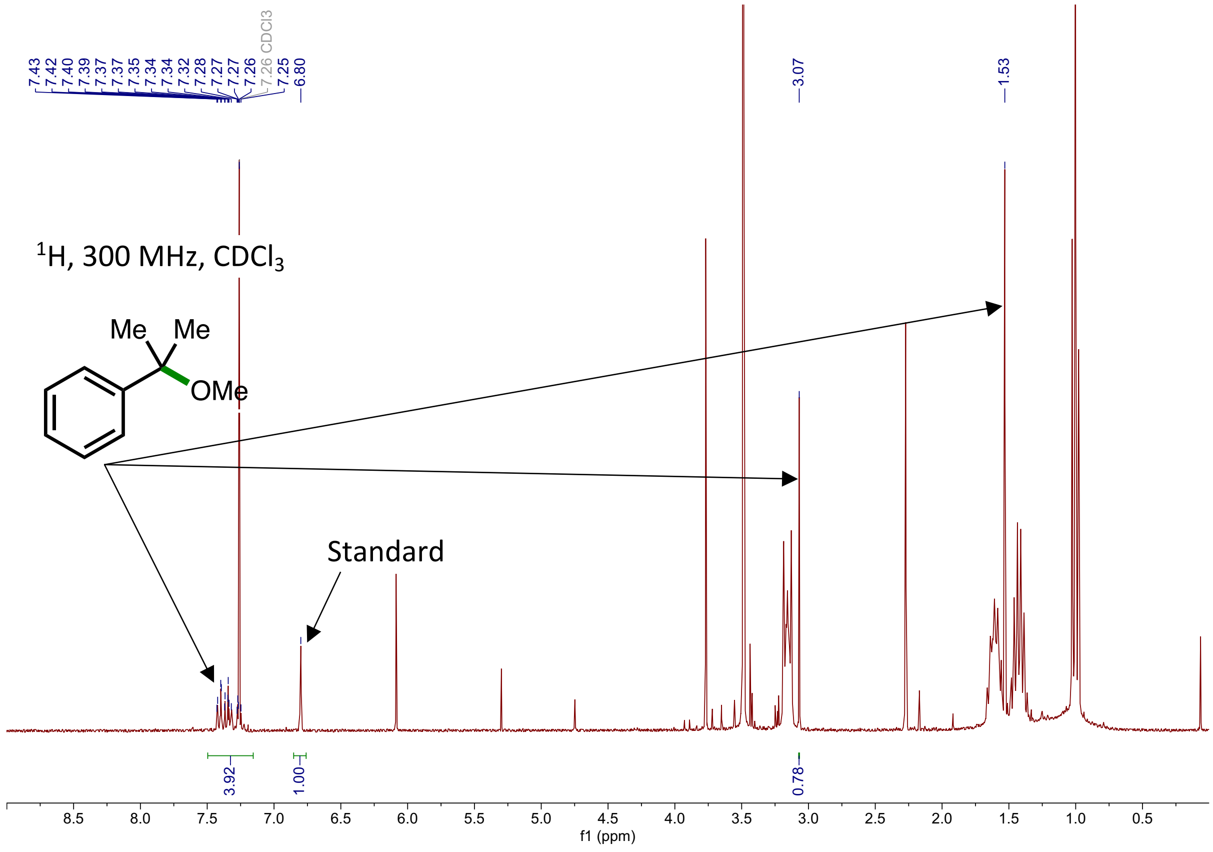

(P12)

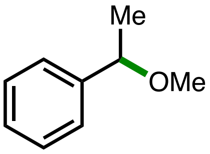

Prepared according to electrochemical general procedure using **S12** (53.5 mg, 0.3 mmol), *n*-Bu_4NPF_6 (232 mg, 0.60 mmol), dichloromethane (4.5 mL) and MeOH (1.5 mL). Yield determined by crude ^1H NMR using 1,3,5-trimethylbenzene as internal standard: 72%

Selected data for the product:

^{1}H NMR (300 MHz, $CDCl_3$) δ 4.31 – 4.25 (q, *J* = 6.5 Hz, 1H).

Data consistent with the literature[1] and the data of isolated **P1**.

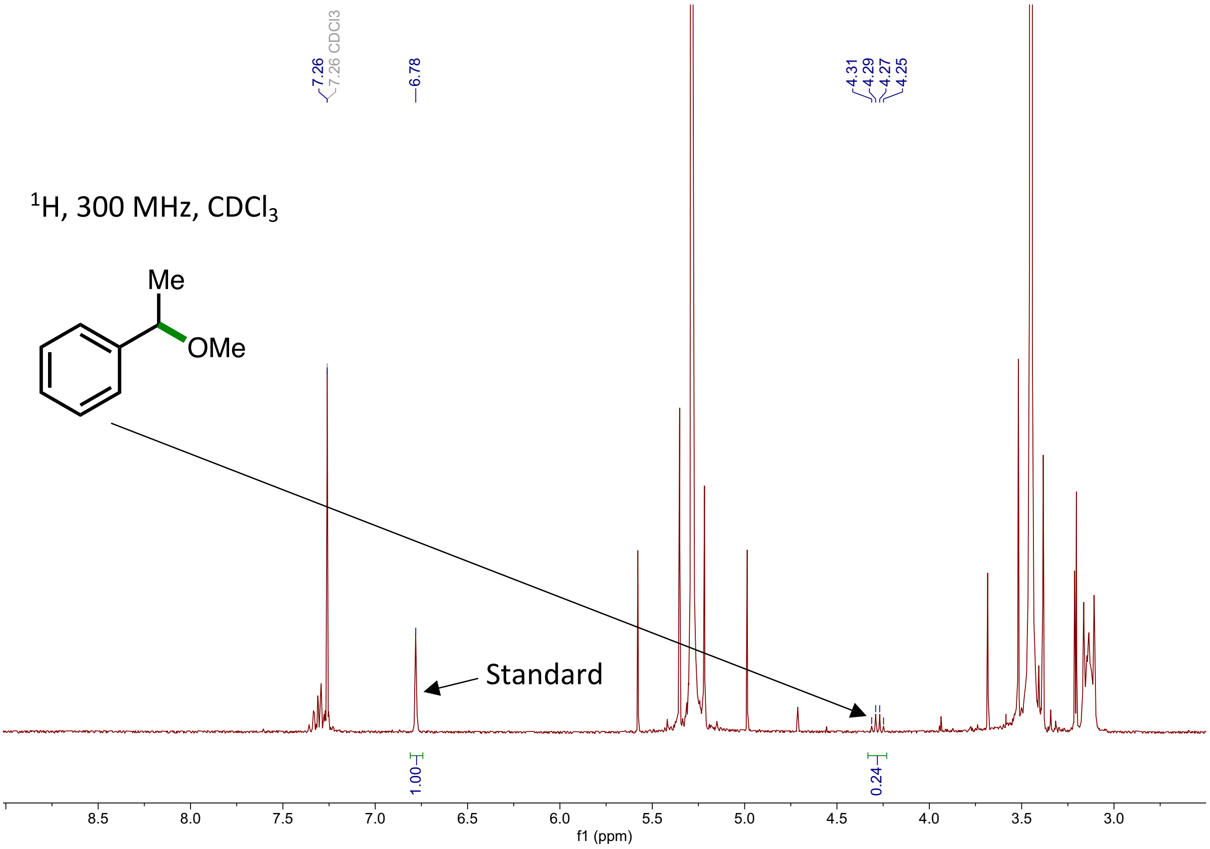

(P13)

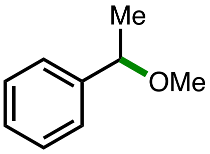

Prepared according to electrochemical general procedure using **S13** (61.6 mg, 0.3 mmol), *n*-Bu_4NPF_6 (232 mg, 0.60 mmol), dichloromethane (4.5 mL) and MeOH (1.5 mL). Yield determined by crude ^{1}H NMR using 1,3,5-trimethylbenzene as internal standard: 53%

Selected data for the product:

^{1}H NMR (500 MHz, $CDCl_3$) δ 7.35 – 7.32 (m, 2H), 4.30 – 4.26 (q, *J* = 6.5 Hz, 1H).

Data consistent with the literature[1] and the data of isolated **P1**.

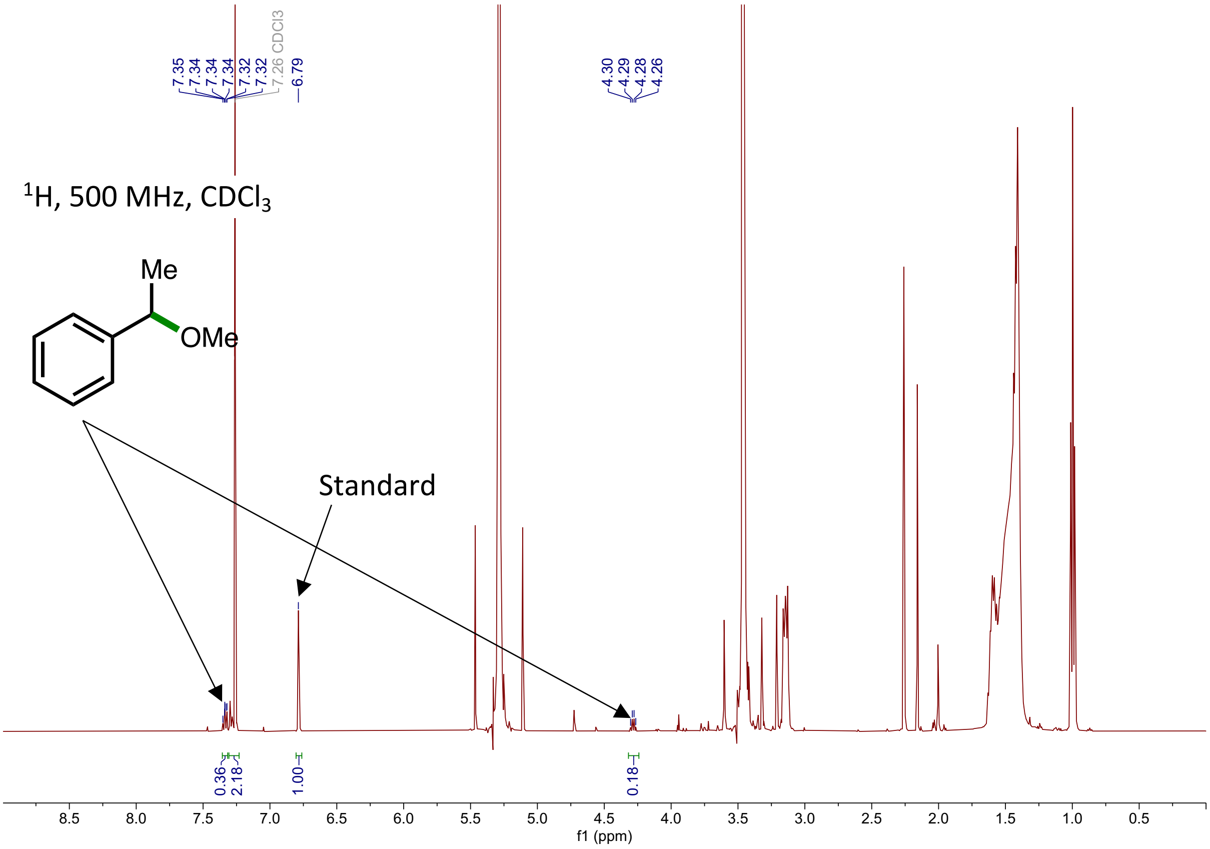

(P16)

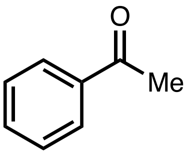

Prepared according to electrochemical general procedure using **S16** (45 mg, 0.3 mmol), *n*-Bu_4NPF_6 (232 mg, 0.60 mmol), dichloromethane (4.5 mL) and MeOH (1.5 mL). Yield determined by crude ^1H NMR using 1,3,5-trimethylbenzene as internal standard: 24% with 73% return starting material (RSM).

Selected data for the product:

^1H NMR (300 MHz, $CDCl_3$) δ 7.96 – 7.93 (m, 2H), 2.60 (s, 3H).

Data consistent with the literature.[11]

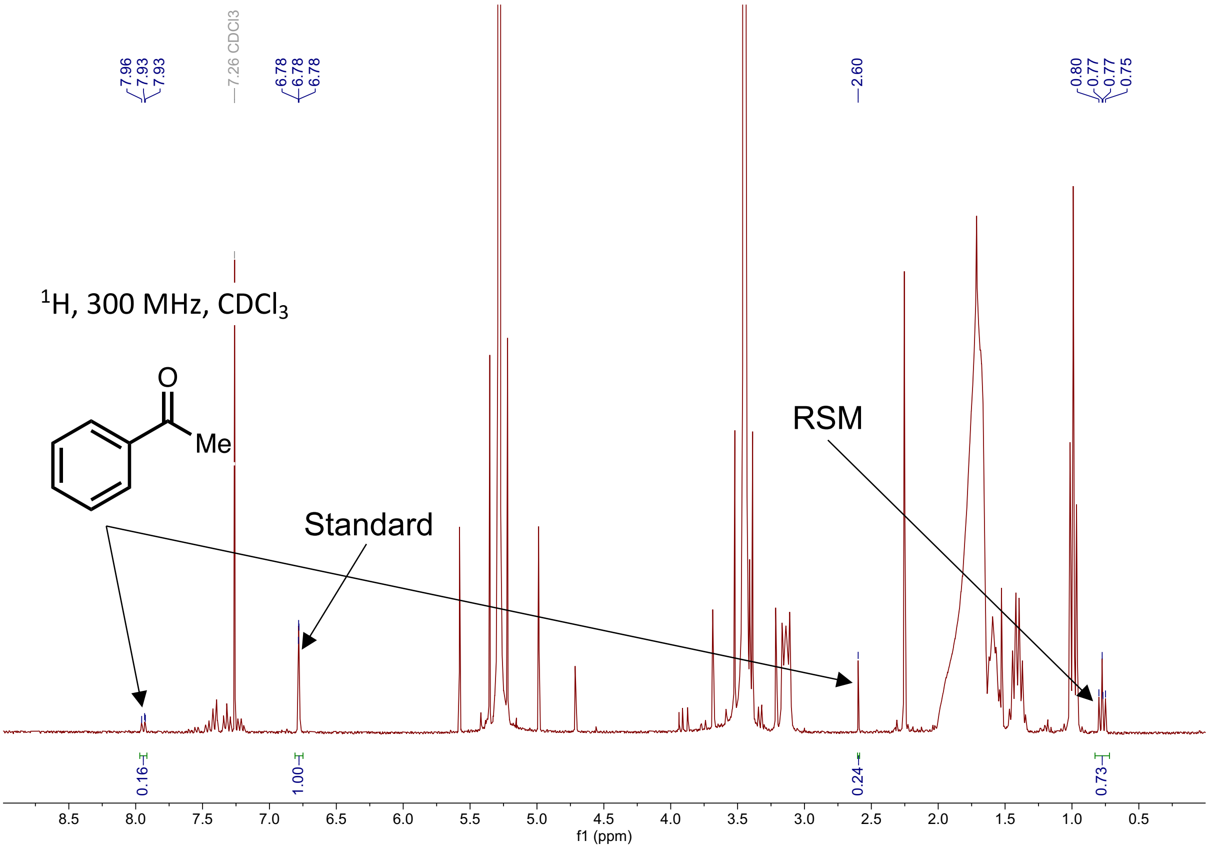

(P17)

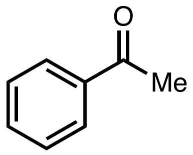

Prepared according to electrochemical general procedure using **S17** (49.3 mg, 0.3 mmol), *n*-Bu_4NPF_6 (232 mg, 0.60 mmol), dichloromethane (4.5 mL) and MeOH (1.5 mL). Yield determined by crude ^{1}H NMR using 1,3,5-trimethylbenzene as internal standard: 55%

Selected data for the product:

^{1}H NMR (500 MHz, $CDCl_3$) δ 7.95 – 7.93 (m, 2H), 7.56 – 7.53 (m, 1H), 7.46 – 7.43 (m, 2H), 2.59 (s, 3H).

Selected data for 2-methoxypropane (Observed in 18% yield):

^{1}H NMR (300 MHz, $CDCl_3$) δ 1.13 – 1.12 (d, *J* = 6.1 Hz, 6H).

Data consistent with the literature.[11]

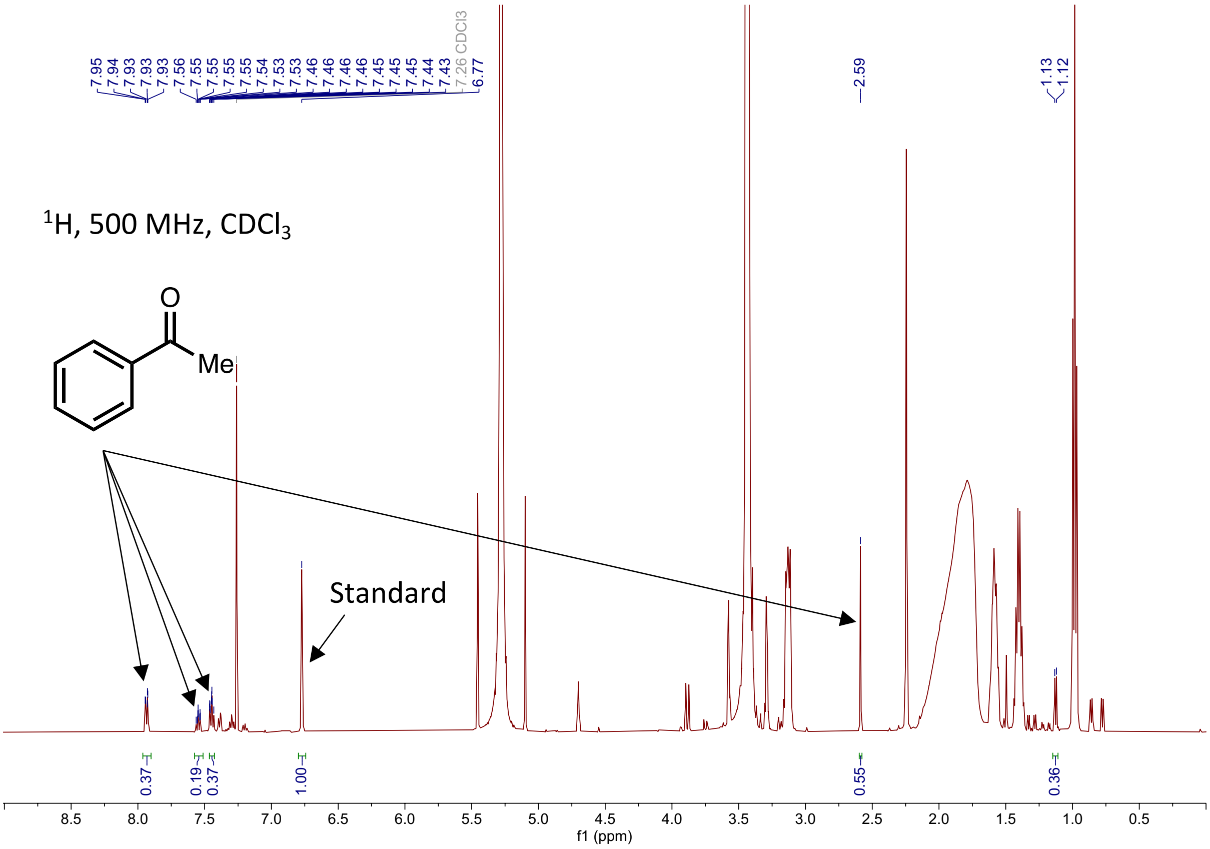

(P18)

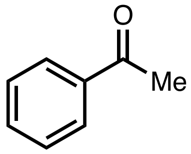

Prepared according to electrochemical general procedure using **S18** (53.5 mg, 0.3 mmol), *n*-Bu_4NPF_6 (232 mg, 0.60 mmol), dichloromethane (4.5 mL) and MeOH (1.5 mL). Yield determined by crude ^{1}H NMR using 1,3,5-trimethylbenzene as internal standard: 65%

Selected data for the product:

^{1}H NMR (300 MHz, $CDCl_3$) δ 7.96 – 7.93 (m, 2H), 7.56 – 7.54 (m, 1H), 7.48 – 7.45 (m, 2H), 2.60 (s, 3H).

Selected data for 2-methoxy-2-methylpropane (Observed in 52% yield):

^{1}H NMR (300 MHz, $CDCl_3$) δ 3.20 (s, 3H), 1.17 (s, 9H).

Data consistent with the literature.[11]

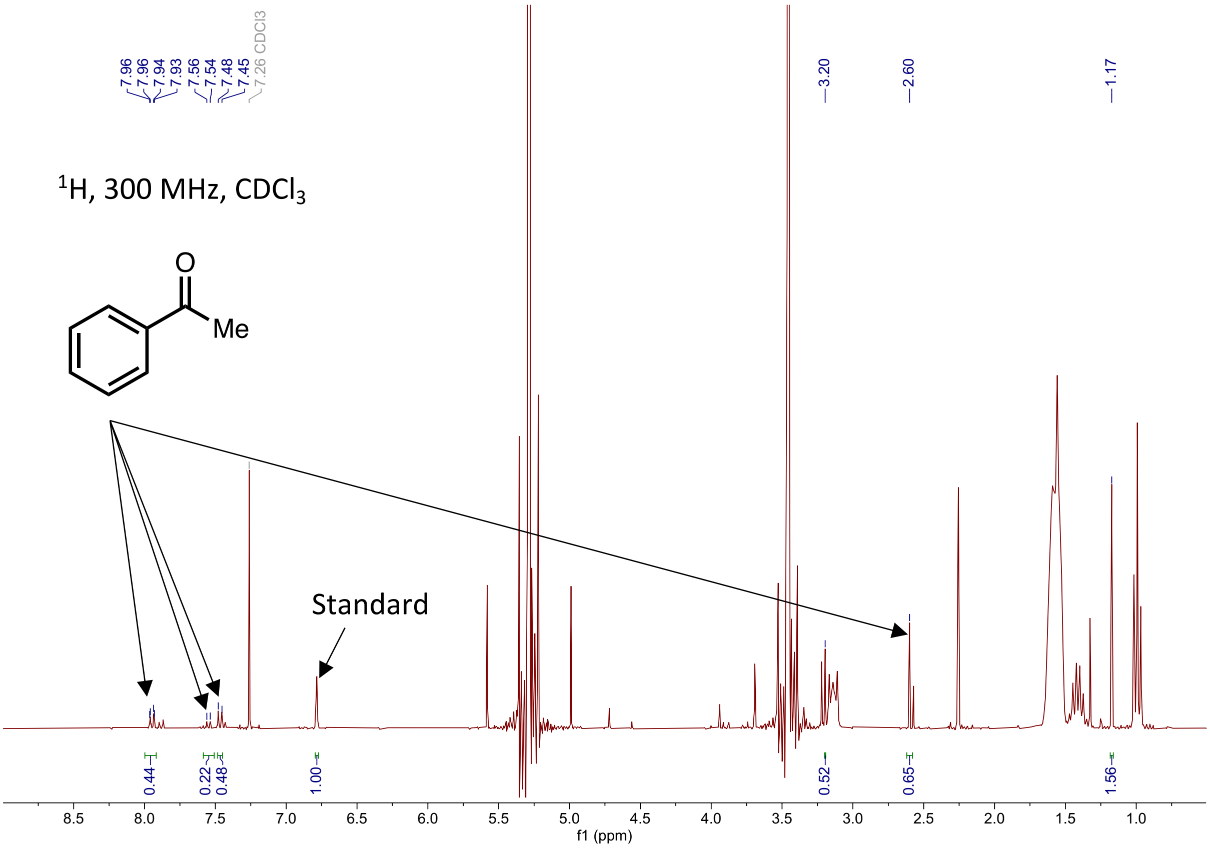

(P19)

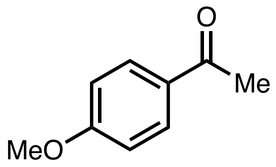

Prepared according to electrochemical general procedure using **S19** (62.5 mg, 0.3 mmol), *n*-Bu_4NPF_6 (232 mg, 0.60 mmol), dichloromethane (4.5 mL) and MeOH (1.5 mL). Yield determined by crude ^{1}H NMR using 1,3,5-trimethylbenzene as internal standard: 86%

Selected data for the product:

^{1}H NMR (300 MHz, $CDCl_3$) δ 7.94 – 7.91 (d, *J* = 8.9 Hz, 2H), 6.94 – 6.91 (d, *J* = 8.9 Hz, 2H), 3.86 (s, 3H), 2.55 (s, 3H).

Selected data for 2-methoxy-2-methylpropane (Observed in 64% yield):

^{1}H NMR (300 MHz, $CDCl_3$) δ 3.20 (s, 3H), 1.17 (s, 9H).

Data consistent with the literature.[9]

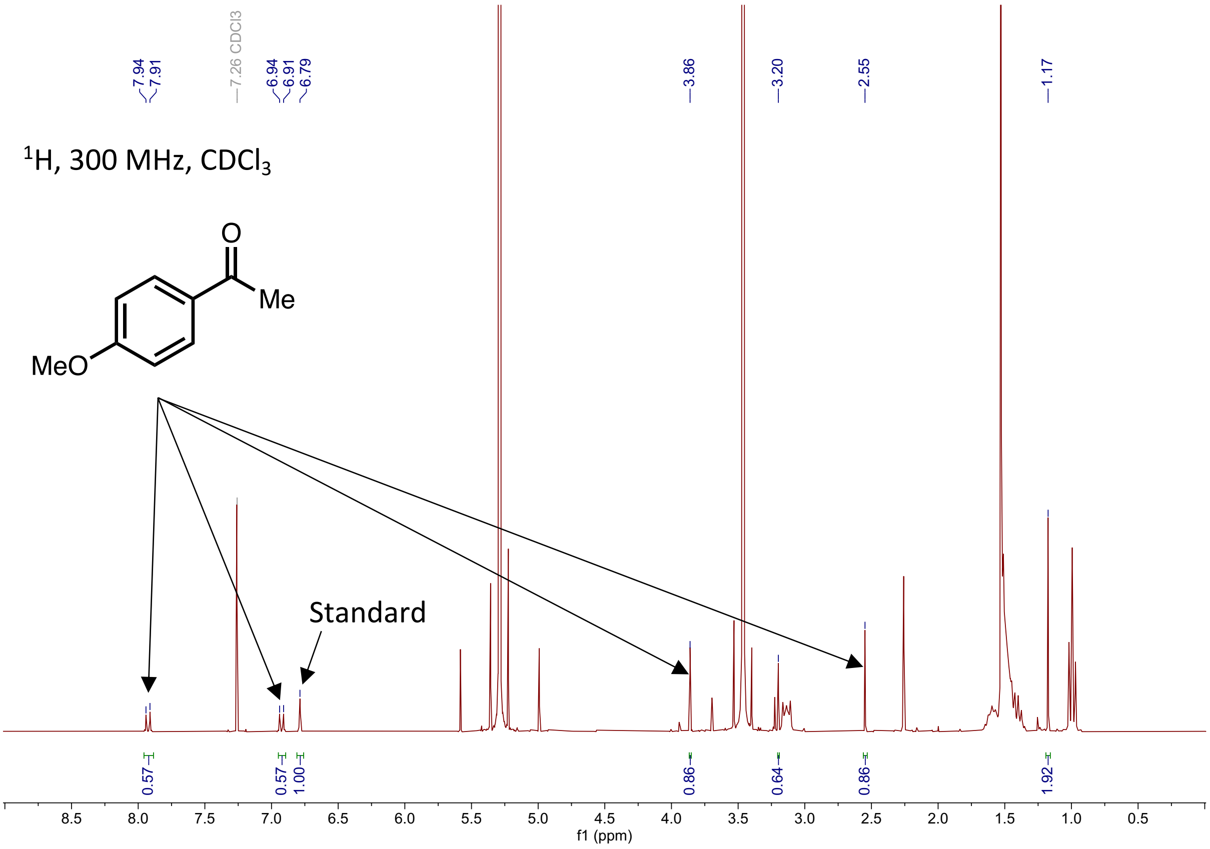

Cyclic Voltammetry Studies

Oxidative Potential Window (0.0 – 2.5 V vs. Fc/Fc$^+$).

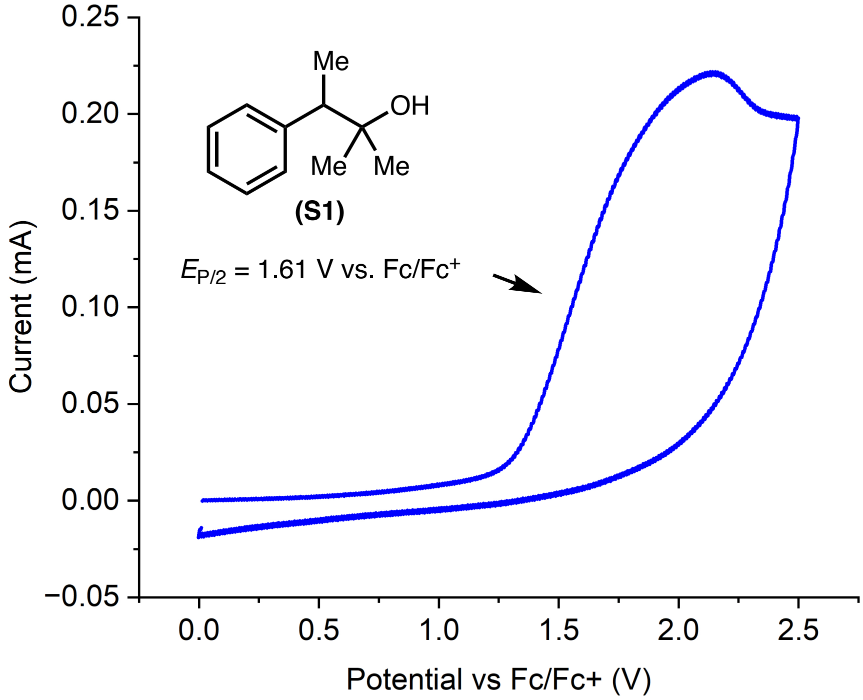

Figure S5. *CV of Compound* ***S1*** *(5 mM in DCM), n-Bu$_4$PF$_6$ (0.1 M), Scan Rate: 100 mV/s*

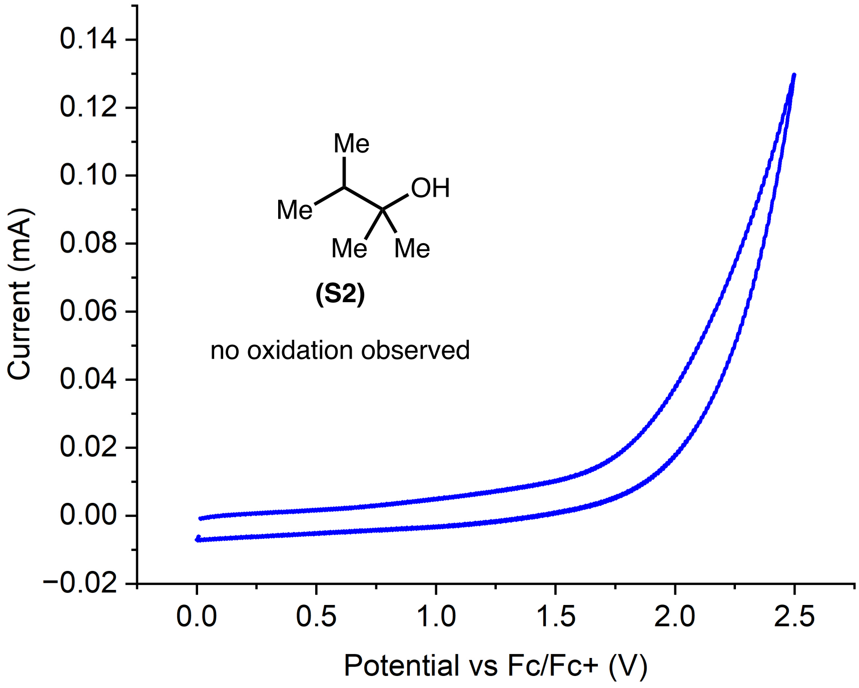

Figure S6. *CV of Compound **S2** (5 mM in DCM), n-Bu$_4$PF$_6$ (0.1 M), Scan Rate: 100 mV/s*

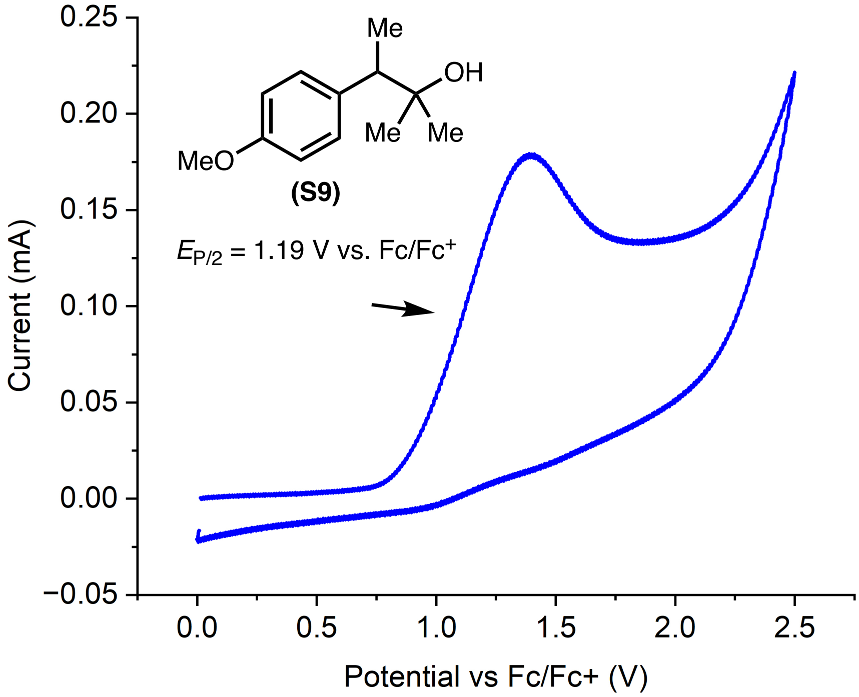

Figure S7. *CV of Compound **S9** (5 mM in DCM), n-Bu$_4$PF$_6$ (0.1 M), Scan Rate: 100 mV/s*

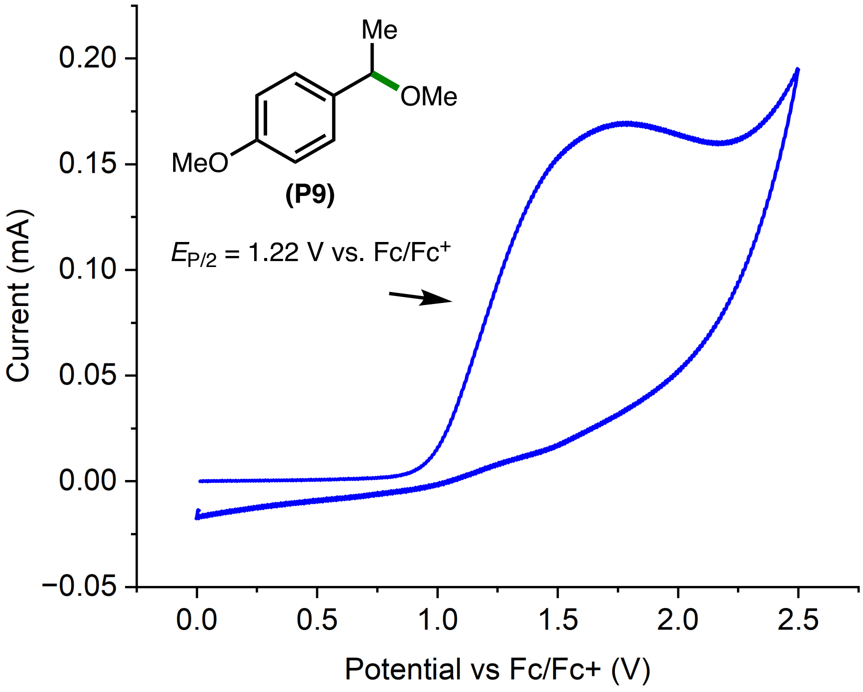

Figure S8. *CV of Compound **P9** (5 mM in DCM), n-Bu$_4$PF$_6$ (0.1 M), Scan Rate: 100 mV/s*

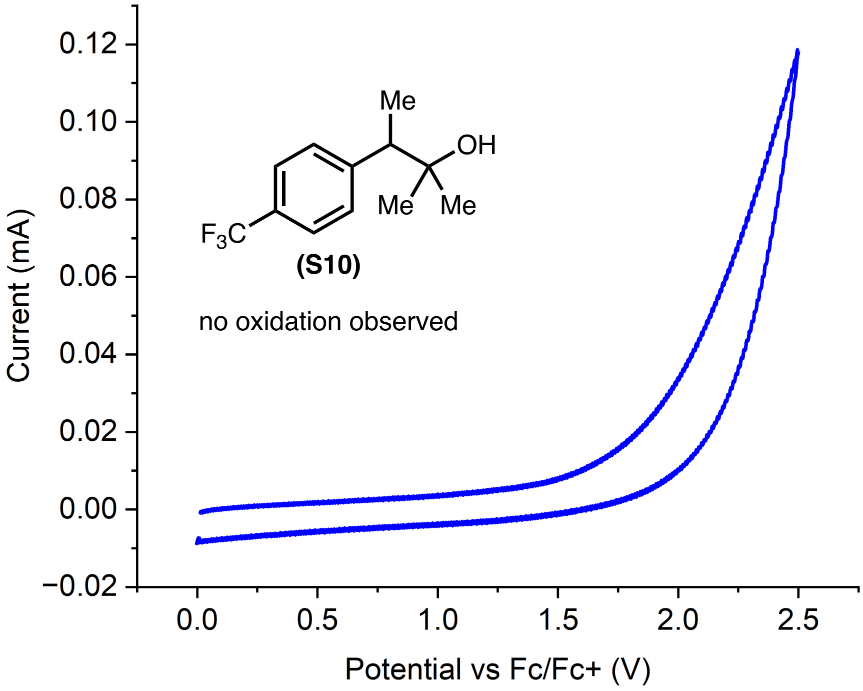

Figure S9. *CV of Compound **S10** (5 mM in DCM), n-Bu$_4$PF$_6$ (0.1 M), Scan Rate: 100 mV/s*

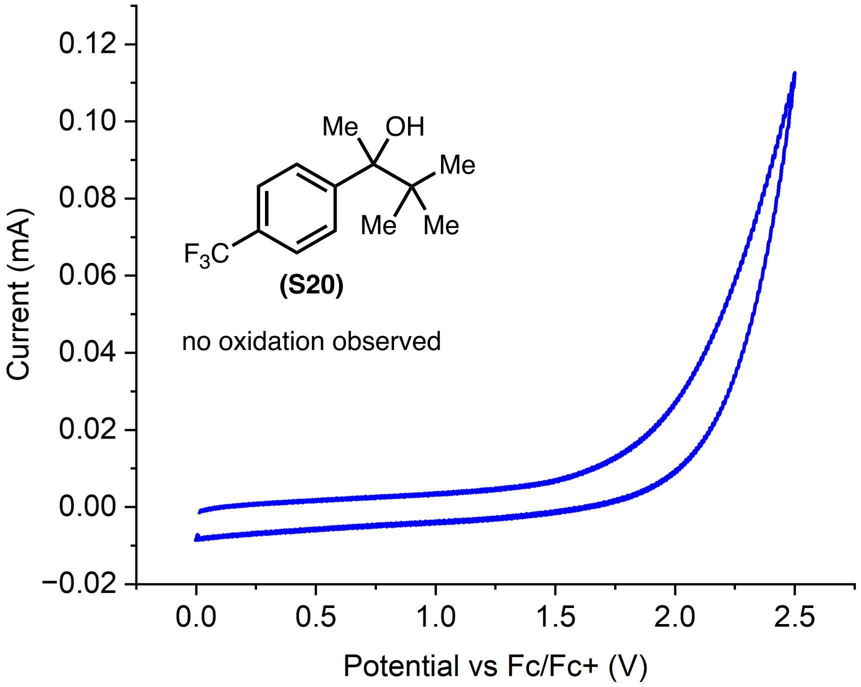

Figure S10. *CV of Compound **S20** (5 mM in DCM), n-Bu$_4$PF$_6$ (0.1 M), Scan Rate: 100 mV/s*

Computational Studies

Computational Methods:

All calculations performed in this work were geometry optimisations at the ωB97X-D/Def2-TZVPP level of theory using the Gaussian 16, Revision C.01 quantum chemistry code.[12] All calculations were performed in both methanol and dichloromethane solvents in the IEF-PCM implicit solvent model.[13] Quasi-harmonic Gibbs free energies using Grimme's quasi-harmonic treatment of entropy[14] were extracted using the GoodVibes Python library[15] from the optimised structure files with concentration set to 1.0 mol/L and temperature set to 298.15 K. The spin density plot shown in Scheme 3 in the main manuscript was visualised with an IsoValue set to 0.005 e^{-}/au^{3}. All structure visualisations in the main manuscript were performed using GaussView 6[16] and CYLview 1.0b[17]. All structure visualisations in this supporting information were performed using GaussView 6.

Mechanism Barriers:

In order to explore which of two mechanisms, "S_N1-like" or "S_N2-like", was most likely to occur, we calculated the activation barriers of the corresponding transition states of these two mechanisms for the reactant species shown in Figure S11.

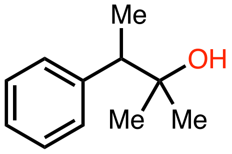

Figure S11. *The reactant structure from which the barriers of the S_N1-like and S_N2-like mechanisms were calculated.*

To begin, we built the three major conformations of the reactant structure that arise from the rotations of the central bond and optimised the radical cation form of each of those three conformers (which are shown in Figure S12). These were used as the reactant species for the S_N1-like mechanism barrier calculations.

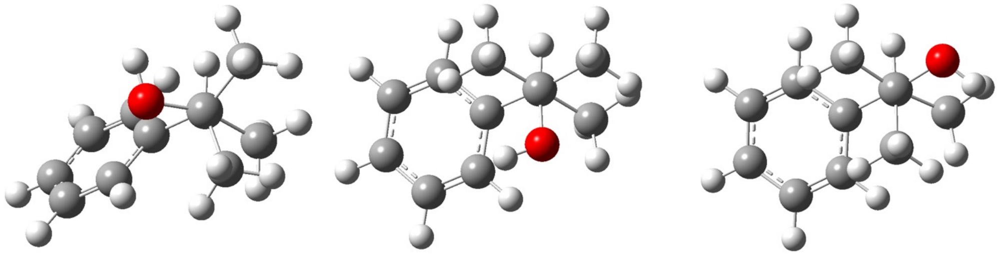

Figure S12. *The three conformations of the optimised radical cation reactant structure. From left to right, these conformations are named "1", "2" and "3", and have relative free energies of: 3.54, 0.0 and 0.208 kcal/mol in dichloromethane and 3.25, 0.287 and 0.0 kcal/mol in methanol.*

We also constructed pre-reaction complexes for the S_N2-like mechanism by including a methoxy anion with each of the three reactant conformers and optimising these structures to become the "reactant" species for the barrier calculations. An example pre-reaction complex structure is shown in Figure S13.

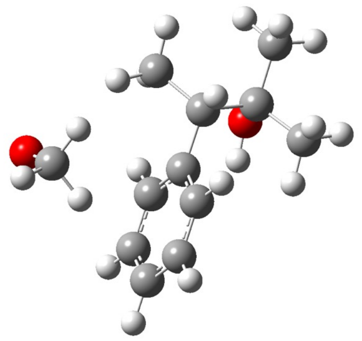

Figure S13. *An example pre-reaction complex conformation (specifically, conformer 2 from Figure S12) for the S_N2-like mechanism.*

Finally, we optimised the three conformers of the transition states of both mechanisms and an example of an S_N1-like transition state is shown in Figure S14 and an example of an S_N2-like transition state is shown in Figure S15. We report the barriers of the two reaction mechanisms in Scheme 4 of the main manuscript as the free energy difference between the lowest energy transition state conformer and the lowest energy reactant conformer (or the lowest energy pre-reaction complex conformer, in the case of the S_N2-like mechanism).

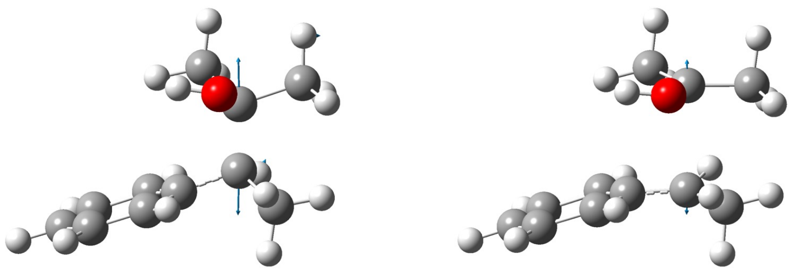

Figure S14. *A visualisation of the imaginary vibrational frequency of an example optimised transition state of the S_N1-like mechanism (specifically conformer 2 from Figure S12).*

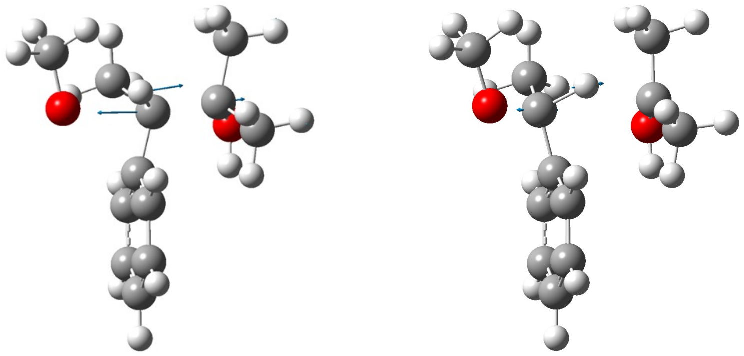

Figure S15. *A visualisation of the imaginary vibrational frequency of an example optimised transition state of the S_N2-like mechanism (specifically conformer 2 from Figure S12).*

Fragments:

In order to explore the likely locations of the radical electron and cationic centre following the mesolytic cleavage of the radical cation reactant structure, we selected a set of eight molecules and fragmented them according to the highlighted bonds in Figure S16. We generated initial 3D geometries of the fragments using the RDKit Python library[18] and then optimised both the radical and cationic forms of both fragments of each molecule (see Schemes 5, 7 and 8 in the main manuscript).

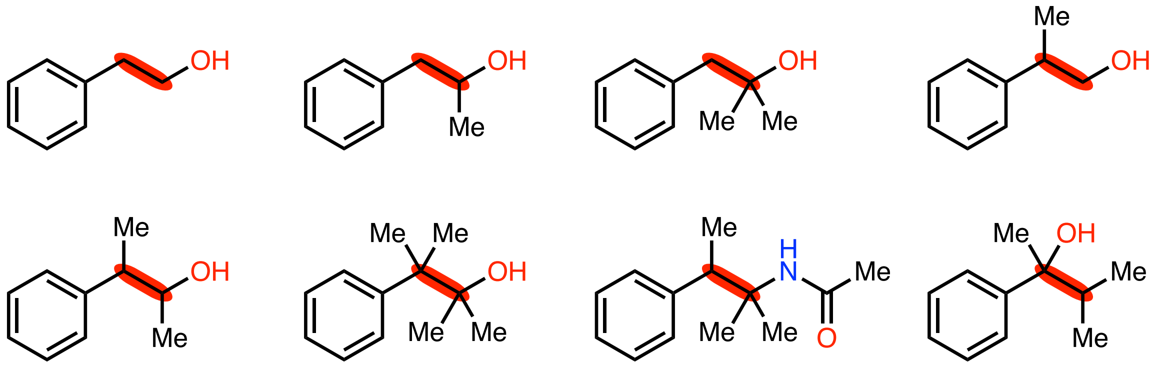

Figure S16. *The selection of molecules that were fragmented into radical and cationic substructures. The bond highlighted in red indicates the location of the fragmentation. See Schemes 5, 7 and 8 in the main manuscript.*

Coordinates and Energies:

Sn1-like Reactant Conformer 1 (Dichloromethane)

Number of imaginary frequencies = 0

Electronic energy = -503.821452

Zero-point correction = 0.246476

Enthalpy = -503.561527

Temperature entropy product = 0.048819

Temperature quasi-harmonic entropy product = 0.047911

Gibbs free energy = -503.610346

Quasi-harmonic Gibbs free energy = -503.609438

Cartesian Coordinates

C	1.07053	-1.00607	0.41572
C	0.67882	0.29050	-0.04985
C	2.38262	-1.36498	0.42506
H	2.68190	-2.34240	0.77336
C	1.69966	1.19703	-0.48853
C	3.36455	-0.45650	-0.02293
H	1.40332	2.17680	-0.83662
H	4.40611	-0.74680	-0.00837
C	3.00649	0.83190	-0.48473
H	3.77459	1.50893	-0.82786
H	0.31924	-1.70238	0.74453
C	-0.72153	0.79131	-0.06720
C	-0.88572	1.72612	1.15794
H	-0.73443	1.18268	2.08852
H	-0.17957	2.55328	1.11976
H	-1.89119	2.14265	1.15249

C	-1.84391	-0.27590	-0.20398
C	-3.06001	0.36971	-0.86214
H	-3.85857	-0.36532	-0.95439
H	-3.43457	1.20795	-0.27543
H	-2.81185	0.73928	-1.85907
C	-2.26091	-0.90454	1.12106
H	-2.95772	-1.71591	0.91836
H	-1.42432	-1.30923	1.68734
H	-2.76411	-0.17299	1.75047
O	-1.36914	-1.34124	-1.02797
H	-1.36983	-1.05671	-1.94345
H	-0.78939	1.43866	-0.94844

Sn1-like Reactant Conformer 1 (Methanol)

Number of imaginary frequencies = 0

Electronic energy = -503.827690

Zero-point correction = 0.246427

Enthalpy = -503.567820

Temperature entropy product = 0.048788

Temperature quasi-harmonic entropy product = 0.047898

Gibbs free energy = -503.616608

Quasi-harmonic Gibbs free energy = -503.615718

Cartesian Coordinates

C	1.07226	-1.00872	0.41300
C	0.67980	0.28760	-0.04946
C	2.38459	-1.36532	0.42262
H	2.68578	-2.34283	0.76889
C	1.69962	1.19662	-0.48681
C	3.36637	-0.45398	-0.02296

H	1.40147	2.17633	-0.83332
H	4.40807	-0.74336	-0.00774
C	3.00711	0.83382	-0.48307
H	3.77408	1.51271	-0.82474
H	0.32194	-1.70699	0.73972
C	-0.72068	0.78846	-0.06567
C	-0.88350	1.72069	1.16150
H	-0.73072	1.17538	2.09072
H	-0.17626	2.54688	1.12314
H	-1.88849	2.13835	1.15791
C	-1.84583	-0.27519	-0.20445
C	-3.06275	0.37925	-0.85332
H	-3.86455	-0.35231	-0.94605
H	-3.43108	1.21567	-0.26004
H	-2.81678	0.75287	-1.84908
C	-2.25951	-0.91238	1.11730
H	-2.96055	-1.71968	0.91175
H	-1.42189	-1.32386	1.67689
H	-2.75762	-0.18356	1.75388
O	-1.38086	-1.33756	-1.03804
H	-1.33306	-1.02766	-1.94434
H	-0.78840	1.43746	-0.94540

Sn1-like Reactant Conformer 2 (Dichloromethane)

Number of imaginary frequencies = 0

Electronic energy = -503.826170

Zero-point correction = 0.245866

Enthalpy = -503.566558

Temperature entropy product = 0.049745

Temperature quasi-harmonic entropy product = 0.048522

Gibbs free energy = -503.616303

SUPPORTING INFORMATION

Quasi-harmonic Gibbs free energy = -503.615080

Cartesian Coordinates

C	1.17081	-1.00765	-0.54954
C	0.63020	-0.10244	0.42740
C	2.46916	-0.90180	-0.94199
H	2.87973	-1.57210	-1.68224
C	1.48140	0.88957	0.99592
C	3.28557	0.09415	-0.37436
H	1.07451	1.55964	1.73990
H	4.31858	0.17886	-0.68265
C	2.77761	0.99005	0.60276
H	3.43005	1.74160	1.02237
H	0.52224	-1.75687	-0.97450
C	-0.79076	-0.19599	0.84549
C	-1.12133	-1.62995	1.29327
H	-1.23127	-2.29738	0.44153
H	-0.34482	-2.01913	1.94969
H	-2.05409	-1.62532	1.85255
C	-1.74719	0.32514	-0.28364
C	-1.38014	1.75897	-0.66226
H	-2.06566	2.12499	-1.42715
H	-1.45109	2.42445	0.19853
H	-0.36863	1.81174	-1.06656
C	-3.19328	0.25622	0.19020
H	-3.84325	0.69170	-0.57067
H	-3.50707	-0.77245	0.35447
H	-3.33252	0.82161	1.11154
O	-1.54698	-0.53786	-1.39434
H	-2.20894	-0.34619	-2.05990
H	-0.93424	0.48000	1.68829

Sn1-like Reactant Conformer 2 (Methanol)

Number of imaginary frequencies = 0

Electronic energy = -503.831898

Zero-point correction = 0.245945

Enthalpy = -503.572385

Temperature entropy product = 0.049136

Temperature quasi-harmonic entropy product = 0.048049

Gibbs free energy = -503.621520

Quasi-harmonic Gibbs free energy = -503.620434

Cartesian Coordinates

C	-1.21065	-1.05951	0.51166
C	-0.62498	-0.16041	-0.43019
C	-2.51134	-0.91485	0.87883
H	-2.96319	-1.58766	1.59254
C	-1.42742	0.89740	-0.96860
C	-3.28615	0.13264	0.32459
H	-0.97702	1.57642	-1.67881
H	-4.31841	0.24476	0.62597
C	-2.73111	1.03399	-0.60601
H	-3.34310	1.82220	-1.01838
H	-0.60578	-1.84980	0.93012
C	0.78202	-0.29469	-0.83413
C	1.17480	-1.74582	-1.13235
H	1.31524	-2.31804	-0.21749
H	0.41370	-2.23324	-1.73913
H	2.10609	-1.76293	-1.69317
C	1.74238	0.35203	0.28857
C	1.33023	1.79051	0.57589

H	2.03984	2.23646	1.27180
H	1.32760	2.38444	-0.33739
H	0.33542	1.84607	1.02269
C	3.17092	0.29561	-0.23203
H	3.81590	0.82842	0.46621
H	3.52405	-0.73081	-0.30136
H	3.25164	0.76658	-1.21059
O	1.70435	-0.42826	1.46020
H	1.04906	-0.08046	2.06652
H	0.94193	0.32227	-1.71853

Sn1-like Reactant Conformer 3 (Dichloromethane)

Number of imaginary frequencies = 0

Electronic energy = -503.826552

Zero-point correction = 0.246082

Enthalpy = -503.567024

Temperature entropy product = 0.048642

Temperature quasi-harmonic entropy product = 0.047725

Gibbs free energy = -503.615666

Quasi-harmonic Gibbs free energy = -503.614749

Cartesian Coordinates

C	-1.27635	-1.13463	0.40527
C	-0.62895	-0.16236	-0.41359
C	-2.58375	-0.98113	0.75635
H	-3.07920	-1.72136	1.36715
C	-1.37197	0.98952	-0.82111
C	-3.29771	0.15360	0.32239
H	-0.88007	1.73318	-1.43212
H	-4.33264	0.27316	0.61189

C	-2.68185	1.13447	-0.47336
H	-3.24799	1.99377	-0.80111
H	-0.72654	-2.00244	0.73737
C	0.75479	-0.31675	-0.84658
C	1.18415	-1.74449	-1.16493
H	1.17498	-2.40377	-0.29932
H	0.52135	-2.16952	-1.91674
H	2.19089	-1.73541	-1.57678
C	1.78218	0.37293	0.26987
C	1.70160	-0.34345	1.60739
H	2.42629	0.11041	2.28283
H	0.71241	-0.24081	2.05040
H	1.93653	-1.40407	1.52222
C	1.46833	1.85221	0.39708
H	2.21912	2.31053	1.03932
H	1.50171	2.34560	-0.57317
H	0.48907	2.01135	0.84577
O	3.05119	0.27825	-0.28799
H	3.45048	-0.56728	-0.07390
H	0.93136	0.32119	-1.71253

Sn1-like Reactant Conformer 3 (Methanol)

Number of imaginary frequencies = 0

Electronic energy = -503.832636

Zero-point correction = 0.246068

Enthalpy = -503.573118

Temperature entropy product = 0.048949

Temperature quasi-harmonic entropy product = 0.047773

Gibbs free energy = -503.622067

Quasi-harmonic Gibbs free energy = -503.620891

Cartesian Coordinates

C	-1.27222	-1.13461	0.40230
C	-0.62899	-0.15677	-0.41531
C	-2.57759	-0.98523	0.75760
H	-3.07004	-1.72636	1.36950
C	-1.37736	0.99199	-0.82495
C	-3.29605	0.14971	0.32627
H	-0.88867	1.73564	-1.43830
H	-4.33024	0.26667	0.61913
C	-2.68487	1.13383	-0.47274
H	-3.25543	1.99112	-0.79767
H	-0.71763	-2.00035	0.73137
C	0.76335	-0.30281	-0.84196
C	1.18040	-1.73200	-1.17903
H	1.18560	-2.39625	-0.31704
H	0.50453	-2.15091	-1.92243
H	2.18045	-1.72161	-1.60721
C	1.77548	0.36580	0.26712
C	1.68190	-0.34041	1.61049
H	2.41611	0.10081	2.28387
H	0.69533	-0.21430	2.05396
H	1.89277	-1.40666	1.53122
C	1.48315	1.85055	0.39504
H	2.22926	2.29771	1.05045
H	1.53661	2.34502	-0.57408
H	0.49947	2.02404	0.82896
O	3.05898	0.25961	-0.27324
H	3.43660	-0.59694	-0.06459
H	0.93025	0.33519	-1.71017

Sn1-like Transition State Conformer 1 (Dichloromethane)

Number of imaginary frequencies = 1

Imaginary frequency = -401.31

Electronic energy = -503.824459

Zero-point correction = 0.245192

Enthalpy = -503.566187

Temperature entropy product = 0.047957

Temperature quasi-harmonic entropy product = 0.047145

Gibbs free energy = -503.614144

Quasi-harmonic Gibbs free energy = -503.613332

Cartesian Coordinates

C	1.32924	1.15940	0.38457
C	0.62260	0.22967	-0.43094
C	2.63555	0.93720	0.71526
H	3.17634	1.64979	1.32057
C	1.29157	-0.95742	-0.84721
C	3.27932	-0.22746	0.26854
H	0.74878	-1.67733	-1.44414
H	4.30999	-0.40540	0.54289
C	2.59931	-1.17117	-0.51789
H	3.11240	-2.06052	-0.85327
H	0.82826	2.05180	0.72979
C	-0.74706	0.45262	-0.83044
C	-1.20981	1.89563	-0.93570
H	-1.30115	2.38622	0.03230
H	-0.49640	2.46328	-1.53173
H	-2.17297	1.94891	-1.43382
C	-1.75048	-0.44644	0.27964

C	-3.17277	-0.15606	-0.15781
H	-3.82327	-0.80839	0.42546
H	-3.45327	0.87371	0.04122
H	-3.31968	-0.37832	-1.21312
C	-1.46445	-0.01796	1.70505
H	-2.20835	-0.48015	2.35440
H	-0.47520	-0.33585	2.03328
H	-1.54143	1.06066	1.82189
O	-1.50882	-1.78225	0.05891
H	-0.81106	-2.10384	0.63436
H	-0.98058	-0.11461	-1.72984

Sn1-like Transition State Conformer 1 (Methanol)

Number of imaginary frequencies = 1

Imaginary frequency = -439.36

Electronic energy = -503.830825

Zero-point correction = 0.245044

Enthalpy = -503.572666

Temperature entropy product = 0.048375

Temperature quasi-harmonic entropy product = 0.047267

Gibbs free energy = -503.621041

Quasi-harmonic Gibbs free energy = -503.619932

Cartesian Coordinates

C	1.33905	1.16791	0.35657
C	0.62505	0.23642	-0.44519
C	2.64128	0.93164	0.69961
H	3.18714	1.64700	1.29729
C	1.28046	-0.96238	-0.84061
C	3.27163	-0.24711	0.27595

H	0.73219	-1.68864	-1.42329
H	4.29776	-0.43482	0.56078
C	2.58578	-1.18886	-0.50012
H	3.08598	-2.09191	-0.81803
H	0.85162	2.07439	0.68402
C	-0.73628	0.47112	-0.84940
C	-1.21646	1.90587	-0.93241
H	-1.29792	2.38582	0.04188
H	-0.51335	2.48494	-1.53048
H	-2.18584	1.95757	-1.41844
C	-1.74987	-0.45620	0.30719
C	-3.17362	-0.10960	-0.07799
H	-3.82601	-0.76561	0.50075
H	-3.41929	0.91807	0.17120
H	-3.36349	-0.27736	-1.13790
C	-1.37550	-0.05791	1.71507
H	-2.12408	-0.47270	2.39108
H	-0.40248	-0.45936	1.98749
H	-1.37138	1.02230	1.83848
O	-1.45545	-1.77556	0.11908
H	-1.96443	-2.14085	-0.60938
H	-0.99190	-0.09977	-1.74013

Sn1-like Transition State Conformer 2 (Dichloromethane)

Number of imaginary frequencies = 1

Imaginary frequency = -385.21

Electronic energy = -503.825303

Zero-point correction = 0.245272

Enthalpy = -503.566901

Temperature entropy product = 0.048387

Temperature quasi-harmonic entropy product = 0.047341

Gibbs free energy = -503.615288

Quasi-harmonic Gibbs free energy = -503.614242

Cartesian Coordinates

C	1.22923	1.05705	0.53640
C	0.62972	0.22314	-0.44331
C	2.53342	0.86684	0.90207
H	2.99021	1.50315	1.64607
C	1.39875	-0.83769	-0.99659
C	3.27985	-0.16687	0.31844
H	0.94236	-1.48782	-1.73055
H	4.30743	-0.31765	0.61963
C	2.70745	-1.01399	-0.63557
H	3.29576	-1.80417	-1.07867
H	0.64537	1.83645	1.00287
C	-0.72962	0.41523	-0.87594
C	-1.22518	1.84010	-1.01838
H	-1.18695	2.39800	-0.08493
H	-0.58940	2.35794	-1.73603
H	-2.24130	1.86714	-1.40091
C	-1.76188	-0.42220	0.33980
C	-1.17179	-1.78589	0.61625
H	-1.88924	-2.34389	1.21829
H	-1.00032	-2.33428	-0.30711
H	-0.24155	-1.70847	1.17315
C	-3.12591	-0.48372	-0.31789
H	-3.78125	-1.04095	0.35372
H	-3.56070	0.50100	-0.47397
H	-3.08268	-1.00832	-1.26903
O	-1.73104	0.32406	1.47724
H	-2.38416	1.02813	1.44776

H	-0.95947	-0.19070	-1.74984

Sn1-like Transition State Conformer 2 (Methanol)

Number of imaginary frequencies = 1

Imaginary frequency = -444.65

Electronic energy = -503.830847

Zero-point correction = 0.245129

Enthalpy = -503.572577

Temperature entropy product = 0.048383

Temperature quasi-harmonic entropy product = 0.047342

Gibbs free energy = -503.620960

Quasi-harmonic Gibbs free energy = -503.619919

Cartesian Coordinates

C	1.24676	1.08731	0.49507
C	0.62637	0.21835	-0.44165
C	2.55031	0.89596	0.85540
H	3.02560	1.55905	1.56345
C	1.37938	-0.87319	-0.96275
C	3.27937	-0.17369	0.30707
H	0.90565	-1.54610	-1.66413
H	4.30782	-0.32394	0.60520
C	2.68853	-1.05157	-0.60746
H	3.26345	-1.86597	-1.02302
H	0.67931	1.90223	0.92074
C	-0.73850	0.41242	-0.86061
C	-1.22079	1.84675	-0.99305
H	-1.34687	2.32946	-0.02588
H	-0.49651	2.41929	-1.57069
H	-2.16964	1.88046	-1.52049

C	-1.76016	-0.40774	0.32682
C	-1.30555	-1.84808	0.45040
H	-2.02556	-2.37735	1.07485
H	-1.27766	-2.33316	-0.52315
H	-0.32299	-1.92959	0.91295
C	-3.16043	-0.27852	-0.23704
H	-3.81633	-0.88761	0.38661
H	-3.51483	0.74732	-0.19624
H	-3.21425	-0.64643	-1.25879
O	-1.69453	0.29134	1.50062
H	-0.98753	-0.03944	2.06080
H	-0.96530	-0.18695	-1.74029

Sn1-like Transition State Conformer 3 (Dichloromethane)

Number of imaginary frequencies = 1

Imaginary frequency = -240.10

Electronic energy = -503.826499

Zero-point correction = 0.245705

Enthalpy = -503.567870

Temperature entropy product = 0.047780

Temperature quasi-harmonic entropy product = 0.046901

Gibbs free energy = -503.615650

Quasi-harmonic Gibbs free energy = -503.614770

Cartesian Coordinates

C	-1.28714	-1.13455	0.39359
C	-0.62953	-0.17244	-0.42435
C	-2.59487	-0.96493	0.74765
H	-3.09578	-1.70185	1.35820
C	-1.35734	0.98744	-0.82429

C	-3.29392	0.17552	0.31928
H	-0.86085	1.72876	-1.43479
H	-4.32679	0.30776	0.61073
C	-2.66761	1.14743	-0.47306
H	-3.22152	2.01545	-0.79911
H	-0.74960	-2.00972	0.72611
C	0.73996	-0.34587	-0.86427
C	1.19653	-1.76589	-1.15224
H	1.10276	-2.43161	-0.29718
H	0.59468	-2.17551	-1.96264
H	2.23838	-1.75772	-1.45978
C	1.79781	0.36803	0.29685
C	1.70024	-0.37887	1.60926
H	2.40606	0.07467	2.30485
H	0.70116	-0.30069	2.03193
H	1.96682	-1.42692	1.49628
C	1.46074	1.84146	0.42350
H	2.21473	2.30904	1.05669
H	1.46464	2.34258	-0.54478
H	0.48654	1.98378	0.88680
O	3.05947	0.16916	-0.21290
H	3.27468	0.84254	-0.86357
H	0.93902	0.30224	-1.71805

Sn1-like Transition State Conformer 3 (Methanol)

Number of imaginary frequencies = 1

Imaginary frequency = -339.55

Electronic energy = -503.832275

Zero-point correction = 0.245256

Enthalpy = -503.573928

Temperature entropy product = 0.048447

Temperature quasi-harmonic entropy product = 0.047292

Gibbs free energy = -503.622376

Quasi-harmonic Gibbs free energy = -503.621221

Cartesian Coordinates

C	-1.27785	-1.13571	0.40333
C	-0.62966	-0.18349	-0.43073
C	-2.58381	-0.96281	0.76682
H	-3.07770	-1.69254	1.39159
C	-1.36129	0.96767	-0.84135
C	-3.28737	0.16977	0.33002
H	-0.87208	1.70054	-1.46769
H	-4.31778	0.30470	0.62871
C	-2.67029	1.12994	-0.48044
H	-3.22778	1.99267	-0.81440
H	-0.73585	-2.00553	0.74354
C	0.73276	-0.35933	-0.87729
C	1.20583	-1.77777	-1.13308
H	1.17969	-2.41385	-0.25072
H	0.56523	-2.23290	-1.88757
H	2.22126	-1.76682	-1.52143
C	1.80033	0.39982	0.29181
C	1.64758	-0.28245	1.63470
H	2.37291	0.16052	2.31784
H	0.65115	-0.12514	2.04063
H	1.84272	-1.35237	1.57953
C	1.48430	1.87833	0.32285
H	2.22024	2.36362	0.96381
H	1.55136	2.31996	-0.66969
H	0.49409	2.05900	0.73399
O	3.04795	0.24178	-0.25011

H	3.43736	-0.59348	0.02145
H	0.94839	0.28350	-1.72851

Sn2-like Pre-reaction Complex Conformer 1 (Dichloromethane)

Number of imaginary frequencies = 0

Electronic energy = -619.146959

Zero-point correction = 0.285332

Enthalpy = -618.843691

Temperature entropy product = 0.061614

Temperature quasi-harmonic entropy product = 0.058003

Gibbs free energy = -618.905304

Quasi-harmonic Gibbs free energy = -618.901694

Cartesian Coordinates

C	0.80737	-0.34873	1.14200
C	0.18409	-0.29287	-0.10234
C	1.89277	-1.18352	1.36353
H	2.36169	-1.20505	2.33856
C	0.68634	-1.09908	-1.11977
C	2.38217	-1.98036	0.33893
H	0.22548	-1.06496	-2.09909
H	3.23288	-2.62698	0.50823
C	1.77316	-1.93473	-0.90725
H	2.14729	-2.54701	-1.71738
H	0.45054	0.27216	1.95262
C	-0.99225	0.62325	-0.36847
C	-0.82147	1.99077	0.29801
H	-0.97083	1.94546	1.37715
H	0.18443	2.37182	0.12321
H	-1.53058	2.71239	-0.10351

C	-2.36073	-0.05123	-0.05008
C	-3.51259	0.89785	-0.37719
H	-4.45459	0.35417	-0.30901
H	-3.55316	1.73732	0.31551
H	-3.41516	1.29686	-1.38964
C	-2.47116	-0.53331	1.38809
H	-3.45931	-0.96292	1.55237
H	-1.72482	-1.29720	1.59878
H	-2.33749	0.28853	2.09062
O	-2.49299	-1.23023	-0.84720
H	-2.50322	-0.97452	-1.77085
H	-1.01106	0.79783	-1.44975
O	2.96993	2.23960	0.36033
C	3.01067	1.66311	-0.86565
H	2.93190	0.57321	-0.68320
H	2.16570	1.93564	-1.50918
H	3.97441	1.81146	-1.36936

Sn2-like Pre-reaction Complex Conformer 1 (Methanol)

Number of imaginary frequencies = 0

Electronic energy = -619.148473

Zero-point correction = 0.285219

Enthalpy = -618.845278

Temperature entropy product = 0.061892

Temperature quasi-harmonic entropy product = 0.058107

Gibbs free energy = -618.907171

Quasi-harmonic Gibbs free energy = -618.903386

Cartesian Coordinates

C	0.80129	-0.35570	1.14384

C	0.18243	-0.29263	-0.10251
C	1.88824	-1.18932	1.36346
H	2.35347	-1.21689	2.34016
C	0.69043	-1.09059	-1.12382
C	2.38346	-1.97783	0.33505
H	0.23401	-1.04952	-2.10498
H	3.23560	-2.62299	0.50272
C	1.77872	-1.92513	-0.91307
H	2.15804	-2.52991	-1.72641
H	0.43924	0.25788	1.95783
C	-0.99425	0.62325	-0.36788
C	-0.82313	1.99042	0.29914
H	-0.96619	1.94408	1.37905
H	0.18085	2.37344	0.11759
H	-1.53561	2.71087	-0.09834
C	-2.36278	-0.05087	-0.04990
C	-3.51428	0.89452	-0.38842
H	-4.45634	0.35101	-0.31884
H	-3.55755	1.73911	0.29781
H	-3.41341	1.28521	-1.40358
C	-2.47928	-0.52344	1.39096
H	-3.46721	-0.95448	1.55340
H	-1.73179	-1.28340	1.61226
H	-2.35209	0.30369	2.08831
O	-2.49035	-1.23567	-0.84050
H	-2.48602	-0.98651	-1.76619
H	-1.01294	0.79861	-1.44878
O	2.98761	2.21946	0.37391
C	3.00787	1.67127	-0.86563
H	2.92219	0.57793	-0.70734
H	2.15823	1.96620	-1.49247
H	3.96726	1.82173	-1.37679

Sn2-like Pre-reaction Complex Conformer 2 (Dichloromethane)

Number of imaginary frequencies = 0

Electronic energy = -619.148692

Zero-point correction = 0.285363

Enthalpy = -618.845404

Temperature entropy product = 0.061759

Temperature quasi-harmonic entropy product = 0.058122

Gibbs free energy = -618.907163

Quasi-harmonic Gibbs free energy = -618.903525

Cartesian Coordinates

C	0.74884	-0.33151	1.11615
C	0.16815	-0.29254	-0.15049
C	1.82105	-1.17061	1.38654
H	2.25690	-1.17994	2.37689
C	0.70325	-1.11408	-1.14008
C	2.34103	-1.98347	0.39070
H	0.27346	-1.09352	-2.13420
H	3.18147	-2.63217	0.59834
C	1.77809	-1.95032	-0.87793
H	2.17882	-2.57414	-1.66618
H	0.37339	0.31268	1.90007
C	-1.00729	0.61123	-0.45820
C	-0.80005	2.01734	0.10830
H	-0.96882	2.03775	1.18505
H	0.21802	2.35801	-0.07847
H	-1.48335	2.73001	-0.35043
C	-2.36114	-0.02197	-0.00694
C	-2.58970	-1.36565	-0.69585

H	-3.55335	-1.77394	-0.39187
H	-2.58844	-1.25211	-1.78085
H	-1.81356	-2.08487	-0.43238
C	-3.52159	0.91446	-0.31571
H	-4.46567	0.41981	-0.08778
H	-3.46213	1.82246	0.28136
H	-3.52172	1.18995	-1.37066
O	-2.38599	-0.20385	1.40817
H	-1.73842	-0.87238	1.63939
H	-1.06839	0.69153	-1.54653
O	2.98942	2.21241	0.37568
C	3.06699	1.62582	-0.84375
H	2.96880	0.53854	-0.65591
H	2.24972	1.90377	-1.51987
H	4.05016	1.75800	-1.31324

Sn2-like Pre-reaction Complex Conformer 2 (Methanol)

Number of imaginary frequencies = 0

Electronic energy = -619.149856

Zero-point correction = 0.285295

Enthalpy = -618.846631

Temperature entropy product = 0.061563

Temperature quasi-harmonic entropy product = 0.058032

Gibbs free energy = -618.908194

Quasi-harmonic Gibbs free energy = -618.904663

Cartesian Coordinates

C	0.74511	-0.33588	1.11702
C	0.16688	-0.29217	-0.15063
C	1.81950	-1.17298	1.38546

H	2.25283	-1.18675	2.37692
C	0.70617	-1.10733	-1.14322
C	2.34415	-1.97904	0.38647
H	0.27865	-1.08281	-2.13817
H	3.18644	-2.62590	0.59233
C	1.78332	-1.94147	-0.88306
H	2.18778	-2.55970	-1.67377
H	0.36537	0.30200	1.90413
C	-1.00944	0.61094	-0.45735
C	-0.80138	2.01720	0.10834
H	-0.96093	2.03741	1.18655
H	0.21439	2.35960	-0.08779
H	-1.48941	2.72869	-0.34503
C	-2.36319	-0.02222	-0.00689
C	-2.58913	-1.36736	-0.69349
H	-3.55370	-1.77561	-0.39220
H	-2.58397	-1.25583	-1.77859
H	-1.81344	-2.08546	-0.42572
C	-3.52393	0.91259	-0.31931
H	-4.46819	0.41738	-0.09294
H	-3.46664	1.82221	0.27570
H	-3.52200	1.18619	-1.37466
O	-2.39035	-0.20215	1.40936
H	-1.74582	-0.87313	1.64224
H	-1.07097	0.69174	-1.54545
O	3.00017	2.19184	0.38927
C	3.06529	1.63386	-0.84437
H	2.94558	0.54426	-0.68308
H	2.25409	1.94492	-1.51299
H	4.05120	1.75657	-1.31046

Sn2-like Pre-reaction Complex Conformer 3 (Dichloromethane)

Number of imaginary frequencies = 0

Electronic energy = -619.147704

Zero-point correction = 0.285404

Enthalpy = -618.844314

Temperature entropy product = 0.062975

Temperature quasi-harmonic entropy product = 0.058567

Gibbs free energy = -618.907289

Quasi-harmonic Gibbs free energy = -618.902881

Cartesian Coordinates

C	0.76060	-0.34479	1.13878
C	0.17469	-0.30352	-0.12457
C	1.83796	-1.17772	1.40241
H	2.27713	-1.18798	2.39132
C	0.71072	-1.11899	-1.11823
C	2.35792	-1.98675	0.40261
H	0.27906	-1.09566	-2.11136
H	3.20192	-2.63230	0.60572
C	1.78938	-1.95321	-0.86281
H	2.18864	-2.57339	-1.65467
H	0.38218	0.28686	1.93105
C	-0.99692	0.60384	-0.44098
C	-0.78938	2.01506	0.11257
H	-0.81145	2.04631	1.20179
H	0.17983	2.40138	-0.20126
H	-1.55221	2.69846	-0.26040
C	-2.37669	-0.00985	-0.04892
C	-2.55504	-0.18611	1.45574

H	-3.54913	-0.58591	1.65539
H	-1.82007	-0.87942	1.86227
H	-2.45824	0.76188	1.98734
C	-2.59271	-1.33920	-0.75768
H	-3.59978	-1.70212	-0.55355
H	-2.47953	-1.22203	-1.83617
H	-1.87912	-2.08638	-0.41483
O	-3.40779	0.84532	-0.55164
H	-3.44258	1.63635	-0.01324
H	-1.04212	0.69145	-1.52940
O	2.95272	2.21367	0.36429
C	3.08191	1.60781	-0.84120
H	2.95740	0.52499	-0.64338
H	2.30453	1.88814	-1.56189
H	4.08946	1.71802	-1.26229

Sn2-like Pre-reaction Complex Conformer 3 (Methanol)

Number of imaginary frequencies = 0

Electronic energy = -619.148929

Zero-point correction = 0.285509

Enthalpy = -618.845487

Temperature entropy product = 0.062362

Temperature quasi-harmonic entropy product = 0.058201

Gibbs free energy = -618.907849

Quasi-harmonic Gibbs free energy = -618.903688

Cartesian Coordinates

C	0.74932	-0.33226	1.12853
C	0.16397	-0.30062	-0.13556
C	1.82919	-1.16028	1.39803

H	2.26706	-1.16380	2.38758
C	0.70386	-1.11979	-1.12414
C	2.35289	-1.97329	0.40334
H	0.27311	-1.10395	-2.11779
H	3.19903	-2.61463	0.61079
C	1.78565	-1.94862	-0.86289
H	2.18815	-2.57140	-1.65103
H	0.36796	0.30212	1.91728
C	-1.01140	0.59978	-0.45834
C	-0.79741	2.02163	0.06417
H	-0.78568	2.07132	1.15275
H	0.15807	2.40819	-0.28924
H	-1.57561	2.69438	-0.29611
C	-2.38714	-0.00584	-0.04096
C	-2.55774	-0.14083	1.46860
H	-3.55035	-0.53597	1.68449
H	-1.82032	-0.82241	1.89007
H	-2.45882	0.82159	1.97311
C	-2.60343	-1.35419	-0.71286
H	-3.60828	-1.71477	-0.49377
H	-2.49500	-1.26619	-1.79469
H	-1.88610	-2.09002	-0.35358
O	-3.42307	0.83410	-0.56107
H	-3.46398	1.63465	-0.03711
H	-1.06736	0.66513	-1.54775
O	3.02201	2.14157	0.40148
C	3.11496	1.60083	-0.83807
H	2.92177	0.51809	-0.70454
H	2.35974	1.97302	-1.54017
H	4.12875	1.67013	-1.25264

Sn2-like Transition State Conformer 1 (Dichloromethane)

Number of imaginary frequencies = 1

Imaginary frequency = -764.29

Electronic energy = -619.077228

Zero-point correction = 0.285131

Enthalpy = -618.775113

Temperature entropy product = 0.057121

Temperature quasi-harmonic entropy product = 0.055468

Gibbs free energy = -618.832234

Quasi-harmonic Gibbs free energy = -618.830581

Cartesian Coordinates

C	1.48718	0.11761	1.23430
C	0.71358	0.26021	0.08316
C	2.85202	-0.11433	1.14972
H	3.43357	-0.22281	2.05603
C	1.34998	0.17553	-1.15614
C	3.47001	-0.20689	-0.08787
H	0.76609	0.30022	-2.05766
H	4.53459	-0.38845	-0.15459
C	2.71075	-0.05809	-1.24215
H	3.18427	-0.11992	-2.21344
H	1.02333	0.18177	2.20934
C	-0.73339	0.49065	0.15836
C	-1.39987	0.76388	1.47715
H	-1.35658	-0.09112	2.15277
H	-0.90661	1.59906	1.97316
H	-2.44555	1.02536	1.33700
C	-1.37468	-1.54809	-0.20786

C	-2.85683	-1.45226	0.00320
H	-3.31355	-2.40551	-0.27909
H	-3.10026	-1.25938	1.04419
H	-3.30100	-0.66741	-0.61177
C	-0.59823	-2.41560	0.72702
H	-0.98821	-3.43635	0.66650
H	0.45489	-2.43171	0.45615
H	-0.69670	-2.08204	1.75782
O	-0.99962	-1.77347	-1.49444
H	-1.63793	-1.39293	-2.10169
H	-1.27416	0.61783	-0.76279
O	-0.47597	2.49892	-0.32292
C	-1.69326	3.14034	-0.40580
H	-2.40431	2.63707	-1.08262
H	-2.19394	3.26703	0.56757
H	-1.53690	4.14803	-0.81645

Sn2-like Transition State Conformer 1 (Methanol)

Number of imaginary frequencies = 1

Imaginary frequency = -732.27

Electronic energy = -619.079767

Zero-point correction = 0.285152

Enthalpy = -618.777622

Temperature entropy product = 0.057179

Temperature quasi-harmonic entropy product = 0.055499

Gibbs free energy = -618.834801

Quasi-harmonic Gibbs free energy = -618.833121

Cartesian Coordinates

C	1.48776	0.11939	1.23491

C	0.71292	0.25958	0.08386
C	2.85328	-0.10947	1.14989
H	3.43515	-0.21698	2.05609
C	1.34935	0.17429	-1.15599
C	3.47119	-0.20155	-0.08800
H	0.76419	0.29363	-2.05755
H	4.53603	-0.38138	-0.15496
C	2.71085	-0.05620	-1.24233
H	3.18396	-0.11933	-2.21377
H	1.02398	0.18144	2.21014
C	-0.73398	0.48655	0.15960
C	-1.40115	0.75985	1.47767
H	-1.35612	-0.09430	2.15418
H	-0.91036	1.59621	1.97429
H	-2.44729	1.01844	1.33655
C	-1.37310	-1.55353	-0.20968
C	-2.85407	-1.45692	0.00587
H	-3.31044	-2.40929	-0.27950
H	-3.09390	-1.26952	1.04856
H	-3.29969	-0.66993	-0.60464
C	-0.59270	-2.41793	0.72466
H	-0.98359	-3.43841	0.66710
H	0.45960	-2.43505	0.45063
H	-0.68866	-2.08285	1.75510
O	-0.99857	-1.77345	-1.49558
H	-1.63950	-1.39614	-2.10272
H	-1.27423	0.61655	-0.76134
O	-0.47841	2.50840	-0.32320
C	-1.70029	3.14228	-0.40640
H	-2.40886	2.63142	-1.07974
H	-2.20057	3.26713	0.56742
H	-1.55424	4.15072	-0.81991

Sn2-like Transition State Conformer 2 (Dichloromethane)

Number of imaginary frequencies = 1

Imaginary frequency = -763.48

Electronic energy = -619.080327

Zero-point correction = 0.285575

Enthalpy = -618.777883

Temperature entropy product = 0.056938

Temperature quasi-harmonic entropy product = 0.055290

Gibbs free energy = -618.834821

Quasi-harmonic Gibbs free energy = -618.833173

Cartesian Coordinates

C	1.39964	-0.11299	1.23275
C	0.69504	0.28080	0.09544
C	2.76988	-0.33498	1.18001
H	3.29637	-0.64091	2.07466
C	1.40713	0.46071	-1.09330
C	3.46088	-0.16091	-0.00790
H	0.87643	0.78653	-1.97773
H	4.52844	-0.33148	-0.04884
C	2.77113	0.24244	-1.14590
H	3.30263	0.39055	-2.07693
H	0.87880	-0.24751	2.17114
C	-0.75920	0.48933	0.13214
C	-1.46194	0.66631	1.44809
H	-1.44188	-0.24452	2.04751
H	-0.97375	1.45538	2.01902
H	-2.50082	0.94931	1.29839
C	-1.32901	-1.56727	-0.23179

C	-0.72031	-1.89266	-1.56234
H	-1.07139	-2.87992	-1.87615
H	-1.02235	-1.17019	-2.31876
H	0.36741	-1.91499	-1.51921
C	-2.82371	-1.50309	-0.19022
H	-3.22940	-2.47767	-0.47722
H	-3.18595	-1.27048	0.80796
H	-3.20075	-0.76176	-0.89253
O	-0.84129	-2.27099	0.82074
H	0.10997	-2.37998	0.72972
H	-1.27713	0.63935	-0.79987
O	-0.53274	2.51070	-0.23090
C	-1.76205	3.12925	-0.32244
H	-2.44420	2.63518	-1.03444
H	-2.28921	3.21044	0.64129
H	-1.61576	4.15326	-0.69396

Sn2-like Transition State Conformer 2 (Methanol)

Number of imaginary frequencies = 1

Imaginary frequency = -731.17

Electronic energy = -619.082236

Zero-point correction = 0.285575

Enthalpy = -618.779804

Temperature entropy product = 0.056996

Temperature quasi-harmonic entropy product = 0.055285

Gibbs free energy = -618.836800

Quasi-harmonic Gibbs free energy = -618.835089

Cartesian Coordinates

C	1.40353	-0.10584	1.23418

C	0.69748	0.27712	0.09386
C	2.77401	-0.32640	1.18236
H	3.30125	-0.62546	2.07886
C	1.40838	0.44561	-1.09763
C	3.46408	-0.16186	-0.00767
H	0.87625	0.75807	-1.98609
H	4.53167	-0.33240	-0.04804
C	2.77283	0.22898	-1.14929
H	3.30304	0.36667	-2.08266
H	0.88327	-0.23565	2.17353
C	-0.75606	0.48628	0.13048
C	-1.45840	0.67068	1.44534
H	-1.44052	-0.23775	2.04843
H	-0.96859	1.46012	2.01442
H	-2.49641	0.95575	1.29422
C	-1.33491	-1.56965	-0.22783
C	-0.72358	-1.90154	-1.55499
H	-1.08071	-2.88720	-1.86668
H	-1.01882	-1.17942	-2.31420
H	0.36371	-1.93181	-1.50754
C	-2.82895	-1.49215	-0.18983
H	-3.24055	-2.46376	-0.47821
H	-3.19187	-1.25701	0.80747
H	-3.19829	-0.74818	-0.89320
O	-0.85401	-2.26899	0.82893
H	0.09257	-2.40740	0.73023
H	-1.27282	0.63865	-0.80171
O	-0.52765	2.51883	-0.23862
C	-1.75904	3.13491	-0.32554
H	-2.44592	2.63241	-1.02680
H	-2.27897	3.22202	0.64170
H	-1.62123	4.15682	-0.70690

Sn2-like Transition State Conformer 3 (Dichloromethane)

Number of imaginary frequencies = 1

Imaginary frequency = -761.41

Electronic energy = -619.077843

Zero-point correction = 0.285463

Enthalpy = -618.775455

Temperature entropy product = 0.056967

Temperature quasi-harmonic entropy product = 0.055366

Gibbs free energy = -618.832421

Quasi-harmonic Gibbs free energy = -618.830820

Cartesian Coordinates

C	-1.47404	-0.01544	-1.23719
C	-0.72973	0.23513	-0.08495
C	-2.83243	-0.28494	-1.16266
H	-3.38962	-0.47918	-2.06991
C	-1.39470	0.23132	1.14304
C	-3.47576	-0.30498	0.06531
H	-0.84007	0.46298	2.04261
H	-4.53527	-0.51603	0.12365
C	-2.74908	-0.04012	1.21962
H	-3.24329	-0.03692	2.18245
H	-0.99181	-0.00643	-2.20534
C	0.71173	0.50678	-0.14845
C	1.38711	0.75854	-1.46618
H	1.33846	-0.10230	-2.13585
H	0.90183	1.58833	-1.97841
H	2.43367	1.01281	-1.31981
C	1.49546	-1.48746	0.21476

C 0.72368 -2.41569 -0.67097

H 1.14957 -3.41958 -0.58012

H -0.32092 -2.45569 -0.37445

H 0.77346 -2.12486 -1.72106

C 1.32176 -1.66548 1.68851

H 1.70367 -2.64937 1.97572

H 1.87729 -0.91222 2.24553

H 0.27157 -1.61816 1.96399

O 2.81180 -1.33988 -0.09499

H 2.94616 -1.42240 -1.04158

H 1.24206 0.67397 0.77155

O 0.38215 2.51872 0.26986

C 1.57761 3.19997 0.36034

H 2.27943 2.75247 1.08338

H 2.10587 3.29470 -0.60203

H 1.37681 4.22140 0.71364

Sn2-like Transition State Conformer 3 (Methanol)

Number of imaginary frequencies = 1

Imaginary frequency = -730.78

Electronic energy = -619.080056

Zero-point correction = 0.285379

Enthalpy = -618.777737

Temperature entropy product = 0.056962

Temperature quasi-harmonic entropy product = 0.055405

Gibbs free energy = -618.834699

Quasi-harmonic Gibbs free energy = -618.833142

Cartesian Coordinates

C -1.47528 -0.01214 -1.23753

C	-0.72999	0.23257	-0.08418
C	-2.83405	-0.28049	-1.16357
H	-3.39153	-0.47103	-2.07140
C	-1.39453	0.22218	1.14449
C	-3.47699	-0.30571	0.06476
H	-0.83910	0.44531	2.04580
H	-4.53650	-0.51689	0.12260
C	-2.74932	-0.04822	1.22034
H	-3.24263	-0.05164	2.18362
H	-0.99317	-0.00105	-2.20571
C	0.71100	0.50352	-0.14746
C	1.38697	0.75948	-1.46371
H	1.33595	-0.09815	-2.13699
H	0.90450	1.59257	-1.97319
H	2.43421	1.00998	-1.31643
C	1.49908	-1.49060	0.21315
C	0.72436	-2.41555	-0.67284
H	1.15090	-3.41926	-0.58433
H	-0.31914	-2.45658	-0.37287
H	0.77255	-2.12335	-1.72240
C	1.32730	-1.66732	1.68712
H	1.71042	-2.65118	1.97258
H	1.88304	-0.91372	2.24335
H	0.27763	-1.62191	1.96440
O	2.81199	-1.33809	-0.09987
H	2.94749	-1.43103	-1.04566
H	1.23901	0.67481	0.77314
O	0.37785	2.52826	0.27563
C	1.57560	3.20678	0.36062
H	2.28038	2.75541	1.07841
H	2.09926	3.30008	-0.60446
H	1.38316	4.22936	0.71632

CCCNCOC Cation Fragment (Dichloromethane)

Number of imaginary frequencies = 0

Electronic energy = -326.393800

Zero-point correction = 0.148643

Enthalpy = -326.235808

Temperature entropy product = 0.039152

Temperature quasi-harmonic entropy product = 0.038568

Gibbs free energy = -326.274959

Quasi-harmonic Gibbs free energy = -326.274376

Cartesian Coordinates

C	2.30617	-1.04758	-0.00012
C	1.17019	-0.10718	0.00004
C	1.45030	1.34168	0.00022
N	-0.03630	-0.59760	-0.00001
C	-1.30485	0.11962	-0.00006
O	-1.32232	1.30913	-0.00028
C	-2.47722	-0.80019	0.00016
H	2.92510	-0.83153	0.87328
H	2.01246	-2.09292	-0.00026
H	2.92504	-0.83126	-0.87349
H	0.99287	1.81043	-0.87143
H	2.52151	1.51286	0.00046
H	0.99251	1.81035	0.87171
H	-0.11940	-1.60671	-0.00009
H	-2.44773	-1.44224	0.88182
H	-3.38956	-0.21423	-0.00000
H	-2.44771	-1.44272	-0.88115

CCCNCOC Cation Fragment (Methanol)

Number of imaginary frequencies = 0

Electronic energy = -326.401315

Zero-point correction = 0.148418

Enthalpy = -326.243470

Temperature entropy product = 0.039342

Temperature quasi-harmonic entropy product = 0.038775

Gibbs free energy = -326.282812

Quasi-harmonic Gibbs free energy = -326.282244

Cartesian Coordinates

C	2.30475	-1.04793	0.00021
C	1.16968	-0.10712	-0.00009
C	1.45070	1.34141	-0.00034
N	-0.03696	-0.59667	-0.00005
C	-1.30307	0.11899	0.00006
O	-1.32304	1.30949	0.00043
C	-2.47587	-0.80018	-0.00021
H	2.92306	-0.83066	0.87364
H	2.00978	-2.09276	0.00066
H	2.92295	-0.83138	-0.87347
H	0.99296	1.80944	-0.87198
H	2.52208	1.51088	-0.00120
H	0.99448	1.80921	0.87225
H	-0.11982	-1.60574	0.00003
H	-2.44583	-1.44252	0.88111
H	-3.38820	-0.21418	-0.00035
H	-2.44552	-1.44249	-0.88154

CCCNCOC Radical Fragment (Dichloromethane)

Number of imaginary frequencies = 0

Electronic energy = -326.536792

Zero-point correction = 0.145472

Enthalpy = -326.381148

Temperature entropy product = 0.042383

Temperature quasi-harmonic entropy product = 0.041208

Gibbs free energy = -326.423531

Quasi-harmonic Gibbs free energy = -326.422356

Cartesian Coordinates

C	2.29150	-1.09072	-0.06725
C	1.21528	-0.06228	-0.14006
C	1.51778	1.35686	0.19168
N	-0.07715	-0.56556	-0.00936
C	-1.27256	0.08895	-0.04871
O	-1.38003	1.29483	-0.21907
C	-2.48786	-0.78831	0.13971
H	3.21154	-0.71098	-0.51025
H	2.52378	-1.36835	0.97065
H	2.01745	-2.00604	-0.59589
H	0.90195	2.05698	-0.36737
H	2.56815	1.55905	-0.01405
H	1.34264	1.56434	1.25628
H	-0.14217	-1.56729	0.05911
H	-2.26039	-1.85148	0.18864
H	-2.98928	-0.49462	1.06184
H	-3.17827	-0.60838	-0.68307

CCCNCOC Radical Fragment (Methanol)

Number of imaginary frequencies = 0

Electronic energy = -326.538163

Zero-point correction = 0.145347

Enthalpy = -326.382579

Temperature entropy product = 0.042771

Temperature quasi-harmonic entropy product = 0.041398

Gibbs free energy = -326.425350

Quasi-harmonic Gibbs free energy = -326.423977

Cartesian Coordinates

C	2.29066	-1.09015	-0.07567
C	1.21435	-0.06115	-0.13928
C	1.51916	1.35508	0.20382
N	-0.07777	-0.56476	-0.00724
C	-1.27245	0.08780	-0.05159
O	-1.38029	1.29333	-0.23531
C	-2.48745	-0.78636	0.14777
H	3.20858	-0.70872	-0.52160
H	2.52729	-1.37065	0.96030
H	2.01315	-2.00356	-0.60535
H	0.90724	2.06187	-0.35129
H	2.57048	1.55640	0.00249
H	1.34236	1.55360	1.26987
H	-0.14220	-1.56626	0.06842
H	-2.25942	-1.84858	0.20837
H	-2.98723	-0.48280	1.06768
H	-3.17914	-0.61591	-0.67602

CCCO Cation Fragment (Dichloromethane)

Number of imaginary frequencies = 0

Electronic energy = -193.583905

Zero-point correction = 0.097661

Enthalpy = -193.480032

Temperature entropy product = 0.030852

Temperature quasi-harmonic entropy product = 0.030755

Gibbs free energy = -193.510883

Quasi-harmonic Gibbs free energy = -193.510786

Cartesian Coordinates

C	1.19847	-0.77014	-0.00536
C	-0.00461	0.06804	-0.00210
C	-1.35007	-0.50845	0.00471
O	0.06622	1.33291	0.00310
H	1.27253	-1.22067	0.98979
H	2.10714	-0.21413	-0.21825
H	1.06961	-1.59329	-0.70694
H	-1.39556	-1.31635	0.73513
H	-1.50616	-0.97251	-0.97430
H	-2.11302	0.24079	0.18529
H	0.97296	1.67619	-0.01905

CCCO Cation Fragment (Methanol)

Number of imaginary frequencies = 0

Electronic energy = -193.591625

Zero-point correction = 0.097281

Enthalpy = -193.487991

Temperature entropy product = 0.031391

Temperature quasi-harmonic entropy product = 0.031095

Gibbs free energy = -193.519382

Quasi-harmonic Gibbs free energy = -193.519085

Cartesian Coordinates

C	1.20080	-0.76725	-0.00687
C	-0.00402	0.06729	-0.00164
C	-1.34663	-0.51477	0.00678
O	0.06005	1.33252	0.00433
H	1.32000	-1.13743	1.01756
H	2.09675	-0.22479	-0.29581
H	1.04499	-1.64057	-0.63752
H	-1.36749	-1.39397	0.64842
H	-1.53676	-0.86901	-1.01250
H	-2.10277	0.21202	0.28376
H	0.96399	1.68194	-0.02815

CCCO Radical Fragment (Dichloromethane)

Number of imaginary frequencies = 0

Electronic energy = -193.726768

Zero-point correction = 0.094728

Enthalpy = -193.625442

Temperature entropy product = 0.031923

Temperature quasi-harmonic entropy product = 0.031921

Gibbs free energy = -193.657365

Quasi-harmonic Gibbs free energy = -193.657363

Cartesian Coordinates

C	1.20979	-0.78496	0.03129
C	-0.00966	0.03017	-0.22428
C	-1.36584	-0.52043	0.02706
O	0.07284	1.37513	0.05323
H	1.41260	-0.88729	1.10790
H	2.09587	-0.33661	-0.42549
H	1.09334	-1.78792	-0.37782
H	-1.45542	-1.52293	-0.39014
H	-2.13451	0.11147	-0.42024
H	-1.58365	-0.58927	1.10323
H	0.98335	1.66282	-0.02774

CCCO Radical Fragment (Methanol)

Number of imaginary frequencies = 0

Electronic energy = -193.727386

Zero-point correction = 0.094724

Enthalpy = -193.626074

Temperature entropy product = 0.031897

Temperature quasi-harmonic entropy product = 0.031896

Gibbs free energy = -193.657972

Quasi-harmonic Gibbs free energy = -193.657971

Cartesian Coordinates

C	1.20931	-0.78574	0.03116
C	-0.00952	0.03020	-0.22483
C	-1.36599	-0.51988	0.02728
O	0.07349	1.37555	0.05269
H	1.41154	-0.88705	1.10789
H	2.09591	-0.33876	-0.42569
H	1.09115	-1.78901	-0.37670

H	-1.45605	-1.52240	-0.38966
H	-2.13539	0.11180	-0.41935
H	-1.58275	-0.58903	1.10366
H	0.98485	1.66252	-0.02329

CCC Cation Fragment (Dichloromethane)

Number of imaginary frequencies = 0

Electronic energy = -118.298527

Zero-point correction = 0.089164

Enthalpy = -118.203690

Temperature entropy product = 0.028361

Temperature quasi-harmonic entropy product = 0.028360

Gibbs free energy = -118.232051

Quasi-harmonic Gibbs free energy = -118.232050

Cartesian Coordinates

C	-1.27086	-0.19680	-0.02216
C	0.00000	0.45727	-0.00003
C	1.27086	-0.19680	0.02216
H	-1.58490	-0.05011	1.03551
H	-2.02073	0.34951	-0.59305
H	-1.23692	-1.26301	-0.22125
H	-0.00001	1.54512	-0.00002
H	1.58511	-0.04995	-1.03542
H	2.02054	0.34940	0.59341
H	1.23687	-1.26304	0.22106

CCC Cation Fragment (Methanol)

Number of imaginary frequencies = 0

Electronic energy = -118.306186

Zero-point correction = 0.089077

Enthalpy = -118.211393

Temperature entropy product = 0.028486

Temperature quasi-harmonic entropy product = 0.028478

Gibbs free energy = -118.239879

Quasi-harmonic Gibbs free energy = -118.239871

Cartesian Coordinates

C	-1.27068	-0.19711	-0.02182
C	0.00002	0.45713	-0.00003
C	1.27070	-0.19708	0.02184
H	-1.58778	-0.04528	1.03378
H	-2.01759	0.34785	-0.59779
H	-1.23545	-1.26366	-0.21755
H	-0.00003	1.54466	-0.00003
H	1.58759	-0.04567	-1.03388
H	2.01758	0.34803	0.59770
H	1.23547	-1.26359	0.21784

CCC Radical Fragment (Dichloromethane)

Number of imaginary frequencies = 0

Electronic energy = -118.483990

Zero-point correction = 0.088122

Enthalpy = -118.389722

Temperature entropy product = 0.030385

Temperature quasi-harmonic entropy product = 0.030214

Gibbs free energy = -118.420107

Quasi-harmonic Gibbs free energy = -118.419936

Cartesian Coordinates

C	1.28943	-0.19822	0.00278
C	0.00000	0.53650	-0.04600
C	-1.28943	-0.19822	0.00278
H	1.30075	-1.03019	-0.70855
H	2.13897	0.44890	-0.21434
H	1.46470	-0.64323	0.99301
H	-0.00000	1.60867	0.10239
H	-1.46469	-0.64325	0.99301
H	-2.13897	0.44890	-0.21432
H	-1.30076	-1.03017	-0.70857

CCC Radical Fragment (Methanol)

Number of imaginary frequencies = 0

Electronic energy = -118.484069

Zero-point correction = 0.088096

Enthalpy = -118.389832

Temperature entropy product = 0.030363

Temperature quasi-harmonic entropy product = 0.030201

Gibbs free energy = -118.420195

Quasi-harmonic Gibbs free energy = -118.420033

Cartesian Coordinates

C	1.28941	-0.19824	0.00282
C	-0.00000	0.53663	-0.04685
C	-1.28941	-0.19824	0.00282
H	1.30052	-1.03124	-0.70727
H	2.13911	0.44853	-0.21482

H	1.46429	-0.64197	0.99371
H	0.00001	1.60853	0.10407
H	-1.46430	-0.64195	0.99371
H	-2.13911	0.44852	-0.21486
H	-1.30049	-1.03127	-0.70724

CCCc1ccccc1 Cation Fragment (Dichloromethane)

Number of imaginary frequencies = 0

Electronic energy = -349.383819

Zero-point correction = 0.173957

Enthalpy = -349.200212

Temperature entropy product = 0.040534

Temperature quasi-harmonic entropy product = 0.039729

Gibbs free energy = -349.240746

Quasi-harmonic Gibbs free energy = -349.239941

Cartesian Coordinates

C	2.38111	1.23572	0.08379
C	1.57371	0.00790	-0.00635
C	0.16325	0.00922	-0.00731
C	-0.55728	-1.21087	0.03002
C	-1.92984	-1.21199	0.04805
C	-2.62093	-0.00382	0.00527
C	-1.94165	1.20968	-0.04412
C	-0.56800	1.22215	-0.03929
H	3.23456	1.05531	0.73923
H	2.81066	1.40565	-0.91090
H	1.85073	2.12468	0.39912
H	-0.03223	-2.15305	0.06566
H	-2.47147	-2.14543	0.09050

H	-3.70271	-0.00879	0.01011
H	-2.49216	2.13809	-0.08174
H	-0.05526	2.17024	-0.08092
C	2.35744	-1.24221	-0.08045
H	2.46585	-1.60850	0.94811
H	1.88518	-2.02529	-0.66407
H	3.35996	-1.04768	-0.45283

CCCc1ccccc1 Cation Fragment (Methanol)

Number of imaginary frequencies = 0

Electronic energy = -349.389782

Zero-point correction = 0.174022

Enthalpy = -349.206107

Temperature entropy product = 0.040626

Temperature quasi-harmonic entropy product = 0.039774

Gibbs free energy = -349.246733

Quasi-harmonic Gibbs free energy = -349.245881

Cartesian Coordinates

C	-2.36877	1.23842	0.09132
C	-1.57428	0.00008	-0.00006
C	-0.16304	0.00010	-0.00009
C	0.56203	-1.21611	0.03817
C	1.93545	-1.21040	0.05122
C	2.62083	-0.00004	0.00006
C	1.93558	1.21037	-0.05118
C	0.56215	1.21623	-0.03829
H	-2.62926	1.51582	-0.93817
H	-3.31216	1.04177	0.59801
H	-1.85943	2.07466	0.55467

H	0.04296	-2.16098	0.08034
H	2.48112	-2.14139	0.09583
H	3.70261	-0.00010	0.00012
H	2.48135	2.14132	-0.09573
H	0.04321	2.16116	-0.08052
C	-2.36853	-1.23848	-0.09126
H	-1.85976	-2.07365	-0.55726
H	-2.62581	-1.51783	0.93852
H	-3.31336	-1.04174	-0.59515

CCCc1ccccc1 Radical Fragment (Dichloromethane)

Number of imaginary frequencies = 0

Electronic energy = -349.558086

Zero-point correction = 0.171818

Enthalpy = -349.376387

Temperature entropy product = 0.041823

Temperature quasi-harmonic entropy product = 0.040971

Gibbs free energy = -349.418210

Quasi-harmonic Gibbs free energy = -349.417358

Cartesian Coordinates

C	2.36701	-1.28928	-0.00004
C	1.60851	0.00000	-0.00000
C	0.18568	0.00000	-0.00000
C	-0.55791	1.20538	-0.00002
C	-1.93831	1.19968	-0.00002
C	-2.64351	-0.00000	-0.00000
C	-1.93831	-1.19968	0.00002
C	-0.55791	-1.20538	0.00002
H	2.12955	-1.90079	-0.87632

H	3.44098	-1.11507	-0.00016
H	2.12975	-1.90072	0.87635
H	-0.03990	2.15431	-0.00004
H	-2.47456	2.14013	-0.00004
H	-3.72515	-0.00000	-0.00000
H	-2.47455	-2.14013	0.00005
H	-0.03990	-2.15431	0.00005
C	2.36700	1.28928	0.00004
H	2.12971	1.90075	-0.87631
H	2.12960	1.90076	0.87635
H	3.44098	1.11506	0.00011

CCCc1ccccc1 Radical Fragment (Methanol)

Number of imaginary frequencies = 0

Electronic energy = -349.558574

Zero-point correction = 0.171845

Enthalpy = -349.376885

Temperature entropy product = 0.041490

Temperature quasi-harmonic entropy product = 0.040826

Gibbs free energy = -349.418375

Quasi-harmonic Gibbs free energy = -349.417711

Cartesian Coordinates

C	-2.36712	1.28936	-0.00004
C	-1.60875	0.00000	-0.00004
C	-0.18586	0.00000	-0.00000
C	0.55787	-1.20559	0.00001
C	1.93845	-1.19985	0.00005
C	2.64380	0.00000	0.00007
C	1.93845	1.19985	0.00005

C	0.55787	1.20559	0.00002
H	-2.12954	1.90095	-0.87623
H	-3.44104	1.11516	-0.00004
H	-2.12953	1.90096	0.87614
H	0.03996	-2.15459	0.00000
H	2.47466	-2.14033	0.00006
H	3.72545	0.00000	0.00009
H	2.47466	2.14033	0.00006
H	0.03996	2.15459	0.00000
C	-2.36712	-1.28936	-0.00008
H	-2.12948	-1.90095	-0.87625
H	-2.12958	-1.90095	0.87612
H	-3.44104	-1.11516	-0.00014

CCO Cation Fragment (Dichloromethane)

Number of imaginary frequencies = 0

Electronic energy = -193.583905

Zero-point correction = 0.097661

Enthalpy = -193.480032

Temperature entropy product = 0.030852

Temperature quasi-harmonic entropy product = 0.030755

Gibbs free energy = -193.510883

Quasi-harmonic Gibbs free energy = -193.510786

Cartesian Coordinates

C	1.20833	-0.16036	-0.00001
C	-0.11233	0.42456	-0.00001
O	-1.12935	-0.31133	-0.00000
H	1.19057	-1.24454	-0.00010
H	1.74487	0.23758	-0.86810

H	1.74475	0.23739	0.86826
H	-0.24925	1.50465	0.00000
H	-1.97219	0.17036	0.00002

CCO Cation Fragment (Methanol)

Number of imaginary frequencies = 0

Electronic energy = -193.591625

Zero-point correction = 0.097281

Enthalpy = -193.487991

Temperature entropy product = 0.031391

Temperature quasi-harmonic entropy product = 0.031095

Gibbs free energy = -193.519382

Quasi-harmonic Gibbs free energy = -193.519085

Cartesian Coordinates

C	-1.20880	-0.16005	-0.00001
C	0.11254	0.42391	-0.00001
O	1.12955	-0.31163	-0.00000
H	-1.19205	-1.24417	-0.00032
H	-1.74365	0.23835	0.86867
H	-1.74398	0.23889	-0.86822
H	0.25034	1.50340	-0.00001
H	1.97051	0.17341	0.00002

CCO Radical Fragment (Dichloromethane)

Number of imaginary frequencies = 0

Electronic energy = -193.726768

Zero-point correction = 0.094728

Enthalpy = -193.625442

Temperature entropy product = 0.031923

Temperature quasi-harmonic entropy product = 0.031921

Gibbs free energy = -193.657365

Quasi-harmonic Gibbs free energy = -193.657363

Cartesian Coordinates

C	-1.22656	-0.16166	0.01276
C	0.09228	0.49996	-0.09900
O	1.16608	-0.33894	0.01646
H	-1.31398	-0.98785	-0.69711
H	-1.39863	-0.57755	1.01496
H	-2.02415	0.55143	-0.19160
H	0.23897	1.52255	0.23140
H	1.97480	0.17315	0.02815

CCO Radical Fragment (Methanol)

Number of imaginary frequencies = 0

Electronic energy = -193.727386

Zero-point correction = 0.094724

Enthalpy = -193.626074

Temperature entropy product = 0.031897

Temperature quasi-harmonic entropy product = 0.031896

Gibbs free energy = -193.657972

Quasi-harmonic Gibbs free energy = -193.657971

Cartesian Coordinates

C	-1.22671	-0.16154	0.01311
C	0.09235	0.49982	-0.09963
O	1.16623	-0.33907	0.01560

H	-1.31543	-0.98848	-0.69587
H	-1.39856	-0.57619	1.01593
H	-2.02421	0.55161	-0.19121
H	0.23977	1.52228	0.23036
H	1.97470	0.17367	0.03511

CCOc1ccccc1 Cation Fragment (Dichloromethane)

Number of imaginary frequencies = 0

Electronic energy = -385.321241

Zero-point correction = 0.152280

Enthalpy = -385.160101

Temperature entropy product = 0.038606

Temperature quasi-harmonic entropy product = 0.038240

Gibbs free energy = -385.198707

Quasi-harmonic Gibbs free energy = -385.198341

Cartesian Coordinates

C	-2.47189	1.10699	0.01923
C	-1.61151	-0.09217	0.00046
O	-2.26420	-1.19396	-0.02179
C	-0.17757	-0.04695	0.00234
C	0.59587	-1.22065	0.01255
C	1.96950	-1.14353	0.01095
C	2.59256	0.10035	-0.00175
C	1.84343	1.26913	-0.01122
C	0.46668	1.20054	-0.00787
H	-3.51697	0.81847	0.04486
H	-2.27745	1.70766	-0.87041
H	-2.23278	1.72198	0.88673
H	-1.70991	-1.98613	-0.04321

H	0.14817	-2.20621	0.02732
H	2.56016	-2.04769	0.02052
H	3.67258	0.15562	-0.00331
H	2.33501	2.23085	-0.02074
H	-0.10764	2.11478	-0.01560

CCOc1ccccc1 Cation Fragment (Methanol)

Number of imaginary frequencies = 0

Electronic energy = -385.327775

Zero-point correction = 0.152062

Enthalpy = -385.166752

Temperature entropy product = 0.039230

Temperature quasi-harmonic entropy product = 0.038505

Gibbs free energy = -385.205981

Quasi-harmonic Gibbs free energy = -385.205257

Cartesian Coordinates

C	-2.47043	1.10774	0.01424
C	-1.61201	-0.09260	0.00038
O	-2.26537	-1.19319	-0.01527
C	-0.17726	-0.04796	0.00131
C	0.59624	-1.22132	0.00872
C	1.97012	-1.14317	0.00791
C	2.59266	0.10085	-0.00106
C	1.84285	1.26915	-0.00823
C	0.46601	1.19969	-0.00615
H	-3.51594	0.82052	0.03422
H	-2.26946	1.70900	-0.87327
H	-2.23505	1.72074	0.88418
H	-1.71273	-1.98667	-0.03101

H	0.14889	-2.20691	0.01927
H	2.56099	-2.04721	0.01499
H	3.67265	0.15685	-0.00179
H	2.33344	2.23142	-0.01507
H	-0.10891	2.11349	-0.01212

CCOc1ccccc1 Radical Fragment (Dichloromethane)

Number of imaginary frequencies = 0

Electronic energy = -385.478188

Zero-point correction = 0.148606

Enthalpy = -385.319991

Temperature entropy product = 0.041865

Temperature quasi-harmonic entropy product = 0.040591

Gibbs free energy = -385.361856

Quasi-harmonic Gibbs free energy = -385.360582

Cartesian Coordinates

C	-2.47716	1.13673	-0.00222
C	-1.60842	-0.07148	-0.00048
O	-2.30872	-1.23150	0.00258
C	-0.19985	-0.04153	-0.00050
C	0.59866	-1.21509	-0.00182
C	1.97617	-1.14221	-0.00133
C	2.62752	0.08806	0.00035
C	1.86493	1.25494	0.00147
C	0.48840	1.20082	0.00105
H	-3.52234	0.83636	-0.01167
H	-2.29332	1.76444	-0.87745
H	-2.30744	1.75694	0.88138
H	-1.71999	-1.98812	0.00869

H	0.14896	-2.20087	-0.00411
H	2.55365	-2.05776	-0.00251
H	3.70762	0.13697	0.00071
H	2.35660	2.21940	0.00281
H	-0.07547	2.12327	0.00240

CCOc1ccccc1 Radical Fragment (Methanol)

Number of imaginary frequencies = 0

Electronic energy = -385.480174

Zero-point correction = 0.148829

Enthalpy = -385.321829

Temperature entropy product = 0.041244

Temperature quasi-harmonic entropy product = 0.040224

Gibbs free energy = -385.363073

Quasi-harmonic Gibbs free energy = -385.362053

Cartesian Coordinates

C	-2.53318	1.09932	-0.00005
C	-1.59116	-0.04976	0.00002
O	-2.15369	-1.28741	0.00003
C	-0.18347	-0.00196	0.00001
C	0.58132	-1.19934	-0.00001
C	1.95985	-1.16376	-0.00003
C	2.64527	0.04915	-0.00001
C	1.91453	1.23623	0.00002
C	0.53610	1.22132	0.00003
H	-3.18099	1.06621	-0.88153
H	-2.02310	2.05693	0.00001
H	-3.18116	1.06617	0.88131
H	-3.10903	-1.21296	0.00001

H	0.06920	-2.15054	-0.00001
H	2.51348	-2.09435	-0.00004
H	3.72648	0.06998	-0.00003
H	2.43226	2.18707	0.00003
H	0.00681	2.16357	0.00006

CCc1ccccc1 Cation Fragment (Dichloromethane)

Number of imaginary frequencies = 0

Electronic energy = -349.383819

Zero-point correction = 0.173957

Enthalpy = -349.200212

Temperature entropy product = 0.040534

Temperature quasi-harmonic entropy product = 0.039729

Gibbs free energy = -349.240746

Quasi-harmonic Gibbs free energy = -349.239941

Cartesian Coordinates

C	2.94547	0.30832	0.00029
C	1.78914	-0.57857	-0.00031
C	0.44676	-0.24586	-0.00021
C	-0.50371	-1.30411	-0.00006
C	-1.84667	-1.02685	0.00016
C	-2.26570	0.30205	0.00017
C	-1.35499	1.36196	-0.00004
C	-0.01138	1.10019	-0.00023
H	3.56481	0.05239	0.86669
H	3.56663	0.05181	-0.86459
H	2.72261	1.36863	-0.00009
H	2.02086	-1.63988	-0.00069
H	-0.15162	-2.32688	-0.00004

H	-2.57366	-1.82520	0.00031
H	-3.32538	0.52071	0.00031
H	-1.71420	2.38033	-0.00007
H	0.69644	1.91533	-0.00052

CCc1ccccc1 Cation Fragment (Methanol)

Number of imaginary frequencies = 0

Electronic energy = -349.389782

Zero-point correction = 0.174022

Enthalpy = -349.206107

Temperature entropy product = 0.040626

Temperature quasi-harmonic entropy product = 0.039774

Gibbs free energy = -349.246733

Quasi-harmonic Gibbs free energy = -349.245881

Cartesian Coordinates

C	2.94505	0.30865	0.00001
C	1.78951	-0.57844	0.00000
C	0.44665	-0.24530	-0.00000
C	-0.50287	-1.30371	-0.00000
C	-1.84624	-1.02710	0.00000
C	-2.26589	0.30142	0.00001
C	-1.35553	1.36150	-0.00000
C	-0.01153	1.10033	-0.00001
H	3.56493	0.05172	0.86563
H	3.56478	0.05187	-0.86577
H	2.72146	1.36871	0.00012
H	2.02093	-1.63965	-0.00002
H	-0.14972	-2.32606	-0.00001
H	-2.57274	-1.82592	0.00001

H	-3.32564	0.51964	0.00001
H	-1.71497	2.37978	-0.00001
H	0.69597	1.91578	-0.00002

CCc1ccccc1 Radical Fragment (Dichloromethane)

Number of imaginary frequencies = 0

Electronic energy = -349.558086

Zero-point correction = 0.171818

Enthalpy = -349.376387

Temperature entropy product = 0.041823

Temperature quasi-harmonic entropy product = 0.040971

Gibbs free energy = -349.418210

Quasi-harmonic Gibbs free energy = -349.417358

Cartesian Coordinates

C	-2.94055	0.33436	0.00000
C	-1.82367	-0.64976	-0.00001
C	-0.45807	-0.29920	-0.00000
C	0.54063	-1.30419	-0.00000
C	1.88025	-0.98137	0.00000
C	2.28476	0.35285	0.00000
C	1.32315	1.35940	-0.00000
C	-0.02183	1.04778	-0.00000
H	-2.90286	0.98955	-0.87629
H	-3.90625	-0.16661	-0.00014
H	-2.90302	0.98934	0.87646
H	-2.06784	-1.70490	-0.00000
H	0.23470	-2.34336	0.00000
H	2.62214	-1.76951	0.00001
H	3.33687	0.60373	0.00000

H	1.63111	2.39715	-0.00000
H	-0.75286	1.84540	-0.00001

CCc1ccccc1 Radical Fragment (Methanol)

Number of imaginary frequencies = 0

Electronic energy = -349.558574

Zero-point correction = 0.171845

Enthalpy = -349.376885

Temperature entropy product = 0.041490

Temperature quasi-harmonic entropy product = 0.040826

Gibbs free energy = -349.418375

Quasi-harmonic Gibbs free energy = -349.417711

Cartesian Coordinates

C	2.94071	0.33436	0.00000
C	1.82390	-0.64979	-0.00000
C	0.45818	-0.29919	-0.00000
C	-0.54058	-1.30439	-0.00000
C	-1.88038	-0.98153	0.00000
C	-2.28502	0.35285	0.00000
C	-1.32330	1.35955	-0.00000
C	0.02187	1.04798	-0.00000
H	2.90298	0.98966	0.87620
H	3.90628	-0.16677	-0.00003
H	2.90295	0.98970	-0.87616
H	2.06789	-1.70501	-0.00001
H	-0.23464	-2.34357	0.00000
H	-2.62225	-1.76971	0.00000
H	-3.33715	0.60369	0.00000
H	-1.63124	2.39731	-0.00000

H 0.75282 1.84569 -0.00000

CO Cation Fragment (Dichloromethane)

Number of imaginary frequencies = 0

Electronic energy = -193.583905

Zero-point correction = 0.097661

Enthalpy = -193.480032

Temperature entropy product = 0.030852

Temperature quasi-harmonic entropy product = 0.030755

Gibbs free energy = -193.510883

Quasi-harmonic Gibbs free energy = -193.510786

Cartesian Coordinates

C	-0.61975	0.03279	0.00000
O	0.60827	-0.13063	-0.00000
H	-1.05866	1.02770	-0.00001
H	-1.22231	-0.86979	0.00001
H	1.13332	0.69038	0.00002

CO Cation Fragment (Methanol)

Number of imaginary frequencies = 0

Electronic energy = -193.591625

Zero-point correction = 0.097281

Enthalpy = -193.487991

Temperature entropy product = 0.031391

Temperature quasi-harmonic entropy product = 0.031095

Gibbs free energy = -193.519382

Quasi-harmonic Gibbs free energy = -193.519085

Cartesian Coordinates

C	0.61971	0.03273	-0.00000
O	-0.60844	-0.13076	-0.00000
H	1.05824	1.02733	-0.00000
H	1.22233	-0.86940	0.00001
H	-1.13135	0.69178	0.00002

CO Radical Fragment (Dichloromethane)

Number of imaginary frequencies = 0

Electronic energy = -193.726768

Zero-point correction = 0.094728

Enthalpy = -193.625442

Temperature entropy product = 0.031923

Temperature quasi-harmonic entropy product = 0.031921

Gibbs free energy = -193.657365

Quasi-harmonic Gibbs free energy = -193.657363

Cartesian Coordinates

C	-0.68063	0.02707	-0.05987
O	0.66663	-0.12606	0.01709
H	-1.10726	0.99917	0.14793
H	-1.23661	-0.88228	0.10872
H	1.09461	0.72914	-0.03418

CO Radical Fragment (Methanol)

Number of imaginary frequencies = 0

Electronic energy = -193.727386

Zero-point correction = 0.094724

Enthalpy = -193.626074

Temperature entropy product = 0.031897

Temperature quasi-harmonic entropy product = 0.031896

Gibbs free energy = -193.657972

Quasi-harmonic Gibbs free energy = -193.657971

Cartesian Coordinates

C	-0.68066	0.02702	-0.05971
O	0.66660	-0.12621	0.01654
H	-1.10624	0.99987	0.14624
H	-1.23703	-0.88194	0.11042
H	1.09442	0.72962	-0.03077

Cc1ccccc1 Cation Fragment (Dichloromethane)

Number of imaginary frequencies = 0

Electronic energy = -349.383819

Zero-point correction = 0.173957

Enthalpy = -349.200212

Temperature entropy product = 0.040534

Temperature quasi-harmonic entropy product = 0.039729

Gibbs free energy = -349.240746

Quasi-harmonic Gibbs free energy = -349.239941

Cartesian Coordinates

C	2.33309	0.00000	0.00001
C	0.97359	0.00000	-0.00001
C	0.25317	1.23721	-0.00001
C	-1.11256	1.22669	-0.00000

C	-1.78552	-0.00000	0.00001
C	-1.11255	-1.22669	-0.00000
C	0.25317	-1.23720	-0.00001
H	2.89509	-0.92542	0.00001
H	2.89510	0.92542	0.00001
H	0.80541	2.16704	0.00000
H	-1.67383	2.14885	0.00000
H	-2.86776	-0.00000	0.00003
H	-1.67382	-2.14886	0.00000
H	0.80542	-2.16703	-0.00001

Cc1ccccc1 Cation Fragment (Methanol)

Number of imaginary frequencies = 0

Electronic energy = -349.389782

Zero-point correction = 0.174022

Enthalpy = -349.206107

Temperature entropy product = 0.040626

Temperature quasi-harmonic entropy product = 0.039774

Gibbs free energy = -349.246733

Quasi-harmonic Gibbs free energy = -349.245881

Cartesian Coordinates

C	2.33287	-0.00000	0.00003
C	0.97326	0.00000	-0.00002
C	0.25334	1.23694	-0.00002
C	-1.11250	1.22649	-0.00000
C	-1.78548	-0.00000	0.00002
C	-1.11249	-1.22649	0.00000
C	0.25334	-1.23694	-0.00002
H	2.89403	-0.92581	0.00005

H	2.89404	0.92580	0.00004
H	0.80637	2.16608	-0.00002
H	-1.67360	2.14871	0.00001
H	-2.86765	-0.00000	0.00006
H	-1.67360	-2.14871	0.00001
H	0.80638	-2.16608	-0.00003

Cc1ccccc1 Radical Fragment (Dichloromethane)

Number of imaginary frequencies = 0

Electronic energy = -349.558086

Zero-point correction = 0.171818

Enthalpy = -349.376387

Temperature entropy product = 0.041823

Temperature quasi-harmonic entropy product = 0.040971

Gibbs free energy = -349.418210

Quasi-harmonic Gibbs free energy = -349.417358

Cartesian Coordinates

C	2.38891	0.00000	0.00001
C	0.98780	-0.00000	-0.00001
C	0.25093	1.21157	-0.00001
C	-1.12711	1.20500	0.00000
C	-1.82792	-0.00000	0.00001
C	-1.12711	-1.20499	-0.00000
C	0.25094	-1.21156	-0.00001
H	2.94421	-0.92756	0.00003
H	2.94420	0.92757	0.00001
H	0.78956	2.15098	-0.00002
H	-1.66835	2.14216	0.00001
H	-2.90945	-0.00000	0.00003

H	-1.66835	-2.14216	-0.00000
H	0.78956	-2.15098	-0.00001

Cc1ccccc1 Radical Fragment (Methanol)

Number of imaginary frequencies = 0

Electronic energy = -349.558574

Zero-point correction = 0.171845

Enthalpy = -349.376885

Temperature entropy product = 0.041490

Temperature quasi-harmonic entropy product = 0.040826

Gibbs free energy = -349.418375

Quasi-harmonic Gibbs free energy = -349.417711

Cartesian Coordinates

C	-2.38914	-0.00000	0.00001
C	-0.98787	0.00000	-0.00001
C	-0.25100	-1.21179	-0.00001
C	1.12721	-1.20517	0.00000
C	1.82810	0.00000	0.00001
C	1.12721	1.20517	-0.00000
C	-0.25100	1.21179	-0.00001
H	-2.94417	0.92776	0.00004
H	-2.94417	-0.92776	0.00000
H	-0.78964	-2.15120	-0.00001
H	1.66848	-2.14231	0.00000
H	2.90962	0.00000	0.00002
H	1.66848	2.14231	-0.00000
H	-0.78964	2.15120	-0.00001

References

[1] J. Harnedy, H. A. Maashi, A. A. M. A. El Gehani, M. Burns, L. C. Morrill, *Org Lett* **2023**, *25*, 1486–1490.

[2] F. D. Greene, *J Am Chem Soc* **1959**, *81*, 2688–2691.

[3] A. Gilbert, R. S. Haines, J. B. Harper, *J Phys Org Chem* **2021**, *34*, e4217.

[4] A. L. Berger, K. Donabauer, B. König, *Chem Sci* **2019**, *10*, 10991–10996.

[5] L. Cicco, M. J. Rodríguez-Álvarez, F. M. Perna, J. García-Álvarez, V. Capriati, *Green Chemistry* **2017**, *19*, 3069.

[6] M. Hatano, T. Miyamoto, K. Ishihara, *Org Lett* **2007**, *9*, 4535–4538.

[7] Y. V. Shklyaev, Y. S. Rozhkova, A. N. Perevoshchikova, A. A. Gorbunov, *Russian Chemical Bulletin* **2014**, *63*, 2087–2091.

[8] P. Huang, G. Zhang, Z. Wang, J. Wu, *Chem Asian J* **2023**, *18*, e202300425.

[9] L. Li, X. Wang, N. Fu, *Angewandte Chemie* **2024**, *136*, e202403475.

[10] Q. Y. Li, S. N. Gockel, G. A. Lutovsky, K. S. DeGlopper, N. J. Baldwin, M. W. Bundesmann, J. W. Tucker, S. W. Bagley, T. P. Yoon, *Nature Chemistry 2022 14:1* **2022**, *14*, 94–99.

[11] D. GonzálezGonz, M. A. Morales-Reza, A. Fuentes-BenítesBenítes, C. GonzálezGonz, **2016**, DOI 10.1039/c6ra13088g.

[12] M. J. Frisch, G. W. Trucks, H. B. Schlegel, G. E. Scuseria, M. A. Robb, J. R. Cheeseman, G. Scalmani, V. Barone, G. A. Petersson, H. Nakatsuji, X. Li, M. Caricato, A. V. Marenich, J. Bloino, B. G. Janesko, R. Gomperts, B. Mennucci, H. P. Hratchian, J. V. Ortiz, A. F. Izmaylov, J. L. Sonnenberg, D. Williams-Young, F. Ding, F. Lipparini, F. Egidi, J. Goings, B. Peng, A. Petrone, T. Henderson, D. Ranasinghe, V. G. Zakrzewski, J. Gao, N. Rega, G. Zheng, W. Liang, M. Hada, M. Ehara, K. Toyota, R. Fukuda, J. Hasegawa, M. Ishida, T. Nakajima, Y. Honda, O. Kitao, H. Nakai, T. Vreven, K. Throssell, J. A. Montgomery Jr., J. A., J. E. Peralta, F. Ogliaro, M. J. Bearpark, J. J. Heyd, E. N. Brothers, K. N. Kudin, V. N. Staroverov, T. A. Keith, R. Kobayashi, J. Normand, K. Raghavachari, A. P. Rendell, J. C. Burant, S. S. Iyengar, J. Tomasi, M. Cossi, J. M. Millam, M. Klene, C. Adamo, R. Cammi, J. W. Ochterski, R. L. Martin, K. Morokuma, O. Farkas, J. B. Foresman and D. J. Fox, Gaussian 16, revision C.01, Gaussian Inc.: Wallingford CT, 2016.

[13] C. Cappelli, F. Lipparini, J. Bloino, V. Barone, *Journal of Chemical Physics* **2011**, *135*, DOI 10.1063/1.3630920/983407.

[14] S. Grimme, *Chemistry – A European Journal* **2012**, *18*, 9955–9964.

[15] G. Luchini, R. Paton, J. Alegre-Requena, J. Rodríguez-Guerra, E. Berquist, J. Chen, IFunes, J. Velmiskina, froessler, H. Mayes, S. Sowndarya S. Vejaykummar, sibo, patonlab/GoodVibes, 2022, https://doi.org/10.5281/zenodo.6977304 [accessed May 2024].

[16] GaussView, Version 6.1.1, Roy Dennington, Todd A. Keith, and John M. Millam, Semichem Inc., Shawnee Mission, KS, 2016.

[17] CYLview, 1.0b; C. Y. Legault, Université de Sherbrooke, 2009, http://www.cylview.org.

[18] G. Landrum, P. Tosco, B. Kelley, Ric, sriniker, D. Cosgrove, gedeck, R. Vianello, N. Schneider, E. Kawashima, D. N, G. Jones, A. Dalke, B. Cole, M. Swain, S. Turk, A. Savelyev, A. Vaucher, M. Wójcikowski, I. Take, D. Probst, K. Ujihara, V. F. Scalfani, guillaume godin, A. Pahl, F. Berenger, JLVarjo, R. Walker, jasondbiggs, strets123, RDKit: Open-source cheminformatics., 2024, https://rdkit.org [accessed May 2024].
